# SCRIPT: Predicting Single‐Cell Long‐Range Cis‐Regulation Based on Pretrained Graph Attention Networks

**DOI:** 10.1002/advs.202505021

**Published:** 2025-08-20

**Authors:** Yu Zhang, Baole Wen, Yifeng Jiao, Yuchen Liu, Xin Guo, Yushuai Wu, Jiyang Li, Limei Han, Yinghui Xu, Xin Gao, Yuan Qi, Yuan Cheng, Ying He, Weidong Tian

**Affiliations:** ^1^ Artificial Intelligence Innovation and Incubation Institute Fudan University Shanghai 200433 China; ^2^ Shanghai Academy of Artificial Intelligence for Science Shanghai 200232 China; ^3^ INF Technology (Shanghai) Co. Ltd Shanghai 200232 China; ^4^ State Key Laboratory of Genetics and Development of Complex Phenotypes Department of Computational Biology School of Life Sciences Fudan University Shanghai 200438 China; ^5^ Computer Electrical and Mathematical Sciences and Engineering Division King Abdullah University of Science and Technology (KAUST) Thuwal 23955‐6900 Saudi Arabia; ^6^ Center of Excellence for Smart Health King Abdullah University of Science and Technology (KAUST) Thuwal 23955‐6900 Saudi Arabia; ^7^ Center of Excellence on GenAI King Abdullah University of Science and Technology (KAUST) Thuwal 23955‐6900 Saudi Arabia; ^8^ Zhongshan Hospital Fudan University Shanghai 200032 China; ^9^ Children's Hospital of Fudan University Shanghai 201102 China; ^10^ Children's Hospital of Shandong University Jinan 250022 China

**Keywords:** cis‐regulation, graph neural networks, non‐coding variant, pretrain, single cell

## Abstract

Single‐cell cis‐regulatory relationships (CRRs) are essential for deciphering transcriptional regulation and understanding the pathogenic mechanisms of disease‐associated non‐coding variants. Existing computational methods struggle to accurately predict single‐cell CRRs due to inadequately integrating causal biological principles and large‐scale single‐cell data. Here, SCRIPT (Single‐cell Cis‐regulatory Relationship Identifier based on Pre‐Trained graph attention networks) is presented for inferring single‐cell CRRs from transcriptomic and chromatin accessibility data. SCRIPT incorporates two key innovations: graph causal attention networks supported by empirical CRR evidence, and representation learning enhanced through pretraining on atlas‐scale single‐cell chromatin accessibility data. Validation using cell‐type‐specific chromatin contact and CRISPR perturbation data demonstrates that SCRIPT achieves a mean AUC of 0.89, significantly outperforming state‐of‐the‐art methods (AUC: 0.7). Notably, SCRIPT obtains an over twofold improvement in predicting long‐range CRRs (>100 Kb) compared to existing methods. By applying SCRIPT to Alzheimer's disease and schizophrenia, a framework is established for prioritizing disease‐causing variants and elucidating their functional effects in a cell‐type‐specific manner. By uncovering molecular genetic mechanisms undetected by existing computational methods, SCRIPT provides a roadmap for advancing genetic diagnosis and target discovery.

## Graphical abstract

SCRIPT is a novel method inferring single‐cell cis‐regulatory relationships (CRRs) from transcriptomic and chromatin accessibility data. SCRIPT incorporates two key innovations: graph causal attention networks supported by empirical CRR evidence, and representation learning enhanced through pretraining on atlas‐scale single‐cell data. For complex genetic diseases, SCRIPT facilitates prioritizing disease‐causing variants and elucidating their functional effects in a cell‐type‐specific manner.

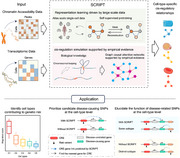

## 1 Introduction

The human genome harbors an extensive repertoire of *cis*‐regulatory elements (CREs) which are preferentially located in accessible chromatin regions, orchestrating precise spatial and temporal patterns of gene expression.^[^ ^1^ ^]^ A major mechanism by which CREs, such as enhancers, act on a target gene is through chromosomal looping, bringing distal enhancers into close physical proximity with their target genes within 3D chromatin space.^[^ ^2^ ^]^ Notably, the *cis*‐regulatory relationships (CRRs) between CREs and their corresponding target genes exhibit two defining characteristics: 1) CRRs are highly cell‐type‐specific, reflecting the unique transcriptional programs of distinct cellular contexts^[^ ^3^ ^]^; and 2) for more than half of CRRs, the genomic distances between CREs and their target genes exceed 100 kilobases (Kb).^[^ ^4^ ^]^ Consequently, single‐cell‐resolution CRR data, particularly those capturing long‐range CRRs, is essential for understanding the regulatory mechanism underlying cell‐type‐specific gene expression.^[^ ^3^, ^5^ ^]^ Moreover, genome‐wide association studies (GWASs) have identified numerous variants associated with complex diseases,^[^ ^6^ ^]^ the majority of which are located in non‐coding regions.^[^ ^7^ ^]^ Single‐cell long‐range CRR data can link more non‐coding variants to their target genes in a cell‐type‐specific manner, providing deeper insights into the genetic underpinnings of complex diseases.^[^ ^5^, ^8^ ^]^

Extensive CRR information can be obtained through the high‐throughput chromosome conformation capture (Hi‐C) technique^[^ ^9^ ^]^ and expression quantitative trait loci (eQTLs) analysis.^[^ ^10^ ^]^ However, these datasets are predominantly available at the tissue level rather than the single‐cell level. To address this limitation, single‐cell Hi‐C (scHi‐C) technologies have been developed to generate single‐cell CRR data.^[^ ^11^ ^]^ However, their high cost and the limited availability of publicly accessible scHi‐C datasets hinder widespread adoption of them.

To circumvent this limitation, computational methods have been developed to predict CRRs. Some approaches, such as Enformer^[^ ^12^ ^]^ and GraphReg,^[^ ^13^ ^]^ utilize DNA sequence features and epigenomic profiles from the ENCODE project^[^ ^14^ ^]^ to predict enhancer‐gene interactions. However, due to their reliance on tissue‐level or cell‐line‐level data available in ENCODE, these methods cannot generalize across diverse cell types or capture CRRs at single‐cell resolution. Other approaches, including SCENIC+,^[^ ^15^ ^]^ FigR,^[^ ^16^ ^]^ LINGER,^[^ ^17^ ^]^ and SCARLink,^[^ ^18^ ^]^ leverage the growing abundance of single‐cell assays for transposase‐accessible chromatin using sequencing (scATAC‐seq) and single‐cell RNA sequencing (scRNA‐seq) data to infer CRRs at single‐cell resolution. LINGER and SCARLink are considered state‐of‐the‐art (SOTA) tools for single‐cell CRR prediction, as they have demonstrated superior performance over other existing methods in previous benchmark evaluations.^[^ ^15^, ^17^, ^18^ ^]^ Despite their strengths, both tools still face critical challenges. SCARLink primarily infers CRRs by modeling associations between chromatin accessibility and gene expression. In contrast, LINGER integrates transcription factor (TF) expression and chromatin accessibility, while also leveraging large‐scale external bulk datasets and TF binding motif information as regularization to improve gene expression prediction. However, neither method explicitly integrates prior knowledge of cis‐regulatory mechanisms to enhance causal interpretability, which may limit their ability to accurately identify long‐range CRRs. In addition, they utilize a limited amount of single‐cell data for model training rather than exploit the wealth of large‐scale publicly available single‐cell atlas datasets. The limited training data are not enough to learn complex single‐cell *cis‐*regulatory mechanisms.

To address these challenges, we propose a novel deep‐learning‐based method called SCRIPT (Single‐cell *Cis‐*regulatory Relationship Identifier based on Pre‐Trained graph attention networks). SCRIPT incorporates two key algorithmic innovations. First, it leverages graph causal attention networks (GCATs) to simulate *cis‐*transcriptional regulation, using chromatin accessibility of CREs measured by scATAC‐seq to predict gene expression levels quantified by scRNA‐seq via CRRs. Causal attention masks of GCATs^[^ ^19^, ^20^ ^]^ are designed by incorporating empirical evidence from large‐scale bulk Hi‐C and eQTL datasets, enabling the modeling of *cis*‐transcriptional regulation grounded in biological principles. Second, SCRIPT employs a self‐supervised graph autoencoder (SSGAE)^[^ ^21^ ^]^ pretrained on atlas‐scale scATAC‐seq data to comprehend the complex interactions between CREs across diverse tissues. This pretraining enables the model to generate effective CRE representations. Recent advancements in foundation models for single‐cell transcriptomics prove the effectiveness of the pretraining strategy.^[^ ^22^, ^23^ ^]^ Relying on these innovative algorithm designs, SCRIPT exhibits excellent performance in predicting single‐cell CRRs, as validated by cell‐type‐specific chromatin contact data and CRISPR perturbation data. Compared to current computational methods, SCRIPT identifies disease‐causing variants more accurately and explains their functional effects more reasonably at the cell type level. Furthermore, we provide a SCRIPT‐based framework to unravel the pathogenic mechanisms underlying the non‐coding variants associated with complex diseases, including Alzheimer's disease (AD) and schizophrenia (SCZ). SCRIPT holds promise in advancing genetic diagnosis and therapeutic target discovery for various complex genetic diseases.

## 2 Results

### 2.1 The Overview of SCRIPT

**Figure 1:**
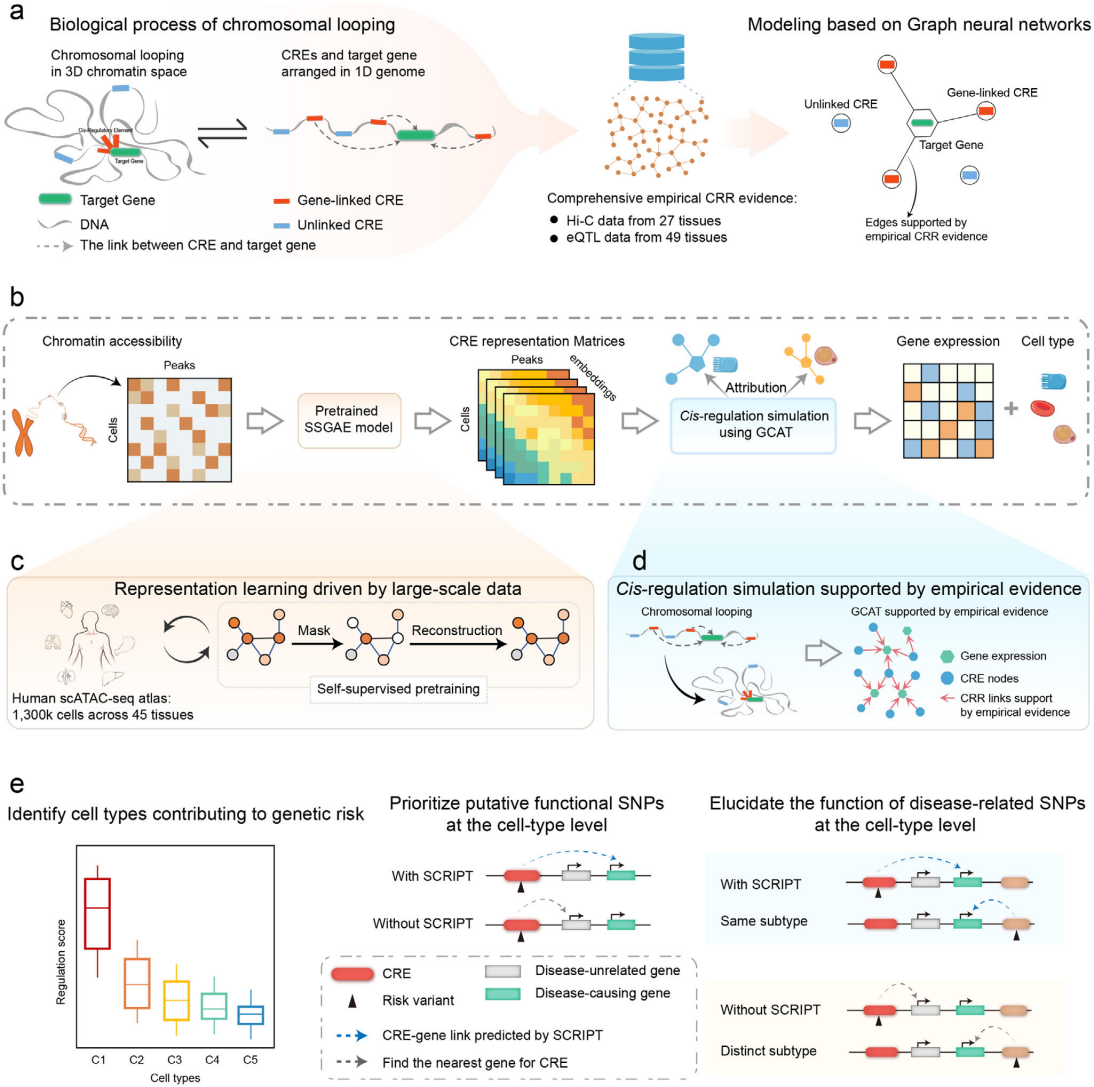
Overview of SCRIPT. a) Graph neural networks inspired by biological principles. SCRIPT incorporates biological principles into graph neural networks to simulate *cis*‐regulatory mechanisms. The biological principles are from comprehensive empirical CRR evidence composed of large‐scale Hi‐C and eQTL datasets. b) Schematic illustration of SCRIPT. SCRIPT uses chromatin accessibility data as input to predict gene expression and cell type, leveraging the pretrained SSGAE and GCAT modules. Single‐cell regulation scores are generated from the GCAT module using the attribution method. c) Representation learning driven by large‐scale data. The SSGAE is pretrained on atlas‐scale scATAC‐seq datasets, enabling the generation of biologically meaningful CRE representations. d) *Cis*‐regulation simulation supported by empirical evidence. GCAT is designed to mimic chromosomal looping to utilize the CRE representations to predict gene expression. e) Applications in disease biology. SCRIPT facilitates the identification of cell types contributing to genetic risk, prioritization of candidate disease‐causing variants, and elucidation of the function of disease‐related variants. CRR, *cis‐*regulatory relationship; CRE, *cis‐*regulatory element; Hi‐C, high‐throughput chromosome conformation capture; eQTL, expression quantitative trait loci; SSGAE; self‐supervised graph autoencoder; GCAT, graph causal attention network; scATAC‐seq, single‐cell assay for transposase‐accessible chromatin using sequencing.

The biological basis of *cis*‐regulation is chromosomal looping, wherein distal CREs are brought into close physical proximity with their target genes within 3D space. The core concept of SCRIPT is incorporating the biological principles of *cis*‐regulation into graph neural networks to simulate *cis*‐regulatory mechanisms. The biological principles are from comprehensive empirical CRR evidence composed of large‐scale tissue‐level Hi‐C (from 27 human tissues) and eQTL (from 49 human tissues) datasets (**Figure** **1**a).

The *cis*‐regulation simulation of SCRIPT leverages chromatin accessibility data to predict gene expression and cell type. Regulation scores for single‐cell CRRs are inferred through an attribution method applied to the trained simulation model, which assesses the impact of each CRE‐gene link on its corresponding gene. SCRIPT's accurate predictions are driven by two innovative components: the pretrained SSGAE model and GCAT supported by empirical evidence (Figure 1b; for details, see Experimental Section and Figure S1, Supporting Information).

SSGAE is pretrained on atlas‐scale human scATAC‐seq data (≈1.3 million cells), enabling it to generate biologically meaningful representations for previously unseen scATAC‐seq datasets (Figure S2, Supporting Information). During pretraining, SSGAE randomly masks node features in the input data and encodes the corrupted graph into node embeddings using an encoder. A decoder then reconstructs the masked input node features. By pretraining on large‐scale scATAC‐seq data, the SSGAE gains a fundamental understanding of *cis*‐regulatory networks, encoding the network hierarchy into the model's attention weights in a completely self‐supervised manner (Figure 1c).

The algorithmic design of GCAT mimics chromosomal looping to utilize the CRE representations to predict gene expression. During GCAT training, attention scores of edges representing CRE‐gene links, supported by empirical evidence, are updated directionally from CREs to target genes. GCAT enables SCRIPT to predict single‐cell CRRs based on biological principles, rather than simple data correlations (Figure 1d).

SCRIPT provides a new avenue for exploring the pathogenic mechanisms of complex diseases. Its applications include the identification of cell types contributing to genetic risk, prioritization of candidate disease‐causing variants, and elucidation of the function of disease‐related variants (Figure 1e).

### 2.2 The Performance Improvement of SCRIPT Benefits from Pretraining on Atlas‐Scale Single Cell Data

To evaluate SCRIPT's performance, we curate five pairs of scATAC‐seq and scRNA‐seq datasets derived from brain, blood, and cancer cell lines that are not included in the pretraining data. Cell‐type‐specific chromatin contact data (proximity ligation‐assisted chromatin immunoprecipitation sequencing (PLAC‐seq)^[^ ^24^ ^]^ and cell‐type‐specific promoter capture Hi‐C (pcHi‐C)^[^ ^25^ ^]^) and CRISPR perturbation data (CRISPR interference (CRISPRi)^[^ ^26^, ^27^ ^]^) are regarded as the true labels for cell‐type‐specific CRR predictions. To clarify the relationship between the empirical CRR evidence set used for graph construction and the CRR set used for model evaluation, we quantify the degree of overlap between these two sets. We find that over 95% of the CRRs in the evaluation set are included in the empirical evidence set (Figure S3a, Supporting Information), highlighting the comprehensiveness of the CRR collection we assembled. Importantly, these overlapping CRRs account for less than 5% of the total empirical CRR evidence set (Figure S3b, Supporting Information), indicating that they comprise only a small fraction of the full empirical CRR evidence set. This ensures that the model cannot rely solely on memorizing specific links to accurately predict cell‐type‐specific CRRs during evaluation.

Four metrics are employed for comparative analysis across different computational methods: cell‐level area under the receiver operating characteristic curve (AUC), cell‐level area under the precision‐recall curve (AUPR) ratio, reg‐level AUC, and reg‐level AUPR ratio. The AUPR ratio is a normalized metric based on AUPR, which serves as a valuable complement to AUC for the class‐imbalanced task of CRR prediction. Moreover, it provides greater discriminative power than AUPR when comparing the performance of different methods (for details, see Materials and Methods). Cell‐level metrics measure the ability to accurately identify cell‐type‐specific CRRs, whereas reg‐level metrics evaluate the accuracy of assigning a cell‐type‐specific CRR to the correct cell type (for details, see Experimental Section and Figure S4, Supporting Information).

To investigate the effectiveness of pretraining on atlas‐scale single‐cell data in CRR prediction, we perform an ablation study by comparing three versions of the method: SCRIPT, SCRIPT pretrained on a small dataset (SCRIPT‐POSD), and SCRIPT without pretraining (SCRIPT‐WOP). In SCRIPT‐POSD, the SSGAE component is pretrained on a substantially smaller scATAC‐seq dataset, whereas SCRIPT‐WOP is trained without any pretraining. In this ablation study, SCRIPT‐WOP serves as a baseline to evaluate the general benefit of pretraining, while SCRIPT‐POSD enables examination of how the scale of pretraining data influences performance.

**Figure 2:**
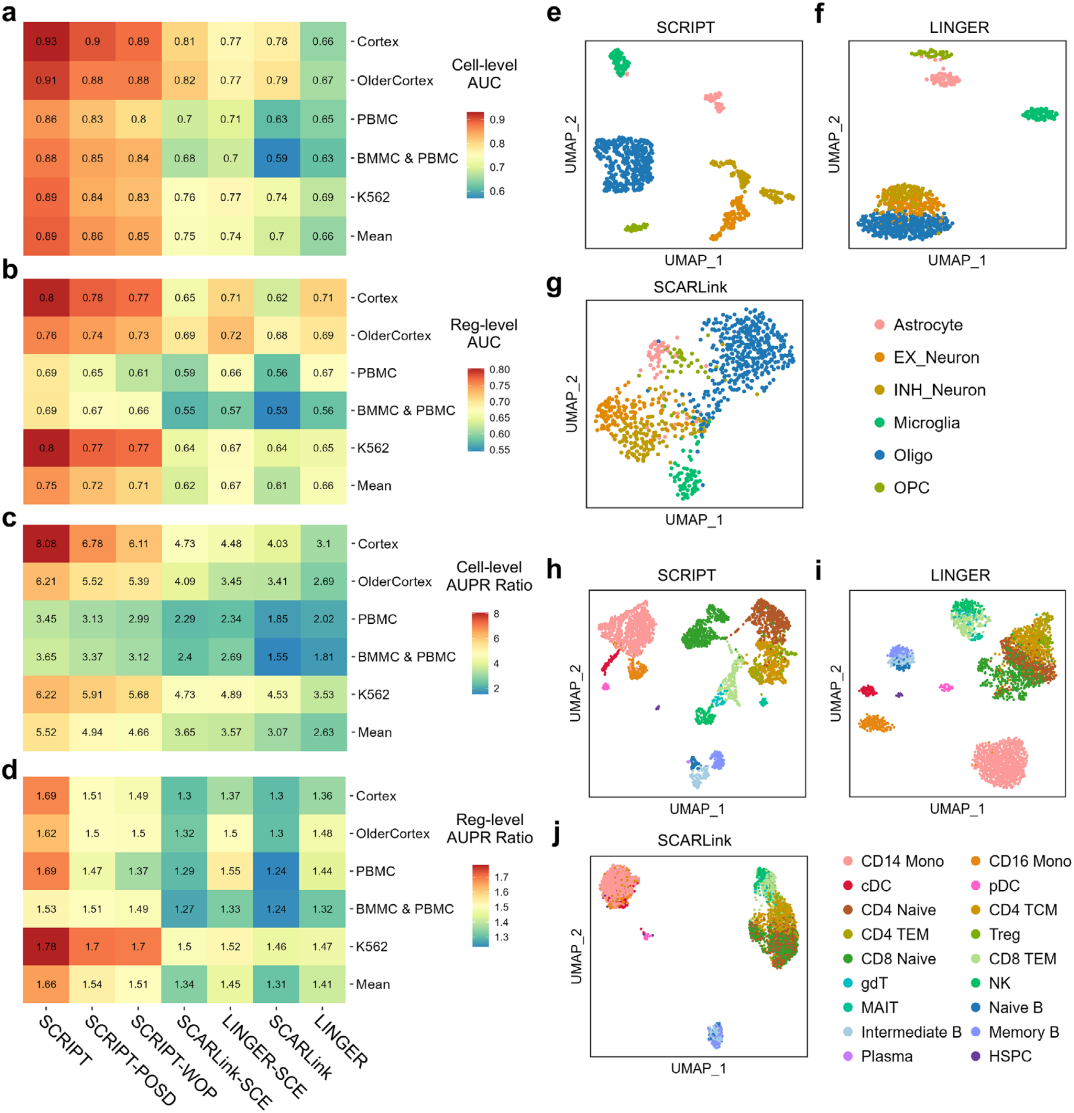
Performance comparison of SCRIPT and competing methods for CRR prediction. a–d) Heatmaps showing cell‐level AUCs (a), reg‐level AUCs (b), cell‐level AUPR ratios (c) and reg‐level AUPR ratios (d) of seven methods for single‐cell CRR prediction across five benchmark datasets. Method names and dataset names are shown at the bottom and left of the heatmaps, respectively. e–g) UMAP visualization of single‐cell regulation score matrices predicted by SCRIPT (e), LINGER (f), and SCARLink (g) for the Cortex dataset (*n* = 852 cells). h‐j) UMAP visualization of single‐cell regulation score matrices predicted by SCRIPT (h), LINGER (i), and SCARLink (j) for the PBMC dataset (*n* = 3609 cells). PBMC, peripheral blood mononuclear cells; BMMC, human bone marrow mononuclear cells.

Relative to SCRIPT‐WOP, SCRIPT‐POSD yields modest improvements: 0.01 in both cell‐level and reg‐level AUC, 0.28 in cell‐level AUPR ratio, and 0.03 in reg‐level AUPR ratio. In contrast, SCRIPT, pretrained on atlas‐scale data, achieves substantially higher gains: 0.04 in both AUC metrics, 0.86 in cell‐level AUPR ratio, and 0.15 in reg‐level AUPR ratio (**Figure** **2**a–d; Figures S5–S8, Supporting Information). These results indicate that while pretraining on a small dataset provides minor improvements, large‐scale pretraining significantly enhances model performance.

To further examine the source of these gains, we evaluate the accuracy of gene expression prediction during the cis‐regulation simulation stage in the ablation study. The results suggest that the performance gains from atlas‐scale pretraining may stem from enhanced representation learning, which leads to more accurate gene expression prediction during the cis‐regulation simulation stage (Figure S9, Supporting Information).

### 2.3 SCRIPT Outperforms Existing Methods in Cell‐Type‐Specific CRR Prediction

We benchmark SCRIPT against two SOTA methods, LINGER and SCARLink. SCRIPT achieves an average cell‐level AUC of 0.89, an average reg‐level AUC of 0.75, an average cell‐level AUPR ratio of 5.52, and an average reg‐level AUPR ratio of 1.66. In comparison, LINGER and SCARLink attain average cell‐level AUCs of 0.66 and 0.7, reg‐level AUCs of 0.66 and 0.61, average cell‐level AUPR ratios of 2.63 and 3.07, and reg‐level AUPR ratios of 1.41 and 1.31, respectively (Figure 2a–d; Figures S5–S8, Supporting Information). These results demonstrate that SCRIPT significantly outperforms both LINGER and SCARLink in both cell‐type‐specific CRR prediction accuracy and robustness across diverse cell types.

To investigate whether empirical CRR evidence can also improve other methods, we modify LINGER and SCARLink by replacing their original distance‐based candidate CREs with the CRR evidence‐based CREs used in SCRIPT. These modified versions, LINGER‐SCE (LINGER supported by CRR evidence) and SCARLink‐SCE (SCARLink supported by CRR evidence), exhibit improved performance. Specifically, the cell‐level AUC increases from 0.70 to 0.74 for SCARLink‐SCE and from 0.66 to 0.75 for LINGER‐SCE; the cell‐level AUPR ratio increases from 3.07 to 3.65 and from 2.63 to 3.57, respectively (Figure 2a–d; Figures S5–S8, Supporting Information). For reg‐level AUC, both LINGER‐SCE and SCARLink‐SCE show modest gains of 0.01, and reg‐level AUPR ratio slightly improves by 0.04 and 0.03, respectively (Figure 2a–d; Figures S5–S8, Supporting Information). Despite these gains, both versions still underperform compared to SCRIPT (Figure 2a–d), suggesting that SCRIPT's superior performance cannot be solely attributed to the inclusion of empirical CRR evidence, but also results from its dedicated model architecture.

Subsequently, single‐cell regulation score matrices generated by the three methods are visualized using Uniform Manifold Approximation and Projection (UMAP) to assess their ability to characterize biological variations inherent in single‐cell data. UMAP visualizations in the cortex dataset reveal that SCRIPT separates distinct cell types, whereas LINGER fails to distinguish excitatory neurons from inhibitory neurons, and SCARLink produces the least coherent cell‐type groupings (Figure 2e–g). Similar results are observed in the peripheral blood mononuclear cells (PBMC) dataset (Figure 2h–j). Overall, SCRIPT outperforms both LINGER and SCARLink in its ability to capture meaningful biological signals.

### 2.4 SCRIPT Demonstrates Superior Long‐Range CRR Prediction

Predicting long‐range CRRs that span over 100 kb is generally considered challenging. We systematically evaluate the ability of SCRIPT, LINGER, and SCARLink to predict long‐range CRRs and visualize their predictions at specific gene loci for direct comparison.

**Figure 3:**
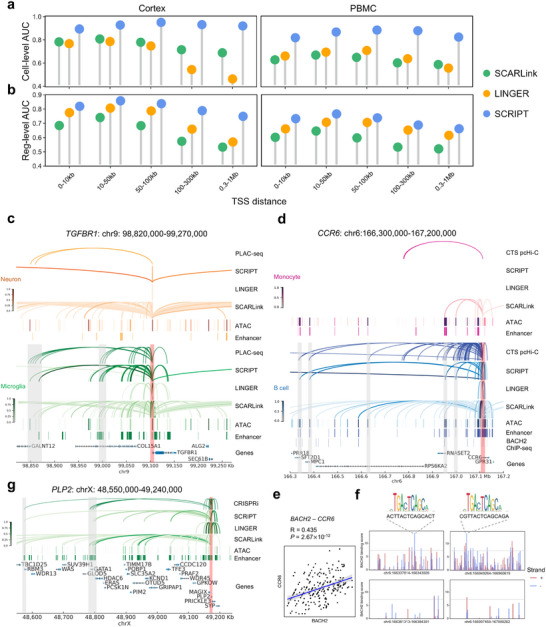
SCRIPT outperforms LINGER and SCARLink in long‐range CRR prediction. a,b) Lollipop plots showing the cell‐level (a) and reg‐level (b) AUCs for cell‐type‐specific CRR prediction in the cortex and PBMC datasets. CRRs are categorized into five distance groups ranging from 0–10Kb to 0.3–1Mb. c,d,g) Genomic visualizations of CRRs identified by PLAC‐seq (c), cell‐type‐specific pcHi‐C (d), or CRISPRi (g) data; normalized regulation scores predicted by SCRIPT, LINGER, and SCARLink; normalized scATAC‐seq‐derived pseudobulk tracks; and enhancers identified by H3K27ac ChIP‐seq data. These examples correspond to the *TGFBR1* gene locus (chr9: 98820000‐99270000) (c), *CCR6* gene locus (chr6:166300000‐167200000) (d) and *PLP2* gene locus (chrX: 48550000‐49240000) (g). The scATAC‐seq tracks represent the aggregate chromatin accessibility of all cells from each given cell type and have been normalized for comparability. The CREs linked to *TGFBR1, CCR6* or *PLP2* based on ChIP‐seq and either PLAC‐seq, cell‐type‐specific pcHi‐C or CRISPRi are highlighted in grey bars. The TSSs of *TGFBR1, CCR6* and *PLP2* are marked by red bars. In B cell, BACH2 ChIP‐seq track is also shown. e) Dot plots showing the expression of *BACH2* in B cells and its relationship with *CCR6* (n = 234 cells) expression. Only cells with non‐zero expression for the corresponding gene (i.e., no dropout) are shown. The blue line denotes the linear regression fitted to the points, and the shaded grey area indicates the 95% confidence interval. Two‐sided t‐test is used to evaluate the statistical significance of the correlation. f) Bar plots showing the BACH2 binding scores across the enhancer regions chr6:166337814‐166343926, chr6:166381313‐166384391, chr6:166949264‐166960619 and chr6:166997459‐167000262. Red and blue bars represent predicted binding scores on the sense and antisense DNA strands, respectively. The grey horizontal line indicates the JASPAR‐recommended threshold for high‐confidence TFBS predictions. For chr6:166337814‐166343926 and chr6:166949264‐166960619, the sequences with the highest predicted binding score are respectively highlighted, and the corresponding BACH2 motif is displayed. CTS pcHi‐C, cell‐type‐specific promoter capture Hi‐C; TFBS, transcription factor binding site.

First, CRRs are stratified into groups based on the distances between each CRE and its corresponding target gene's transcription start site (TSS). In the cortex dataset, for three CRR groups spanning less than 100 kb, SCRIPT achieves a mean cell‐level AUC of 0.92, and a reg‐level AUC of 0.84, both of which outperform LINGER and SCARLink (highest cell‐level AUC: 0.79, reg‐level AUC: 0.79) (**Figure** **3**a,b). Notably, SCRIPT maintains robust performance in long‐range CRR groups (100–300 kb and 0.3–1Mb), achieving a mean cell‐level AUC of 0.90 and a mean reg‐level AUC of 0.75. In contrast, LINGER's mean cell‐level AUC significantly drops to 0.5 and its mean reg‐level AUC to 0.62, while SCARLink's mean cell‐level AUC declines to 0.7 and its mean reg‐level AUC to 0.55 (Figure 3a,b). Considering that a random classifier would yield an AUC of 0.5, SCRIPT demonstrates a twofold relative improvement in both cell‐level and reg‐level AUC compared to LINGER and SCARLink for long‐range CRR prediction.

SCRIPT's superiority in long‐range CRR prediction is further evident in the PBMC dataset, where it attains a mean cell‐level AUC of 0.85 for long‐range CRR groups (100–300 kb and 0.3–1Mb). This performance represents a threefold relative improvement over the mean cell‐level AUC of 0.6 observed for LINGER and SCARLink (Figure 3a,b). Besides, when evaluated using AUPR ratio, SCRIPT consistently demonstrates superior performance in long‐range CRR prediction (Figure S10, Supporting Information). These findings underscore SCRIPT's superior capacity to accurately identify and characterize long‐range CRRs.

To directly compare the long‐range CRR prediction performance of SCRIPT with that of LINGER and SCARLink, we visualize the CRRs identified by experimental data alongside the predictions of the three methods.

As a case study in the cortex dataset, we examine the *TGFBR1* locus, a microglia marker gene critical for microglial maturation.^[^ ^28^ ^]^ In microglia, PLAC‐seq data^[^ ^24^ ^]^ identify two distal sets of CREs regulating *TGFBR1*, located ≈250 kb upstream, 105 kb upstream from the TSS of *TGFBR1* (highlighted by the grey bars in Figure 3c). SCRIPT accurately identifies both CRE sets (Figure 3c; Table S1, Supporting Information), whereas LINGER fails to detect either (Figure 3c). Although SCARLink identifies both CRE sets, it produces numerous false‐positive predictions. Particularly, SCARLink predicts many CRRs in neurons that are not corroborated by PLAC‐seq data, highlighting its reduced specificity compared to SCRIPT (Figure 3c). Moreover, H3K27ac is a histone modification associated with the higher activation of transcription and therefore considered as an enhancer mark.^[^ ^29^ ^]^ The CREs identified by SCRIPT to regulate *TGFBR1* are consistently labeled as microglia‐specific enhancers in H3K27ac ChIP‐seq data.^[^ ^24^ ^]^ In contrast, many CREs identified by SCARLink fail to align with H3K27ac ChIP‐seq data, further emphasizing its tendency to generate false positives (Figure 3c; Figure S11, Supporting Information).

Another representative example in the PBMC dataset further displays SCRIPT's advantages in long‐range CRR prediction. *CCR6*, a well‐established marker gene for B cells, plays an important role in the cell fate transition of B cells.^[^ ^30^ ^]^ Cell‐type‐specific pcHi‐C and H3K27ac ChIP‐seq data^[^ ^25^, ^31^ ^]^ identify five remote candidate enhancer sets regulating *CCR6*, located ≈775 kb, 730 kb, 480 kb, 165 kb, and 125 kb upstream of the TSS of *CCR6* (highlighted by the grey bars in Figure 3d). SCRIPT successfully detects four of these enhancer sets (Figure 3d; Table S2 and Figure S12, Supporting Information), whereas SCARLink identifies only two, and LINGER fails to identify any (Figure 3d; Figure S12, Supporting Information). Additionally, SCARLink predicted numerous false‐positive CRRs in monocytes, further emphasizing its reduced specificity relative to SCRIPT (Figure 3d; Figure S12, Supporting Information).

Previous studies have shown that the generation of *CCR6* ^+^ B cells requires the expression of BACH2.^[^ ^30^, ^32^ ^]^ Besides, we observe a significant co‐expression pattern between *BACH2* and *CCR6* in B cells (Figure 3e), suggesting that BACH2 may positively regulate *CCR6* expression in B cells. We examine the four distal enhancers predicted by SCRIPT to be linked to *CCR6* for their potential to bind BACH2. Two of these enhancers exhibit BACH2 ChIP‐seq signals in B cells^[^ ^33^ ^]^ (Figure 3d), and all four contain predicted transcription factor binding sites (TFBSs) of BACH2 (Figure 3f). These findings further support the literature‐based hypothesis that BACH2 may regulate *CCR6* expression through direct binding to its distal enhancers.

Besides, an exploratory result is observed that SCRIPT identifies a B cell‐specific enhancer linked to *CCR6* located 16.5 Mb upstream of the *CCR6* TSS (Figure S13, Supporting Information). Although exploratory results predicted by SCRIPT require further experimental validation, emerging studies have demonstrated the existence of ultra‐long‐range CRRs^[^ ^34^, ^35^ ^]^ and these results represent testable hypotheses that may inform future functional investigations.

In addition, in the cancer cell line dataset, *PLP2*, a gene implicated in cancer cell proliferation and migration,^[^ ^36^ ^]^ is regulated by two distal enhancer sets validated through CRISPRi^[^ ^26^, ^27^ ^]^ and H3K27ac ChIP‐seq data^[^ ^37^ ^]^ in K562 cell line. These sets are located ≈600 kb and 380 kb upstream of the TSS of *PLP2*, respectively, and are highlighted by the grey bars in Figure 3g. SCRIPT accurately identifies both enhancer sets, whereas LINGER fails to detect either (Figure 3g; Figure S14 and Table S3, Supporting Information). Although SCARLink is able to recover both enhancer sets, it also produces a substantially higher number of false‐positive predictions (Figure 3g; Figure S14, Supporting Information). Similar trends are observed at other gene loci, such as *PIK3R5*, *EFNA5*, *ENPP2*, *CDCA7L*, *IRAK3*, *SYTL2*, and *MAL* (Figures S15–S18, Supporting Information).

Taken together, these results underline SCRIPT's superior capacity to identify long‐range CRRs with high precision and specificity, outperforming both SCARLink and LINGER.

### 2.5 SCRIPT Attends to Cell‐Type‐Specific Enhancers

To determine whether CREs identified by SCRIPT are preferentially regarded as enhancers, we examine the overlap between enhancers labeled by H3K27ac ChIP‐seq data and CRRs predicted by SCRIPT. We find that those CRRs with astrocyte‐specific enhancers exhibit significantly higher regulation scores in astrocytes compared to the other cell types (Figure S19a, Supporting Information). Similar trends are observed for enhancers specific to microglia, neurons, and oligodendrocytes (Figure S19b–d, Supporting Information). In addition, we identify marker CRRs and marker genes for each cell type through differential analysis of the regulation score matrix and the gene expression matrix. The analysis shows a significant concordance between genes regulated by marker CRRs and marker genes for the same cell type (Figure S19e, Supporting Information). These results suggest that CRRs with high regulation scores are likely to promote the expression of their corresponding target genes, strongly indicating that SCRIPT prioritizes the identification of cell‐type‐specific enhancer‐like CREs. Similar patterns are observed in the PBMC dataset (Figure S20, Supporting Information).

### 2.6 SCRIPT Understands the Pathogenic Mechanisms of AD and SCZ in a Cell‐Type‐Specific Manner

Both AD and SCZ are complex genetic diseases characterized by progressive cognitive impairment and structural alteration in the brain. GWASs conducted by Iris E. Jansen et al.^[^ ^38^ ^]^ and Antonio F. Pardiñas et al.^[^ ^39^ ^]^ have identified 275 AD‐associated and 1895 SCZ‐associated single nucleotide polymorphisms (SNPs) mapped to CREs identified through scATAC‐seq data in the human brain^[^ ^40^ ^]^ (Tables S4 and S5, Supporting Information). Notably, the majority of these SNPs are located in non‐coding regions of the human genome.

**Figure 4:**
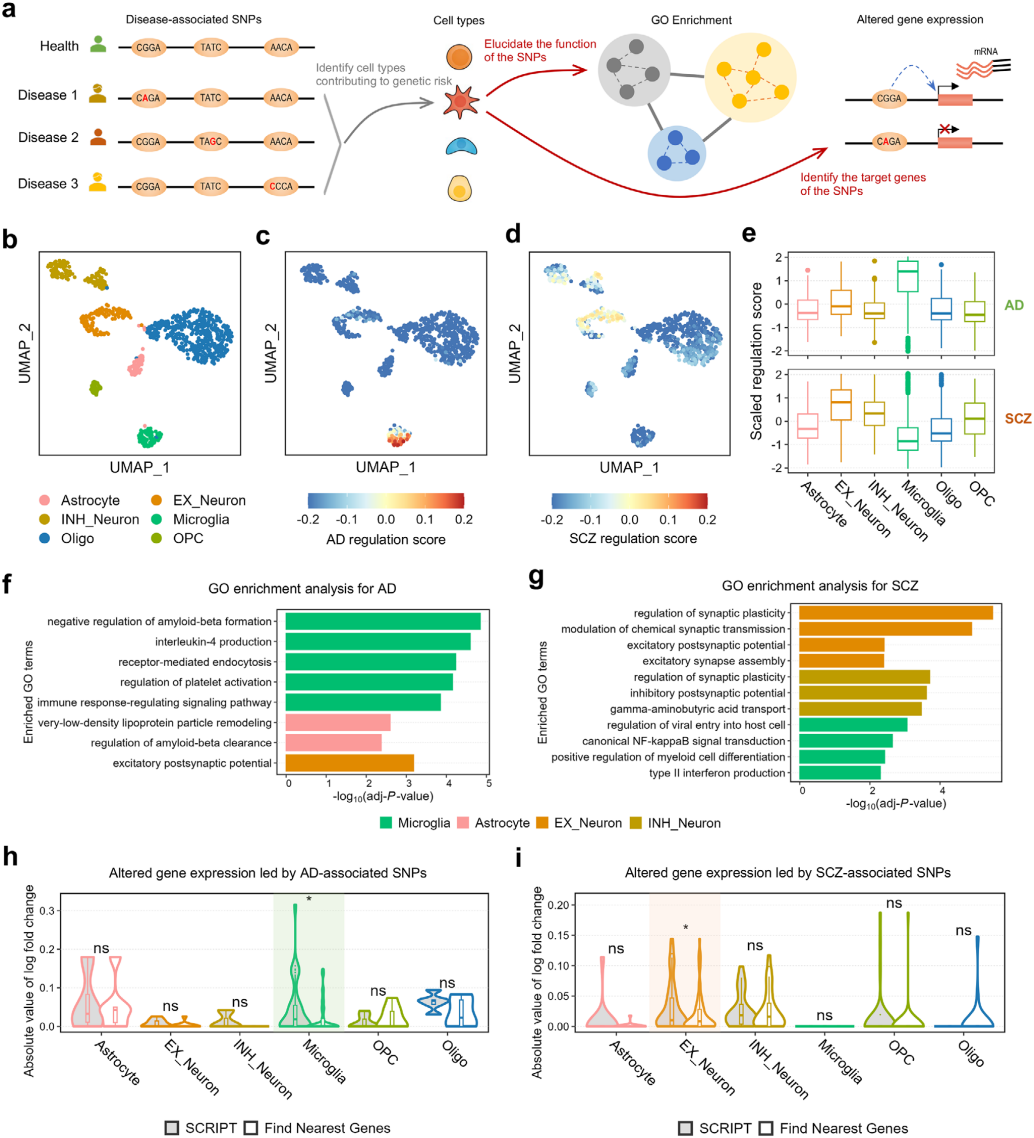
SCRIPT elucidates the pathogenic mechanisms of AD‐ and SCZ‐associated variants. a) Schematic representation of the overall strategy for investigating the pathogenic mechanisms of disease‐associated variants at the cell‐type level using SCRIPT. b) UMAP visualization of scATAC‐seq data from the human cortex dataset including six cell types (*n* = 852 cells). c,d) UMAP visualization of single‐cell mean regulation scores for CRRs related to AD (c) and SCZ (d). e) Boxplots of scaled regulation scores of each human cortex cell type at the CRRs involved in AD‐ and SCZ‐related variants (AD: 141 CRRs; SCZ: 392 CRRs). f,g) Bar plots displaying the enrichments of GO terms in genes regulated by AD‐ and SCZ‐related variants across different cell types. h,i) Violin plots illustrating the absolute values of log fold changes (ALFCs) in gene expression between disease states (AD (h) or SCZ (i)) and controls. Genes identified by SCRIPT and genes determined using the nearest‐gene approach are represented in gray and white, respectively, within the violin plots. Single‐sided Wilcoxon rank sum tests are performed between ALFCs of genes identified by SCRIPT and those of genes determined using the nearest‐gene approach (*P < 0.05). SNP, single nucleotide polymorphism; GO, gene ontology; AD, Alzheimer's disease; SCZ, schizophrenia.

SCRIPT offers a novel approach to explain the pathogenic mechanisms of non‐coding SNPs identified by GWAS. By linking SNPs to their target genes at the single‐cell resolution, SCRIPT identifies cell types contributing to genetic risk from non‐coding SNPs (Tables S6 and S7, Supporting Information). It further elucidates the biological functions influenced by the SNPs and nominates the target genes of the SNPs specific to the cell types of interest (**Figure** **4**a).

The regulation score of each single cell is calculated by averaging the regulation scores of CRRs related to AD or SCZ per cell. Cells labeled as microglia exhibit the highest regulation scores for AD, while excitatory neurons show the highest regulation scores for SCZ (Figure 4b–d). Besides, the regulation score for each CRR was determined by averaging the regulation scores of cells of the same type for CRRs related to AD or SCZ. Consistently, the highest CRR regulation scores were observed in microglia for AD and in excitatory neurons for SCZ (Figure 4e). These results reflect that microglia and excitatory neurons are the primary cell types contributing to genetic risk for AD and SCZ, respectively, which is supported by the literature of AD and SCZ.^[^ ^41^, ^42^ ^]^

Subsequently, we select the genes regulated by the cell‐type‐specific CRRs related to SCZ or AD to perform gene ontology (GO) enrichment analysis. For AD,

Genes specifically regulated in microglia are enriched in gene ontology (GO) terms such as “negative regulation of amyloid‐beta formation”, “receptor‐mediated endocytosis”, and “regulation of platelet activation”, while astrocyte‐specific regulated genes are enriched in “very‐low‐density lipoprotein particle remodeling” and “regulation of amyloid‐beta clearance” (Figure 4f). Extracellular β‐amyloid (Aβ) deposition is a pathological hallmark of AD.^[^ ^43^ ^]^ Activated microglia encase Aβ plaques to form a barrier that limits dissemination and can phagocytose Aβ, thereby attenuating Aβ‐driven neuronal damage. These cellular defense processes have crowned microglia as perhaps the most important cell type in the control of amyloid burden in AD.^[^ ^41^ ^]^ Besides, platelet activation increases plasma Aβ levels, contributing to AD pathogenesis.^[^ ^44^ ^]^ Additionally, Aβ and astrocytes‐derived APOE form an APOE‐Aβ complex which is cleared by a very‐low‐density lipoprotein receptor at the blood‐brain barrier.^[^ ^45^ ^]^

For SCZ, the results of GO enrichment analysis indicate that the excitatory‐neuron‐specific regulated genes are enriched for “regulation of synaptic plasticity”, “modulation of chemical synaptic transmission” and so on. The inhibitory neuron‐specific regulated genes are enriched for “regulation of synaptic plasticity”, “gamma‐aminobutyric acid transport” and so on. Microglia‐specific regulated genes are enriched for “canonical NF‐kappaB signal transduction”, “type II interferon production” and so on (Figure 4g). Synaptic plasticity and neurotransmitter transmission have been suggested to be impaired in SCZ,^[^ ^46^, ^47^ ^]^ and neuroinflammation is considered to contribute to the pathogenesis of SCZ.^[^ ^48^ ^]^ Overall, these pathway enrichment results are consistent with the literature on AD and SCZ.

Finally, scRNA‐seq data for AD and SCZ populations^[^ ^42^, ^49^ ^]^ demonstrate that SCRIPT identifies more biologically relevant target genes for AD‐ and SCZ‐associated SNPs at the cell‐type level. In microglia, the AD‐related genes identified by SCRIPT exhibit significantly greater differential expression than the nearest genes to AD‐associated SNPs (Figure 4h). Similarly, in excitatory neurons, the SCZ‐related genes identified by SCRIPT show significantly greater differential expression than the genes closest to SCZ‐related SNPs (Figure 4i).

### 2.7 SCRIPT Elucidates the Function of Disease‐Related SNPs by Nominating Target Genes

**Figure 5:**
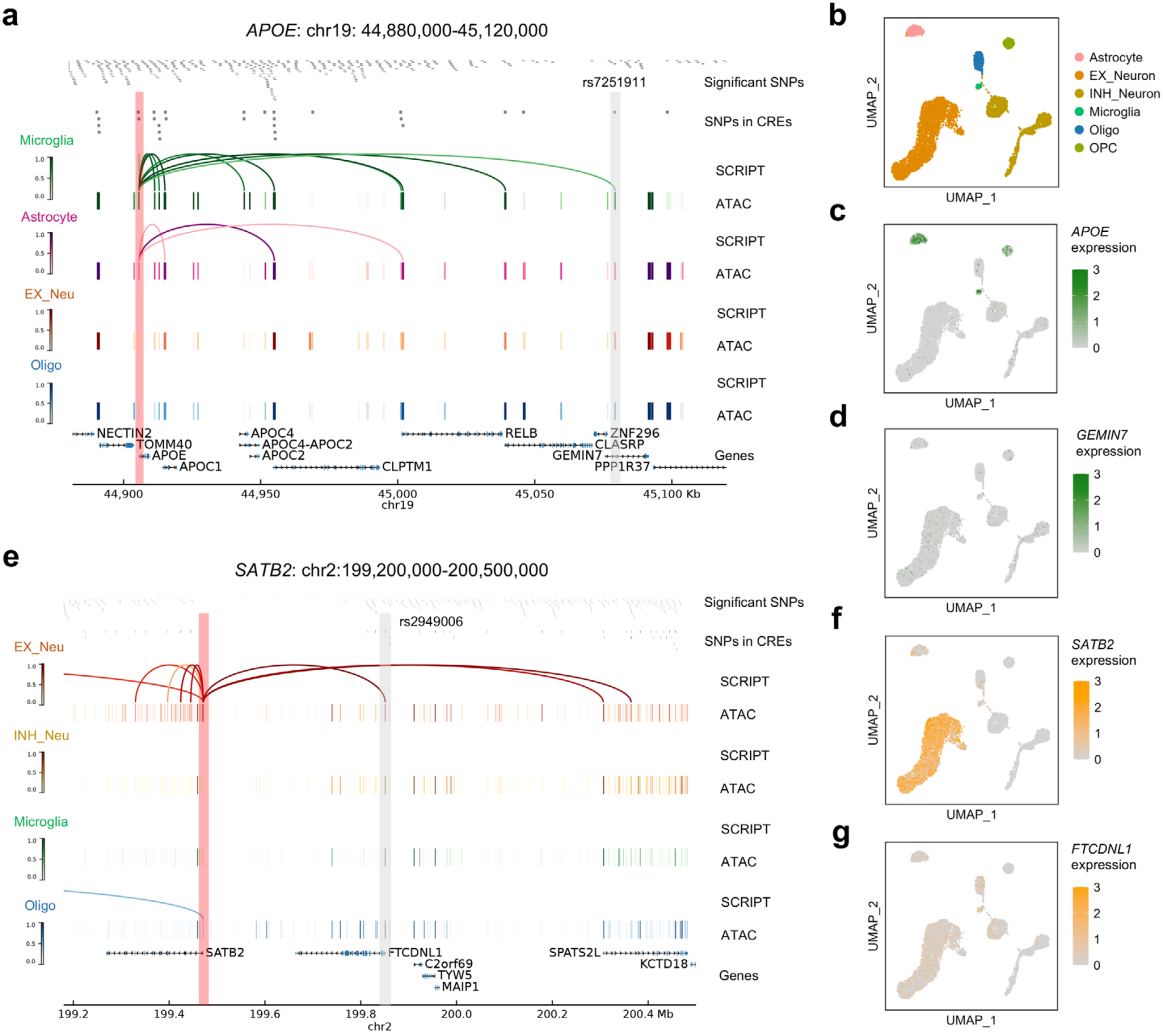
Application of SCRIPT to nominate gene target for AD‐ and SCZ‐related SNPs. a) Genomic visualization of normalized regulation scores predicted by SCRIPT and normalized scATAC‐seq‐derived pseudobulk tracks across four cell types in the *APOE* gene locus (chr19:44880000‐45120000). SNPs significantly associated with AD and located within CREs are displayed at the top of this panel. The genomic locations of SNPs of interest and the *APOE* transcription start site (TSS) are highlighted by gray and red bars, respectively. b) UMAP visualization of scRNA‐seq data from the human cortex dataset including six cell types (*n* = 15594 cells). c,d) UMAP visualization of *APOE* (c) and *GEMIN7* (d) expression across six cortex cell types. e) Genomic visualization of the *SATB2* gene locus (chr2:199200000‐200500000) and SCZ‐related SNPs, following the same organization as Figure 5a. f,g) UMAP visualization of *SATB2* (f) and *FTCDNL1* (g) expression across six cortex cell types.

We present some analytical cases using SCRIPT to elucidate the function of AD‐ or SCZ‐associated SNPs by nominating their target genes. SCRIPT predicts nine microglia‐specific CRRs between *APOE* and nine CREs harboring 19 AD‐associated GWAS SNPs. These CREs are located downstream of the *APOE* TSS, spanning a range from 6 kb to 174 kb. One illustrative example is rs7251911, a SNP located in the intron of *GEMIN7*. SCRIPT predicts that this SNP regulates *APOE*, located 174 kb upstream, through a microglia‐specific CRR (**Figure** **5**a; Figure S21, Supporting Information). *APOE* exhibits high expression levels in microglia and astrocyte (Figure 5b,c). Besides, *APOE* remains the most significant genetic risk factor for AD, implicated in processes such as Aβ peptide aggregation and clearance, tau neurofibrillary degeneration, microglial and astrocytic responses, and blood‐brain barrier disruption—all of which contribute to cognitive decline.^[^ ^45^ ^]^ In contrast, the transcriptional levels of *GEMIN7* are minimal across cortical cell types (Figure 5b,d). In this case, SCRIPT identifies a more plausible mechanism for AD‐causing genetic variants by assigning rs7251911 to *APOE*, rather than *GEMIN7*. Notably, PLAC‐seq data also link rs7251911 to *APOE*, whereas other methods like LINGER and SCARLink fail to identify this long‐range CRR (Figure S22, Supporting Information).

In another analytical case, three CREs containing seven SCZ‐associated GWAS SNPs, located downstream from 379 Kb to 894 Kb, are predicted to regulate *SATB2* through excitatory‐neuron‐specific CRRs. A representative example is rs2949006, an SNP located in the promoter of *FTCDNL1*. This SNP is predicted by SCRIPT to regulate *SATB2* through an excitatory‐neuron‐specific CRR (Figure 5e; Figure S23, Supporting Information). *SATB2* is associated with SCZ risk and is an important transcription factor regulating neocortical organization and circuitry.^[^ ^50^ ^]^ In contrast, *FTCDNL1* exhibits minimal transcriptional levels across cortical cell types (Figure 5f,g). By linking rs2949006 to *SATB2*, SCRIPT identifies SATB2 as a more likely SCZ‐causing gene instead of *FTCDNL1*. PLAC‐seq data of neurons confirm this assignment, while LINGER and SCARLink fail to detect the long‐range CRR (Figure S24, Supporting Information).

Additionally, we employ SCRIPT to identify more cases in *BIN1*, *RIN3*, *MS4A6A*, *MS4A7*, *HCN1*, *MMP16*, *NRGN*, and *SLC32A1* loci (Figures S25–S28, Supporting Information). Previous studies have supported the associations between these genes and AD or SCZ.^[^ ^51^, ^52^, ^53^, ^54^, ^55^, ^56^, ^57^, ^58^ ^]^

These cases demonstrate that SCRIPT provides a more comprehensive prioritization of putative functional SNPs at the cell‐type level compared to traditional approaches, such as LINGER, SCARLink, and nearest‐gene assignment (Figure 1e, middle). By prioritizing functional SNPs and nominating their target genes, SCRIPT enhances our understanding of the functions of non‐coding SNPs. For instance, SNPs located in distinct CREs may regulate the same target gene through long‐range *cis*‐regulation, as predicted by SCRIPT, suggesting potential shared molecular genetic mechanisms. In contrast, traditional methods may overlook these connections, potentially obscuring insights into patient stratification of complex genetic diseases (Figure 1e, right).

## 3 Discussion

SCRIPT is a method that leverages GCAT underpinned by comprehensive empirical CRR evidence, combined with representation learning driven by large‐scale scATAC‐seq data. First, SCRIPT employs GCAT to simulate *cis*‐transcriptional regulation, offering a biologically interpretable alternative to traditional black‐box deep learning models. GCATs selectively update the attention scores of CRE‐gene links supported by empirical CRR evidence. Therefore, the simulation bridges scATAC‐seq and scRNA‐seq data guided by causal biological principles, which prevents SCRIPT from the noises introduced by data correlation. GCAT ensures the reliability of SCRIPT, which not only minimizes false positives but also effectively captures long‐range CRRs. Second, SSGAE enhances CRE representations by effectively capturing the complex interactions within large‐scale scATAC‐seq data. These enhanced representations contribute to improved accuracy in gene expression predictions, further strengthening SCRIPT's ability to identify cell‐type‐specific CRRs. These innovative algorithmic designs ensure that SCRIPT achieves substantially superior CRR inference performance, especially for long‐range CRRs.

More importantly, we present a systematic framework to prioritize candidate disease‐causing SNPs from numerous non‐coding GWAS SNPs at cell type level and elucidate the functions of the disease‐causing SNPs by nominating with high confidence gene and cellular targets for them. Compared to current computational methods, such as LINGER and SCARLink, SCRIPT demonstrates superior accuracy in identifying long‐range CRRs, enabling a more comprehensive annotation of the functional effects of non‐coding SNPs. For example, SCRIPT assigns the AD‐related SNP rs7251911 to *APOE* specific in microglia, and the SCZ‐related SNP rs2949006 to *SATB2* specific in excitatory neurons. In contrast, conventional approaches, which cannot detect long‐range CRRs and rely solely on nearest‐gene assignment in a cell‐type‐agnostic manner, would misattribute these SNPs, limiting their utility for precise genetic‐based patient stratification and novel therapeutic target discovery. More broadly, this study establishes an effective approach for identifying individuals at genetic risk by prioritizing more disease‐causing non‐coding SNPs with high specificity. Furthermore, it provides an avenue toward the nomination of new therapeutic targets that previously remained obscured by the cell heterogeneity and complexity of the regulatory machinery of the noncoding genome.

While SCRIPT offers significant advantages, it also has two limitations that merit further consideration. First, although SCRIPT integrates comprehensive empirical CRR evidence as causal biological principles, and the CRR evidence set covers over 95% of the CRRs in the test datasets, certain rare CRRs may not be captured within this evidence set. As a result, SCRIPT's reliance on the empirical CRR evidence constrains its capacity to predict CRRs beyond current empirical data. In the future, expanding the scope of empirical CRR evidence through the collection of additional experimentally validated datasets, as well as incorporating high‐confidence computational predictions at the bulk level, will help address this limitation. Second, SCRIPT currently employs only scATAC‐seq data for single‐cell CRR prediction because publicly available scATAC‐seq datasets far outnumber those for other single‐cell epigenomic modalities. In the future, as single‐cell epigenomic sequencing technologies continue to advance and datasets for other modalities, such as single‐cell methylation and histone modification data, will become more abundant, we plan to incorporate these additional modalities into SCRIPT to provide deeper insights into gene regulation.

## 4 Experimental Section

### The Inputs for SCRIPT—Input Data

SCRIPT requires two input matrices: a well‐annotated scATAC‐seq count matrix and a corresponding scRNA‐seq count matrix. Notably, the two matrices do not need to originate from single‐cell multi‐omic data where both scRNA‐seq and scATAC‐seq were applied to the same individual cells, but they must be derived from the same tissue. Additionally, SCRIPT relies on a comprehensive empirical CRR evidence database, constructed through the integration of large‐scale bulk Hi‐C and eQTL data.

### Preprocessing

In this study, the reference genome version for the scATAC‐seq matrix is hg38/GRCh38. For datasets based on other reference genome versions, conversion to hg38/GRCh38 can be performed using LiftOver.

Quality control was applied to the scATAC‐seq matrix. By default, cells with fewer captured peaks than the 1st percentile or more than the 99th percentile were excluded. At the gene level, peaks with less than 5% occurrence within any cell type were removed.

Because the low sequencing depth of scATAC‐seq data may hinder CRR predictions, data aggregation was performed. In detail, the scATAC‐seq count matrix was normalized, and 50000 highly variable features were selected. After scaling the data, latent semantic indexing (LSI) was used to reduce the data to 50D. These dimensions were then employed to identify the 10 nearest neighbors for each cell, and the chromatin accessibility profiles of the 11 cells (the target cell and its 10 neighbors) were aggregated.

The scRNA‐seq count matrix was transformed into gene expression profiles of each cell type by aggregating the expression profiles of single cells with the same cell label. For the gene expression profiles, cosg^[^ ^59^ ^]^ was used to identify 300 marker protein‐coding genes for each cell type, generating a marker gene list. Only genes within this list were considered for subsequent modeling. To mitigate the influence of library size, the expression profile matrix was normalized by cell type such that the sum of all gene expression values within each cell type equals 1.

### The Pretraining of Self‐Supervised Graph Autoencoder

During the pretraining stage, the chromatin accessibility matrix derived from scATAC‐seq data is denoted as $X^{P}\in R^{N^{P}\times M^{P}}$, where *N^P^* represents the number of cells and *M^P^* denotes the number of peaks. In the framework, each peak identified from the scATAC‐seq data is considered a CRE. To construct an undirected graph for each cell, the graph of the *n*th cell was defined as $G_{n}^{P}=\left(V_{n}^{P},A_{n}^{P},X_{n}^{P}\right),\;n=1,\;2,\;\text{…},\;N^{P}$, where $V_{n}^{P}$ represents the set of nodes, $M^{P}=\left|V_{n}^{P}\right|$ is the number of nodes, $A_{n}^{P}\in {\left\{0,1\right\}}^{M^{P}\times M^{P}}$ is the adjacent matrix representing CRE‐CRE links, and $X_{n}^{P}\in R^{M^{P}\times 1}$ is the node feature matrix of the *n*th cell. The superscript *P* indicates that these variables were utilized during the pretraining stage.

In the cell graph $G_{n}^{P}$, each node represents a CRE which corresponds a peak in the scATAC‐seq data. The node feature matrix $X_{n}^{P}$ represents the chromatin accessibility levels of the peaks of the *n*th cell. $A_{n}^{P}$ is the adjacent matrix containing CRE‐CRE links derived from three sources: 1) proximal CRE‐CRE links. Two CREs were connected if they were located within the promoter region of the same gene. The promoter region was defined as the genomic interval spanning from 2 kb upstream to 2 kb downstream of the TSS. 2) Hi‐C‐based CRE‐CRE links. If a Hi‐C experiment reports a chromatin interaction between two genomic fragments, all CREs located within the first fragment were linked to all CREs within the second fragment. 3) eQTL‐based CRE‐CRE links. If an eQTL links a gene to a specific SNP, then CREs in the promoter region of the gene were connected to the CRE overlapping the SNP. All genomic overlap operations were performed using bedtools.^[^ ^60^ ^]^

Further, given *f_E_* as the graph encoder, *f_D_* as the graph decoder, and $H_{n}^{P}\in R^{M^{P}\times d}$ denoting the hidden state of the *n*th cell graph encoded by the encoder, the goal of the general self‐supervised graph autoencoder (SSGAE) is to reconstruct the input as

(1) $$H_{n}^{P}=f_{E}\left(X_{n}^{P},A_{n}^{P}\;\right),Z_{n}^{P}=f_{D}\left(H_{n}^{P},A_{n}^{P}\right)$$

where $Z_{n}^{P}$ denotes the reconstructed features.^[^ ^21^ ^]^

In SCRIPT, GAT^[^ ^19^ ^]^ was used as the backbones of *f_E_* and *f_D_* to propagate information between nodes in each cell graph. Given a cell graph, GAT takes as input the hidden state matrix $H^{\left(\mathrm{in}\right)}=\left\{h_{1}^{\left(\mathrm{in}\right)},h_{2}^{\left(\mathrm{in}\right)},\text{…},h_{i}^{\left(\mathrm{in}\right)}\text{…},h_{M}^{\left(\mathrm{in}\right)}\right\}$, where $h_{i}^{\left(\mathrm{in}\right)}$ was the hidden state vector of the *i*th node. The updated hidden state matrix ***H*** ^(***out***)^ is computed as:

(2) $$h_{i}^{\left(\mathrm{out}\right)}=\sum_{j\in N_{i}}e_{ij}\alpha_{ij}W^{\left(1\right)}h_{j}^{\left(\mathrm{in}\right)}$$

(3) $$\alpha_{ij}=\frac{exp\;\left(LeakyReLU\left(a^{T}W^{\left(2\right)}\left[h_{i}^{\left(\mathrm{in}\right)}∥h_{j}^{\left(\mathrm{in}\right)}\right]\right)\right)}{\sum_{k\in N_{i}}exp\;\left(LeakyReLU\left(a^{T}W^{\left(2\right)}\left[h_{i}^{\left(\mathrm{in}\right)}∥h_{k}^{\left(\mathrm{in}\right)}\right]\right)\right)}$$

Here, $N_{i}$ is the set of the neighboring nodes of the *i*th node in the graph $g_{n}^{P}$; *e_ij_* is the weight of the edge linking the *i*th node and the *j*th node; α_*ij*_ is the attention score between the *i*th node and the *j*th node; ***W*** ^(1)^ and ***W*** ^(2)^ are learnable parameter matrices; ***a*** is a learnable parameter vector; ∥ is the concatenation operation.

In the masking stage of SSGAE, for a given cell graph $g_{n}^{P}=\left(V_{n}^{P},X_{n}^{P},A_{n}^{P}\;\right)$, a subset of nodes ${\tilde{V}}_{n}^{P}$ is randomly selected from $V_{n}^{P}$, and their features were replaced with a mask token ***x***_[***M***]_. The masked node feature ${\tilde{x}}_{m,n}^{P}$ for $v_{m,n}\in {\tilde{V}}_{n}^{P}$ in the masked feature matrix ${\tilde{X}}_{n}^{P}$ is defined as:

(4) $${\tilde{x}}_{m,n}^{P}=\left\{\begin{array}{c} x_{\left[M\right]},\;v_{m,n}\in {\tilde{V}}_{n}^{P}\\ x_{m,n}^{P},\;v_{m,n}\notin {\tilde{V}}_{n}^{P} \end{array}\right.$$

The objective of SSGAE is to reconstruct the masked node features in ${\tilde{V}}_{n}^{P}$ given the partially observed node signals ${\tilde{X}}_{n}^{P}$ and the input adjacency matrix $A_{n}^{P}$.

In the reconstruction stage of SSGAE, scaled cosine error (SCE) is leveraged as the loss function to reconstruct original node features. Given the original feature $X_{n}^{P}$ and reconstructed output $Z_{n}^{P}$, the SCE for SSGAE is defined as:

(5)

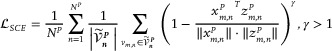

where $x_{m,n}^{P}$ and $z_{m,n}^{P}$ respectively represent the components of $X_{n}^{P}$ and $Z_{n}^{P}$ in the *m*‐th node *v*_*m*,*n*_ of *n*‐th cell, and the easy samples’ contribution is down‐weighted during training by scaling the cosine error with a power of γ > 1. Here, easy samples were defined as nodes exhibiting low cosine error during reconstruction. For predictions with high confidence, their corresponding cosine errors were usually smaller than 1 and decay faster to zero when the scaling factor γ >.1^[^ ^21^ ^]^

### *Cis*‐Regulation Simulation using Graph Causal Attention Networks

For a specific tissue, a scATAC‐seq dataset is used as input, and the matched gene expression profile matrix corresponding to each cell in the scATAC‐seq dataset is used as the label to train the *cis*‐regulation simulation model. From the scATAC‐seq data, a cell‐by‐peak matrix $X^{A,F}\in R^{N^{F}\times M^{A}}$, was constructed where *N^F^* is the number of cells and *M^A^* is the number of peaks, that is, the number of CREs. In parallel, a gene feature matrix $X^{R,F}\in R^{N^{F}\times M^{R}}$ was generated where *M* ^***R***^ denotes the number of genes. Each feature in $X^{R,F}$ is initialized as the chromatin accessibility value of the CRE nearest to the TSS of the corresponding gene. The superscript *F* indicates that these variables were utilized during the *cis*‐regulation simulation stage.

For the *n*th cell, the peak and gene features were concatenated into a joint matrix $X_{n}^{F}\in R^{N^{F}\times \left(M^{A}+M^{R}\right)}$, and construct a directed graph $G_{n}^{F}=\left(V_{n}^{F},A_{n}^{F}\cdot M_{n}^{F},X_{n}^{F}\right),\;n=1,\;2,\;\text{…},\;N$, where $V_{n}^{F}$ represents the set of nodes, $|V_{n}^{F}|=M^{A}+M^{R}=M^{F}$ is the number of nodes, $A_{n}^{F}\in {\left\{0,1\right\}}^{M^{F}\times M^{F}}$ represents the adjacency matrix encoding CRE‐gene links, and $M_{n}^{F}\in {\left\{0,1\right\}}^{M^{F}\times M^{F}}$ represents the causal attention mask matrix^[^ ^20^ ^]^ that guarantees directionality within the graph. Each element 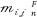 in the *i‐*th row and *j*‐th column of $M_{n}^{F}$ is assigned based on the following rule:

(6)

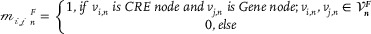




In the cell graph $G_{n}^{F}$, each node represents either a CRE (corresponding to a peak of the *n*th cell in $X^{A,F}$), or a gene corresponding a row in $X^{R,F}$. The node feature matrix $X_{n}^{F}$ encodes the chromatin accessibility values of both CRE and gene nodes for the *n*th cell. The adjacent matrix $A_{n}^{F}$ encodes CRE‐gene links derived from three sources: 1) Proximal CRE‐gene links. A CRE was linked to a gene if it was located within the gene's promoter region, defined as ±2 kb around the TSS. 2) Hi‐C‐based CRE‐gene links. If a Hi‐C experiment indicates that two genomic fragments were spatially proximal, all CREs in the first fragment were linked to all genes in the second fragment. 3) eQTL‐based CRE‐gene links. If a gene is associated with a SNP through an eQTL, it is linked to the CRE overlapping that SNP. All genomic intersection operations were implemented by bedtools.^[^ ^60^ ^]^

For the *n*th cell graph $G_{n}^{F}$, a linear layer followed by a RELU activation function encodes $X_{n}^{F}$ into the hidden state matrix $H_{1}^{F,1}\in R^{M^{F}\times d}$, while SSGAE to encode $X_{n}^{F}$ to the hidden state matrix $H_{2}^{F,1}\in R^{M^{F}\times d}$. The GCAT layer φ is employed to obtain the updated node hidden state matrix ***H*** ^***F***,2^ as follows:

(7) $$H^{F,2}=\varphi \left(H_{1}^{F,1}∥H_{2}^{F,1},A_{n}^{F}\cdot M_{n}^{F}\right)$$

Here, ∥ represents the concatenation operation.

Then, ***H*** ^***F***,2^ is pooled into a vector $u_{n}^{F}$ with the same size as $X_{n}^{F}$. $u_{n}^{\left(\mathrm{gene}\right)}$ is obtained by selecting the gene nodes from $u_{n}^{F}$. Then, $u_{n}^{\left(\mathrm{gene}\right)}$ is normalized as follows:

(8) $$u_{n}^{\left(\mathrm{norm}\right)}=\frac{u_{n}^{\left(\mathrm{gene}\right)}-E\left[u_{n}^{\left(\mathrm{gene}\right)}\right]}{\sqrt{Var\left[u_{n}^{\left(\mathrm{gene}\right)}\right]+\varepsilon }}$$

where *E*[·] is the calculation of mathematical expectation, *Var*[·] is the calculation of variance, ε is a value added to the denominator for numerical stability. Here, $u_{n}^{\left(\mathrm{norm}\right)}=\left\{u_{\left(n,1\right)}^{\left(norm\right)},u_{\left(n,2\right)}^{\left(norm\right)},\text{…},u_{\left(n,g\right)}^{\left(norm\right)},\text{…},u_{\left(n,G\right)}^{\left(norm\right)}\right\}$ contains *G* values, and $u_{\left(n,g\right)}^{\left(norm\right)}$ is the normalized predicted value of the *g*th gene in the *n*th cell.

Subsequently, a softmax function is used to transform each element of $u_{n}^{\left(\mathrm{norm}\right)}$ into a probability as follows:

(9) $$Softmax\left(u_{\left(n,g\right)}^{\left(norm\right)}\right)=\frac{exp\;\left(u_{\left(n,g\right)}^{\left(norm\right)}\right)}{\sum_{m=1}^{M}exp\left(u_{\left(n,g\right)}^{\left(norm\right)}\right)}$$

This transformation yields $u_{n}^{\left(\mathrm{pred}\right)}$, representing the predicted probability vector of sequence reads in each gene region. The true probability vector of the *n*th cell, denoted as $u_{n}^{\left(\mathrm{true}\right)}$, is derived from the gene expression profile of the cell type to which the nth cell belongs. The first part of the loss function of the *cis*‐regulation simulation model, termed the gene expression loss function, is calculated as follows:

(10) $$L_{1}=KL\left(q\left(u_{n}^{\left(\mathrm{pred}\right)}|g_{n}^{F},\Theta \right)∥p\left(u_{n}^{\left(\mathrm{true}\right)}\right)\right)$$

where *KL*(*q*(·)∥*p*(·)) is the Kullback‐Leibler divergence between predicted distribution $q(u_{n}^{\left(\mathrm{pred}\right)}|g_{n}^{F},\Theta )$ and prior distribution $p(u_{n}^{\left(\mathrm{true}\right)})$, and Θ is the parameter of the *cis*‐regulation simulation model.

Additionally, $u_{n}^{\left(\mathrm{norm}\right)}$ serves as input to a multilayer perceptron to generate the label probability vector of the *n‐*th cell ***y***_***n***_ = $\left\{y_{\left(n,1\right)},y_{\left(n,2\right)},\text{…}y_{\left(n,c\right)},\text{…}y_{\left(n,C\right)}\right\}$, where the model predicts one of seven possible labels and *y*_(*n*,*c*)_ represents the probability that the *n*th cell belongs to the *c‐*th label. The true cell‐type label vector is denoted as $C_{n}=\left\{C_{\left(n,1\right)},C_{\left(n,2\right)},\text{…}C_{\left(n,c\right)},\text{…}C_{\left(n,C\right)}\right\}$, where $C_{\left(n,c\right)}$ is 1 if the *n‐*th cell belongs to class *c*, and 0 otherwise. The second part of the loss function of the *cis*‐regulation simulation model, the label loss function, is defined as the weighted cross‐entropy between the predicted and true label vector:

(11) $$L_{2}=-\frac{1}{N}\sum_{n=1}^{N}\sum_{c=1}^{C}\frac{1}{N_{c}}C_{\left(n,c\right)}\log\;\left(y_{\left(n,c\right)}\right)$$

where $\frac{1}{N_{c}}$ is the weight of the *c‐*th label, *N_c_* is the number of the *c‐*th label in the training dataset.

Finally, the total loss function is defined as:

(12) $$L=L_{1}+\lambda L_{2}$$

where λ balances the contributions of *L*_1_ and *L*_2_ in model optimization and is set to 1 by default.

### The Regulation Score Prediction based on the Attribution Method

In the test dataset, the well‐trained *cis*‐regulation simulation model was used to predict the gene expression of each cell. To infer single‐cell regulation scores, an attribution method, integrated gradients (IG),^[^ ^61^ ^]^ was applied, in which the influence of changes in edge weights in each cell graph on the expression levels of regulated genes was quantified.

For the *g*th gene in the *n*th cell, $e_{ij}^{\left(cre\right)}$ represents an edge weight in the CRR networks $e^{\left(\mathrm{cre}\right)}$. The influence of a change of $e_{ij}^{\left(cre\right)}$ on the expression level of the *g*th gene is calculated as:

(13)

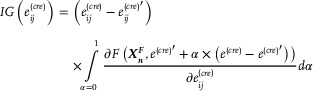

where *F*(·) denotes the predicted expression level of the *g*th gene, and $\frac{\partial F(v_{n},{e^{\left(cre\right)}}^{\prime }+\alpha \times \left(e^{\left(cre\right)}-{e^{\left(cre\right)}}^{\prime }\right))}{\partial e_{ij}^{\left(cre\right)}}$ is the gradient of *F*(·) with respect to $e_{ij}^{\left(cre\right)}$, computed when the node feature vector is $X_{n}^{F}$ and the edge weights of the cell graph is ${e^{\left(cre\right)}}^{\prime }+\alpha \times \left(e^{\left(cre\right)}-{e^{\left(cre\right)}}^{\prime }\right)$. By default, all elements of *e* ^(*cre*)^ were set as 1 and set all elements of baseline ${e^{\left(cre\right)}}^{\prime }$ as 0. The calculation of attribution scores can be simplified as:

(14) $$IG\left(e_{ij}^{\left(cre\right)}\right)=\int_{\alpha =0}^{1}\frac{\partial F\left(v_{n},\alpha \right)}{\partial e_{ij}^{\left(cre\right)}}d\alpha$$

Using this equation, the attribution scores was computed for all edges connected to the *g*th gene in the *n*‐th cell. This process was repeated for each gene in the *n*‐th cell, yielding an attribution score vector with a length equal to the number of edges in the cell graph.

Finally, the regulation score (RS) of each CRR per cell was computed by normalizing the corresponding attribution score to a range between −1 and 1, using the following transformation:

(15) $$RS\left(e_{ij}^{\left(cre\right)}\right)=\tanh\left(\frac{IG\left(e_{ij}^{\left(cre\right)}\right)}{SD\left(IG\right)}\right)$$

where tanh was a hyperbolic tangent function, and *SD*(*IG*) is the standard deviation of all attribution scores.

### The Modified Version of SCRIPT

To investigate the effectiveness of pretraining on atlas‐scale single‐cell data in CRR prediction, two modified versions of SCRIPT were developed: SCRIPT pretrained on a small dataset (SCRIPT‐POSD) and SCRIPT without pretraining (SCRIPT‐WOP). In SCRIPT‐WOP, the embeddings generated by the SSGAE component were not used as inputs during the cis‐regulation simulation stage. In SCRIPT‐POSD, instead of leveraging the full atlas‐scale dataset, the SSGAE component of SCRIPT was pretrained using only the scATAC‐seq data from the *cis*‐regulation simulation stage, which involved a substantially smaller number of cells.

### Data Collections

All datasets used in this study were downloaded from public databases.

The construction of the empirical CRR evidence database relies on large‐scale pcHi‐C and eQTL datasets, sourced as follows:

- The large‐scale pcHi‐C dataset was obtained from the study by Inkyung Jung et al,^[^ ^4^ ^]^ with accession number GSE86189 in the GEO database. In the study, the pcHi‐C technique was used to obtain chromatin interaction data in the 27 human cell/tissue types. Preprocessed chromatin interaction files were downloaded from http://3div.kr/capture_hic. As the raw read pairs were mapped to the human genome hg19, the genomic coordinates was converted into the hg38 reference using the UCSC LiftOver tool.
- The *cis*‐eQTL dataset, derived from 49 human tissues, was provided by the GTEx project^[^ ^10^ ^]^ and was available at https://www.gtexportal.org/.

The human scATAC‐seq atlas from the study by Kai Zhang et al^[^ ^62^ ^]^ was utilized to pretrain the SSGAE, and its accession number in the GEO database is GSE184462.

Several scRNA‐seq and scATAC‐seq datasets were used to predict single‐cell CRRs, and their sources are as follows:

- The scRNA‐seq dataset of the middle temporal gyrus of the human cortex was obtained from the study by Rebecca D. Hodge et al^[^ ^63^ ^]^ and is available at http://celltypes.brain‐map.org/.
- The scATAC‐seq dataset of the human cortex was obtained from the study by Ryan M. Mulqueen et al^[^ ^40^ ^]^ (GEO accession number: GSE174226).
- The scRNA‐seq and scATAC‐seq datasets of the human prefrontal cortex were from the study by Samuel Morabito et al,^[^ ^64^ ^]^ with accession number GSE174367 in the GEO database.
- The scRNA‐seq and scATAC‐seq datasets of human peripheral blood mononuclear cells (PBMC) were sourced from 10X Genomics website and were downloaded from https://support.10xgenomics.com/single‐cell‐gene‐expression/datasets.
- The scRNA‐seq and scATAC‐seq datasets of human bone marrow mononuclear cells (BMMC) and PBMC were derived from the study by Jeffrey M. Granja et al,^[^ ^65^ ^]^ and their accession number in the GEO database is GSE139369.
- The scRNA‐seq and scATAC‐seq datasets of human cancer cell lines were derived from the study by Qionghua Zhu et al.^[^ ^65^ ^]^ The processed scRNA‐seq and scATAC‐seq data generated in this study were available in CNSA with accession number CNP0003658.

To validate the predicted cell‐type‐specific CRRs, cell‐type‐specific chromatin contact datasets, cell‐type‐specific CRISPR perturbation datasets, and cell‐type‐specific ChIP‐seq datasets were utilized, sourced as follows:

- The PLAC‐seq and H3K27ac ChIP‐seq data for three human cortex cell types were obtained from the study by Alexi Nott et al.^[^ ^24^ ^]^ The chromatin interaction data from PLAC‐seq and the bed files from H3K27ac ChIP‐seq were available in supplementary Table S5 (Supporting Information) of their paper. All genomic coordinates were originally mapped to the hg19 reference genome and were subsequently converted to hg38 using the UCSC LiftOver tool.
- The pcHi‐C data for 17 human blood cell types were obtained from the study by Biola M. Javierre et al.^[^ ^25^ ^]^ The chromatin interaction files were provided in supplementary Table S1 (Supporting Information) of their paper. As with the previous dataset, genomic coordinates originally based on hg19 were converted to hg38 using the UCSC LiftOver tool.
- The H3K27ac ChIP‐seq data of four immune cell types were obtained from the Roadmap Epigenomics Project^[^ ^31^ ^]^ and were available at https://egg2.wustl.edu/roadmap/web_portal/processed_data.html. The bed files aligned to the hg38 reference genome were downloaded directly from the ENCODE Project.
- The BACH2 ChIP‐seq data of B cells were obtained from the study by Srividya Swaminathan et al^[^ ^34^ ^]^ (GEO accession number: GSE44420). The bed files aligned to the hg38 reference genome were downloaded directly from the Cistrome database.^[^ ^66^ ^]^
- The CRISPRi data for K562 cell line were obtained from the study by Fulco et al.^[^ ^26^ ^]^ and Gasperini et al.,^[^ ^27^ ^]^ respectively. The processed enhancer‐gene link files were provided in Supporting tables of these two studies. As with the previous dataset, genomic coordinates originally based on hg19 were converted to hg38 using the UCSC LiftOver tool.
- The H3K27ac ChIP‐seq data for K562 cell line were obtained from the study by Zhang.^[^ ^37^ ^]^ The bed file aligned to the hg38 reference genome were downloaded directly from the ENCODE Project.

Two GWAS summary statistics datasets were employed to explore the pathogenic mechanisms of AD and SCZ, with sources as follows:

- GWAS summary statistics data for AD were obtained from a meta‐analysis by E. Jansen et al.^[^ ^38^ ^]^ (71880 cases, 383378 controls) and were available at https://ctg.cncr.nl/software/summary_statistics.
- GWAS summary statistics data of SCZ were obtained from meta‐analysis by Antonio F. Pardiñas et al.^[^ ^39^ ^]^ (40675 cases, 64643 controls) and were available at https://ctg.cncr.nl/software/summary_statistics.

The scRNA‐seq data of AD and SCZ populations were sourced from the studies by Hansruedi Mathys et al.^[^ ^49^ ^]^ and W. Brad Ruzicka et al.,^[^ ^42^ ^]^ respectively. Differential expression gene lists across different cell types for AD and SCZ were extracted from Supporting tables of the two studies.

### Implementation Details

For SSGAE, the encoder consists of two GAT layers, while the decoder contains one GAT layer. The *cis*‐regulation simulation model was implemented with a single GAT layer. In all GAT layers used in this study, the node hidden state has a dimension of 64, the number of multi‐head attentions was set to 4, and the dropout probability for the normalized attention coefficients—allowing each node to be exposed to a stochastically sampled neighborhood during training—is set to 0.5. The masking ratio for SSGAE was also set to 0.5.

Model parameters were optimized using the Adam algorithm, with initial learning rates of 1  × 10^−4^ for SSGAE and 5  × 10^−4^ for the *cis*‐regulation simulation model. To prevent overfitting, L2 regularization was applied with a weight decay parameter of 1  × 10^−4^.

During the *cis*‐regulation simulation, for each scATAC‐seq dataset, 50% of the cells were randomly selected as the training set and 10% as the validation set to train the gene expression model. The well‐trained model was then used to predict the gene expression matrix for the remaining 40% of test cells. Finally, the attribution model was applied to the test set to obtain regulatory scores.

### Evaluation for Cell‐Type‐Specific CRR Prediction

To assess the accuracy of cell‐type‐specific CRR predictions, two metrics were employed: cell‐level AUC and reg‐level AUC.

AUC quantifies a classifier's ability to distinguish between two classes. It was computed by plotting the true positive rate (TPR) against the false positive rate (FPR) across different thresholds. The AUC value represents the probability that a randomly selected positive example was ranked higher than a randomly selected negative example. A perfect classifier has an AUC of 1, while a random classifier has an AUC of 0.5.

To evaluate the accuracy of each method under class imbalance, the AUPR ratio was adopted as a performance metric, following established practices in previous studies.^[^ ^17^ ^]^ The AUPR ratio was a normalized metric based on AUPR, which was defined as the ratio between the AUPR achieved by a given method and the AUPR of a random predictor. Unlike AUROC, which has a fixed baseline of 0.5 regardless of dataset composition, AUPR lacks a constant baseline, as its value varies with the class distribution of the evaluation dataset. Consequently, raw AUPR values may be confounded by dataset‐specific characteristics and do not always reflect the true performance difference between models. In contrast, the AUPR ratio allows for fairer comparisons between methods by focusing on relative improvements over the random predictor rather than absolute values that may be dataset‐dependent. By normalizing against a dataset‐specific random baseline, the AUPR ratio effectively isolates performance differences attributable to the model itself, making it as a more discriminative metric. The experimental results confirm that both AUPR and AUPR ratio consistently support the superior performance of SCRIPT. However, the AUPR ratio exhibits greater discriminative power than AUPR when comparing the performance of different methods (Figure S29, Supporting Information). Notably, an AUPR ratio greater than 1 indicates performance above random, and higher values reflect stronger predictive power.

The result of CRR prediction was a regulation score matrix, where rows correspond to cell types and columns to CRRs. The cell‐level AUC evaluates whether the regulation scores of a given cell type can effectively distinguish cell‐type‐specific CRRs from non‐specific CRRs. The reg‐level AUC assesses whether the regulation scores of an experimentally validated cell‐type‐specific CRR can distinguish the corresponding cell type from others. Notably, since the majority of experimentally supported enhancers lie within 1 Mb of their target gene's TSS,^[^ ^4^ ^]^ the AUC metrics were calculated using only those CRRs located within this range.

For comparisons between predicted CRRs and cell‐type‐specific chromatin contact data, cell subtypes were merged to ensure consistency in cell‐type annotations. In brain tissue, excitatory and inhibitory neurons were combined into a single neuron category. In the PBMC tissue, subtypes was consolidated as follows: CD4^+^ naïve T cells, CD4^+^ central memory T cells, CD4^+^ effector memory T cells, and regulatory T cells were grouped into CD4^+^ T cells; CD8^+^ naïve T cells and CD8^+^ effector memory T cells were merged into CD8^+^ T cells; CD14^+^ monocytes and CD16^+^ monocytes were combined into monocytes; and naïve B cells, intermediate B cells, and memory B cells were categorized as B cells.

### Identification of Marker CRRs and Marker Genes

Marker CRRs for each cell type as follows. For each CRR in the single‐cell regulation score matrix, the intra‐group distances of regulation scores were first computed within a given cell type, followed by the inter‐group distances of regulation scores between this cell type and all others. The fold change (FC) value was defined as the mean inter‐group distances divided by the mean intra‐group distance. To assess statistical significance, the Wilcoxon rank‐sum test was applied to determine whether the inter‐group distances were significantly greater than the intra‐group distances. A CRR was deemed specific to a cell type if the FC value exceeds 1.5 and the Bonferroni‐corrected *P*‐value is below 0.05.

The FindAllMarkers function was performed of the Seurat package^[^ ^67^ ^]^ to identify marker genes of each cell type from a single‐cell gene expression matrix. Only the genes with FC value >1.5 and Bonferroni‐corrected *P*‐value < 0.05 were regarded as marker genes.

### Visualization of CRR Prediction

For the predicted single cell regulation score matrix, 3000 highly variable CRRs were selected, and PCA is performed to reduce this matrix to 50 dimensions. UMAP was then applied for the 50 PCs to visualize the single‐cell regulation score matrix to compare the performance of three methods in capturing biologically relevant signals related to cell type.

To visualize CRRs and scATAC‐seq peaks in specific genomic regions, pyGenomeTracks was used.^[^ ^68^ ^]^ Only predicted CRRs with regulation scores greater than 0.05 were displayed. For each visualized gene locus, the regulation scores generated by each computational method were normalized by dividing by their respective maximum values. For the cell‐peak matrix derived from scATAC‐seq data, pseudobulk scATAC‐seq profiles were generated by aggregating single cells belonging to the same cell type. The pseudobulk scATAC‐seq data were then normalized using a size factor of 10^5^. Since most normalized accessibility scores fall within the range of 0 to 1, values exceeding 1 were clipped to 1.

### Transcription Factor Analysis

To assess the potential of an enhancer to bind specific TFs, the corresponding DNA sequence was first extracted from the hg38 reference genome using bedtools. Position weight matrices (PWMs) from the JASPAR database were then used to predict transcription factor binding sites (TFBSs) within the enhancer sequence.^[^ ^69^ ^]^ TFBS predictions with a PWM relative score ≥ 0.8 and a p‐value < 0.05 were retained for downstream analysis, following the recommended thresholds provided by JASPAR.

### Application of SCRIPT to Explain Disease‐Associated Variants

Variants with *P* values less than 1  × 10^−5^ were defined in GWAS as disease‐associated SNPs. To analyze these variants, scATAC‐seq and scRNA‐sq data were selected from disease‐relevant tissues and applied to SCRIPT to generate a single‐cell regulation score matrix for the tissue. Using bedtools, disease‐associated SNPs were mapped to CREs identified in the scATAC‐seq data. CRRs involving these disease‐associated CREs were then extracted to construct a disease‐associated single‐cell regulation score matrix. The regulation score vector for each cell type was obtained by averaging the regulation score vectors of all cells belonging to that cell type. To facilitate comparison across CRRs, a Z‐transformation was performed on the regulation scores of all cell types for each CRR, yielding scaled regulation scores.

To elucidate the biological functions of disease‐associated variants, genes regulated by cell‐type‐specific CRRs were identified, and GO enrichment analysis was conducted. GO enrichment analysis was performed using ClusterProfiler,^[^ ^70^ ^]^ with multiple testing corrections applied via the Benjamini‐Hochberg method at a significance threshold of 0.05.

To evaluate whether the target genes identified by SCRIPT were more biologically relevant to AD or SCZ, scRNA‐seq data from AD and SCZ cohorts obtained from studies conducted by Hansruedi Mathys et al.^[^ ^49^ ^]^ and W. Brad Ruzicka et al.,^[^ ^42^ ^]^ respectively, were analyzed. These studies provide the results from differential gene expression analysis (DGEA) for each cell type, comparing control samples with either AD or SCZ samples. For disease‐associated variants of each disease, two target gene lists were generated: one using SCRIPT and the other by identifying the nearest gene to each variant. The absolute values of log fold changes for both gene lists were extracted from the DGEA results. To determine whether genes identified by SCRIPT exhibit significantly higher absolute log fold changes compared to those from the nearest‐gene approach, the Wilcoxon rank‐sum test was applied. Notably, the absolute log fold change of a gene was set to 0 if its false discovery rate (FDR) exceeds 0.05.

### Competing Methods

In this study, SCRIPT was compared against two existing methods, SCARLink and LINGER, which were dowloaded from https://github.com/snehamitra/SCARlink and https://github.com/Durenlab/LINGER, respectively. LINGER and SCARLink employ neural networks and Poisson regression models to predict gene expression from chromatin accessibility, respectively, and subsequently infer single‐cell CRRs via Shapley value analysis.

By default, LINGER considers candidate CREs within ±1 Mb of the TSS when inferring CRRs, while SCARLink restricts its analysis to a ±250 kb window around each gene body. Given that the majority of experimentally validated enhancer‐target gene interactions were located within 1 Mb of the TSS and that the evaluation was limited to this range, the genomic window was extended for SCARLink to ±1 Mb. This adjustment ensures consistency in evaluation criteria and enables a fair comparison across methods.

To examine whether incorporating CRR evidence improves the prediction performance of LINGER and SCARLink, both methods were modified by replacing their original distance‐based candidate CREs with the CRR evidence‐based CREs used by SCRIPT for each gene. These modified versions, referred to as LINGER‐SCE (LINGER supported by CRR evidence) and SCARLink‐SCE (SCARLink supported by CRR evidence).

### Run Time Analysis

SSGAE was trained on eight NVIDIA A100 GPUs (batch size of 8, with 1 per core) for 812500 steps, requiring a total of 156.4 h. While SCRIPT requires substantial GPU resources during the pretraining phase, this process only needs to be conducted once. For downstream single‐cell CRR prediction tasks, users can directly load the pretrained model parameters without the need for retraining was provided. For single‐cell CRR prediction on the cortex dataset, SCRIPT required 5.3 h using a single NVIDIA A100 GPU with a batch size of 16. The runtime and memory footprint of SCRIPT, SCARLink, and LINGER across the five benchmark datasets are summarized in Figure S30 (Supporting Information).

### Statistical Analysis

All statistical analyses were performed using R software (version 4.3.2), including the single‐sided Wilcoxon rank‐sum test, Fisher's exact test, and multiple testing correction. *P* < 0.05 was considered statistically significant. All boxplots display the median (center line), the 25^th^ and 75^th^ percentiles (bounds of the box), and the minimum and maximum values (whiskers). The sample sizes used for plots, as well as the P values and statistical tests employed, were provided in the corresponding figure legends.

## Conflict of Interest

The authors declare no conflict of interest.

## Author Contributions

Y.Z., B.W., Y.J. and Y.L. contributed equally to this work. Y.Z., W.T., Y.H., Y.C., Y.Q., Y.X., X.G., and L.H. conceived this study. Y.Z., Y.J., Y.L., X.G., Y.X., Y.C., W.T., and Y.H. designed the computational framework of SCRIPT. Y.Z., Y.J., and Y.L. carried out the implementation of SCRIPT. Y.Z., B.W., Y.J., and Y.L. performed the benchmark analyses. Y.Z. and Y.H. performed the bioinformatic analyses to elucidate pathogenic mechanisms. Y.Z., B.W., X.G., Y.W., J.L., X.G., Y.H. and W.T. wrote the manuscript with all the authors’ inputs. Y.Q., Y.C., Y.H. and W.T. supervised the project. All authors read and approved the final manuscript.

## Code Availability

SCRIPT was available on GitHub (https://github.com/Drizzle‐Zhang/SCRIPT), together with usage documentation.

## Supporting information



Supporting Information

Supporting Information

## Data Availability

All datasets in this study were obtained from their public accessions. The detailed information including the data download and publication citations for all datasets can be seen in Materials and Methods.
