# Supplementary material for: SCRIPT: Predicting Single‐Cell Long‐Range Cis‐Regulation Based on Pretrained Graph Attention Networks
Source: Adv Sci (Weinh). 2025 Aug 20;12(41):e05021. doi: 10.1002/advs.202505021 (PMC12591183; doi:10.1002/advs.202505021)
Supplement: Supplementary file 1 — Supporting Information [file ADVS-12-e05021-s001.pdf]

Supporting Information

SCRIPT: Predicting Single-cell Long-range Cis-regulation Based on Pretrained Graph Attention Networks

Yu Zhang[1,2,3†], Baole Wen[4†], Yifeng Jiao[2†], Yuchen Liu[1,2†], Xin Guo[2], Yushuai Wu[2], Jiyang Li[2], Limei Han[1,2], Yinghui Xu[1,3], Xin Gao[2,5,6,7], Yuan Qi[1,2,8], Yuan Cheng[1,2*], Ying He[2*], Weidong Tian[4,9,10*]*

Affiliations

1. Artificial Intelligence Innovation and Incubation Institute, Fudan University, Shanghai, China
2. Shanghai Academy of Artificial Intelligence for Science, Shanghai, China
3. INF Technology (Shanghai) Co. Ltd, Shanghai, China
4. State Key Laboratory of Genetics and Development of Complex Phenotypes, Department of Computational Biology, School of Life Sciences, Fudan University, Shanghai, China.
5. Computer, Electrical and Mathematical Sciences and Engineering Division, King Abdullah University of Science and Technology (KAUST), Thuwal, Saudi Arabia
6. Center of Excellence for Smart Health, King Abdullah University of Science and Technology (KAUST); Thuwal, Saudi Arabia
7. Center of Excellence on GenAI, King Abdullah University of Science and Technology (KAUST); Thuwal, Saudi Arabia
8. Zhongshan Hospital, Fudan University, Shanghai, China
9. Children's Hospital of Fudan University, Shanghai, China
10. Children's Hospital of Shandong University, Jinan, China

† These authors contributed equally to this work.

*Corresponding authors. Emails: qiyuan@fudan.edu.cn, cheng_yuan@fudan.edu.cn, heying340@gmail.com, weidong.tian@fudan.edu.cn

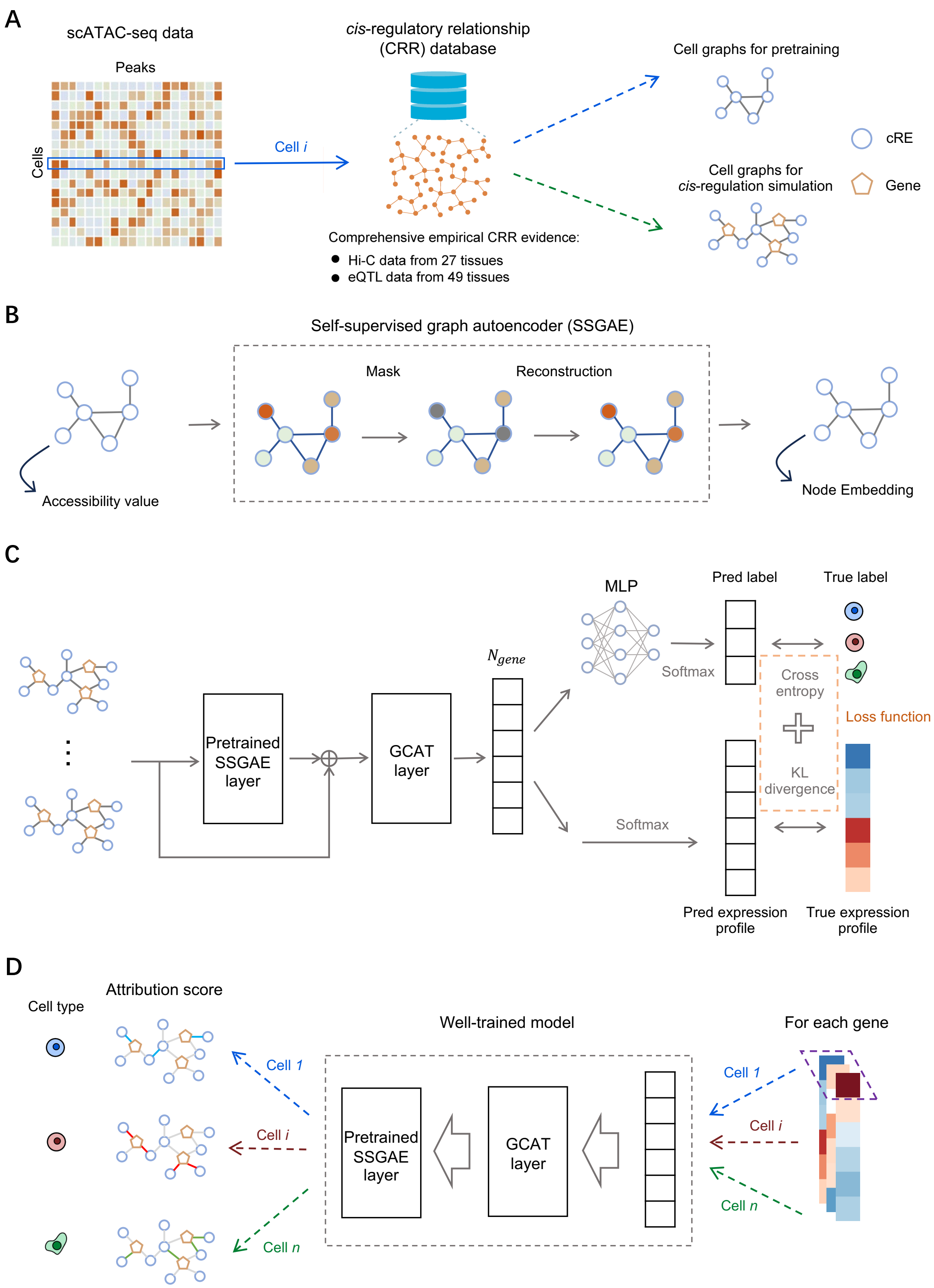

Figure S1 The workflow of SCRIPT. a, Construction of cell graphs. SCRIPT utilizes a preconstructed empirical CRR evidence database to transform scATAC-seq data into cell graphs which are used for pretraining or *cis*-regulation simulation. **b,** Pretraining of the self-supervised

graph autoencoder (SSGAE). The SSGAE is pretrained on atlas-scale datasets, enabling the transformation of node features into node embeddings. **c,** *Cis*-regulation simulation. Cell graphs processed by the pretrained SSGAE are input into a GCAT-based model to predict both cell type labels and gene expression profiles. **d,** Regulation score prediction. SCRIPT applies an attribution method to the well-trained gene expression prediction model to calculate attribution scores for each cell, which are regarded as single-cell-resolution regulation scores.

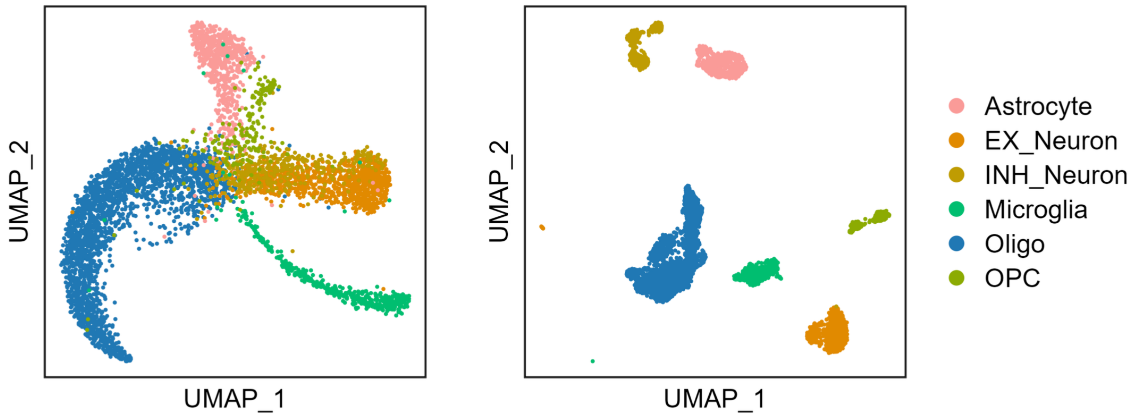

Figure S2 UMAP plots of cell embeddings generated by different methods in OlderCortex dataset. The left plot shows the UMAP plot obtained using raw chromatin accessibility. The right plot displays UMAP plot of cell embeddings generated by pretrained SSGAE. Different colors represent different cell types.

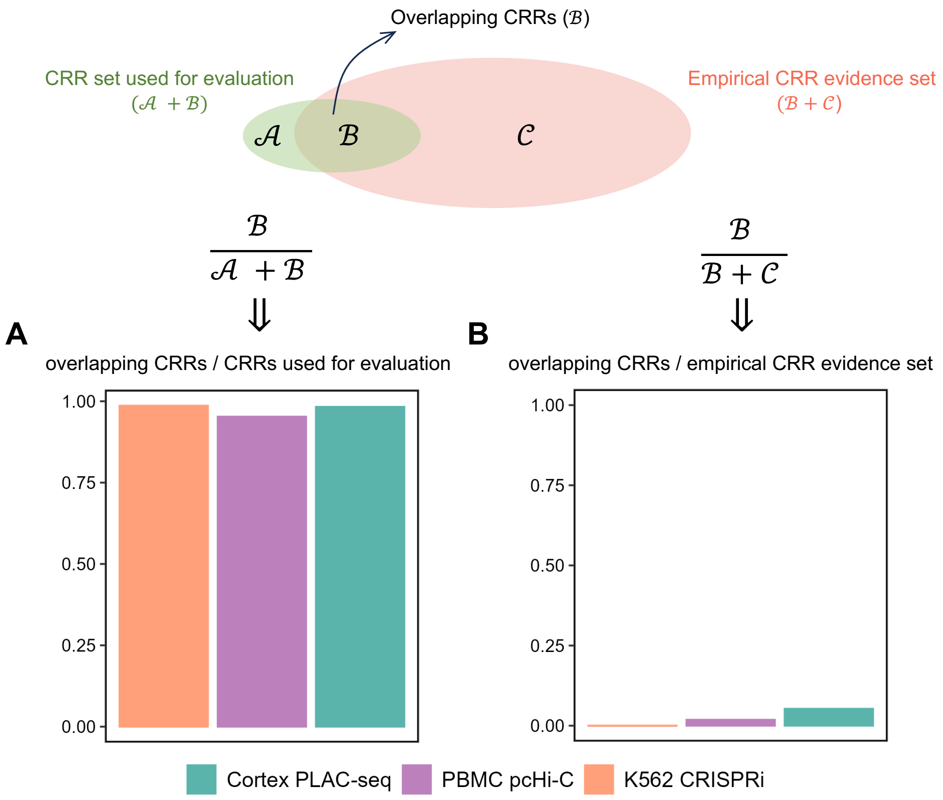

Figure S3 The analysis for the relationship between the empirical CRR evidence set and the CRR set used for model evaluation. (a) shows proportions of CRRs in the evaluation set that overlap with the empirical CRR evidence set. **(b)** shows proportions of overlapping CRRs relative to the total empirical CRR evidence set used by SCRIPT. Cortex PLAC-seq, PBMC pcHi-C, and K562 CRISPRi refer to the three cell-type-specific CRR datasets used for model evaluation.

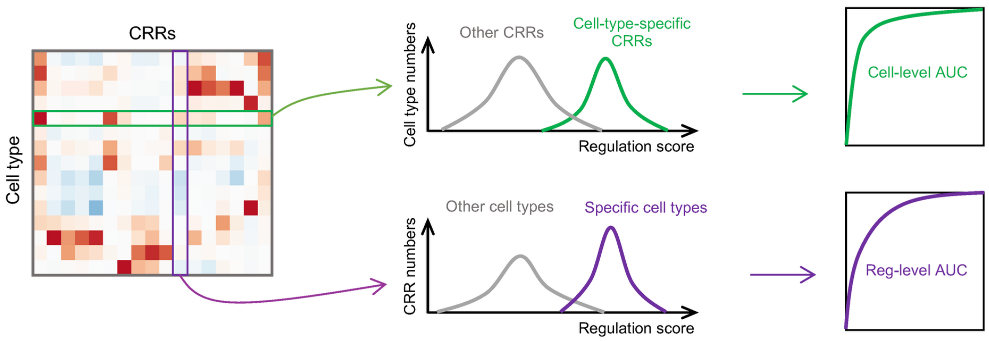

Figure S4 The diagram showing the calculation process of two metrics for assessing the effectiveness of CRR prediction methods.

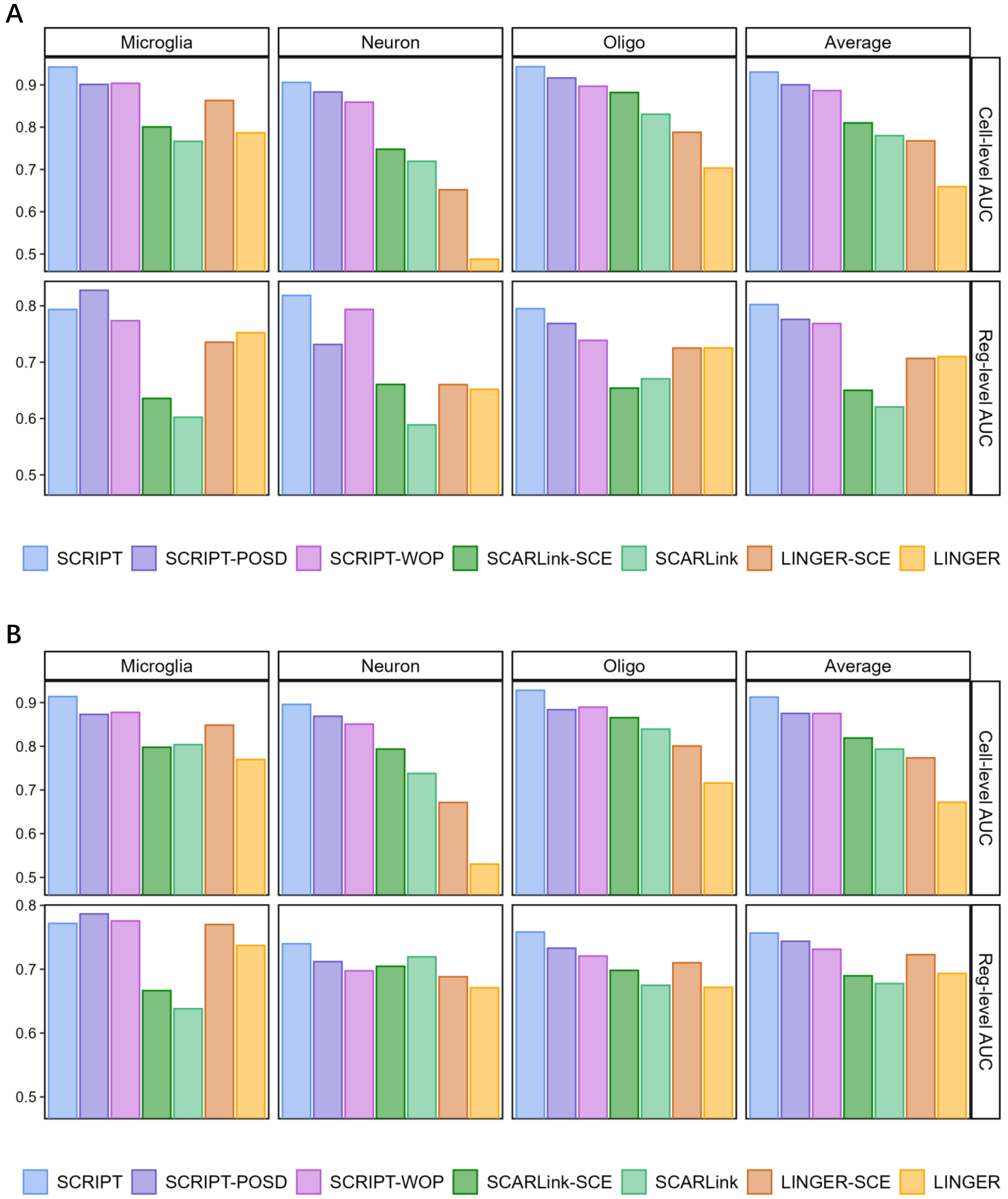

Figure S5 Performance of SCRIPT and other competing methods in different cell types. Panel **A** and **B** show the cell-level and reg-level AUCs in Cortex and OlderCortex datasets, respectively. The cell type names are shown in the boxes on top of the bar plots, the evaluation metrics are displayed in the boxes to the right of the bar plots, and different colors represent the various competing methods.

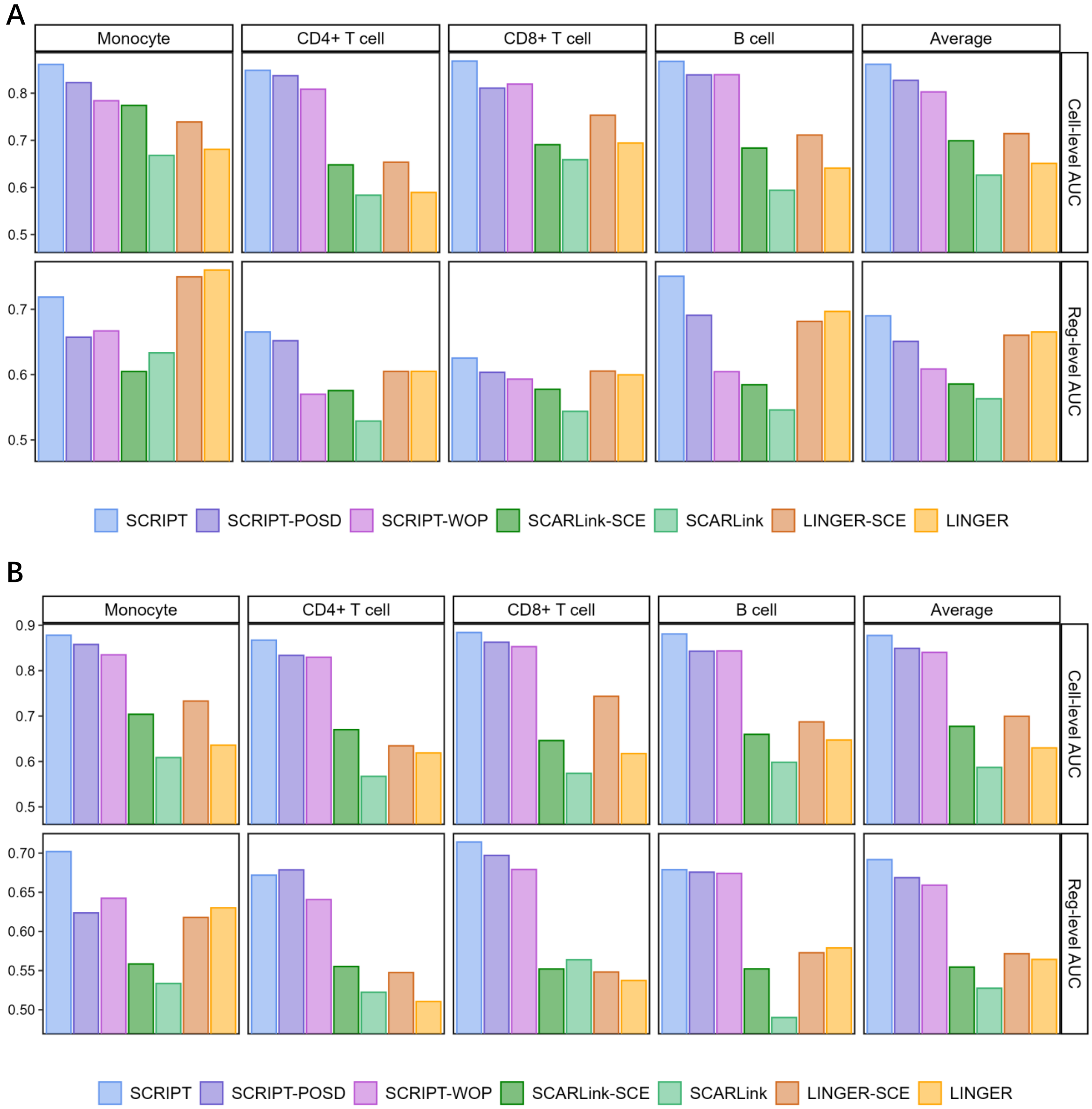

Figure S6 Performance of SCRIPT and other competing methods in different cell types. Panel A and B respectively show the performance in PBMC and PBMC&BMMC dataset. This figure is organized by the same way as **Fig. S5**.

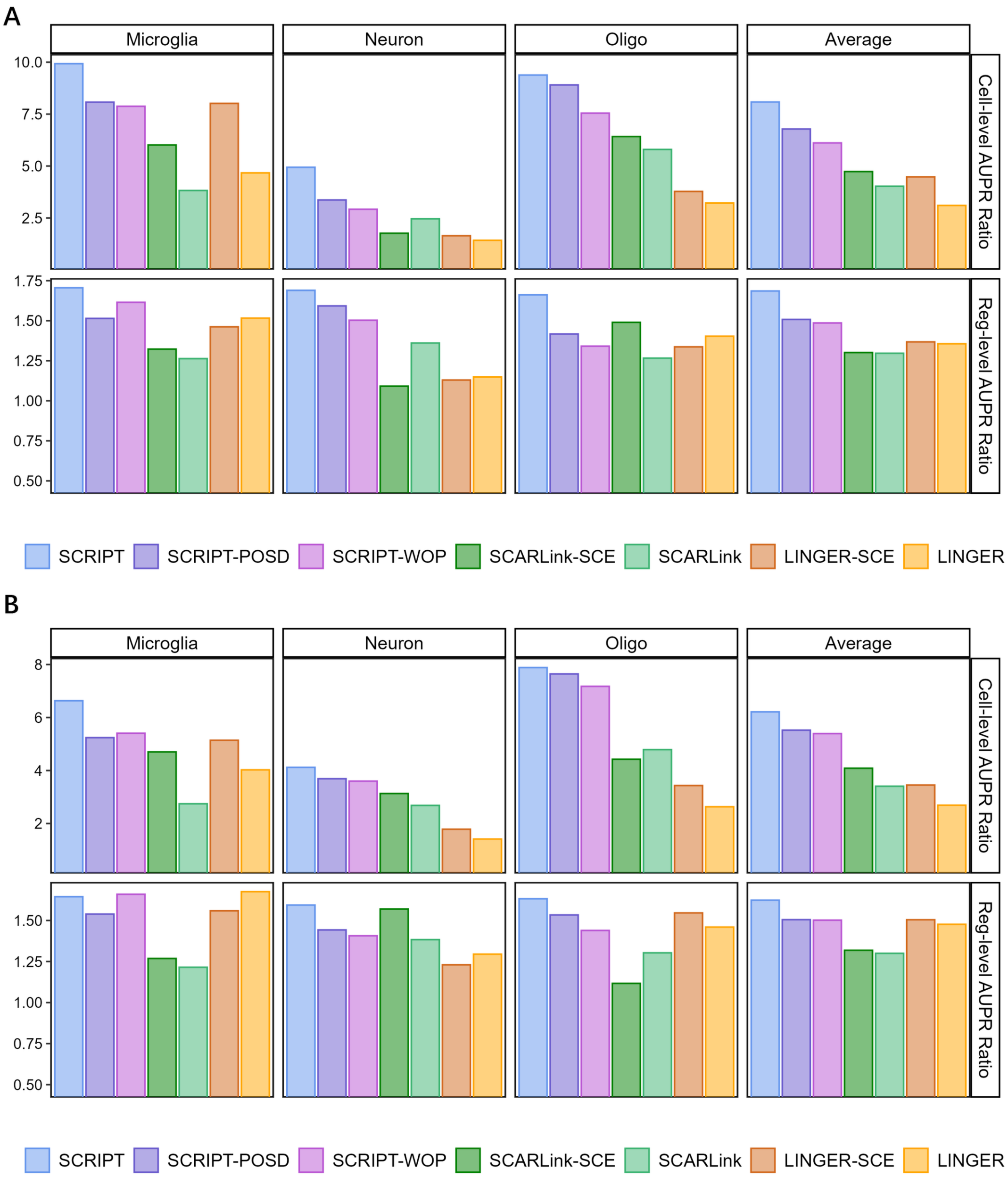

Figure S7 Performance of SCRIPT and other competing methods in different cell types. Panel **A** and **B** show the cell-level and reg-level AUPR ratios in Cortex and OlderCortex datasets, respectively. The cell type names are shown in the boxes on top of the bar plots, the evaluation metrics are displayed in the boxes to the right of the bar plots, and different colors represent the various competing methods.

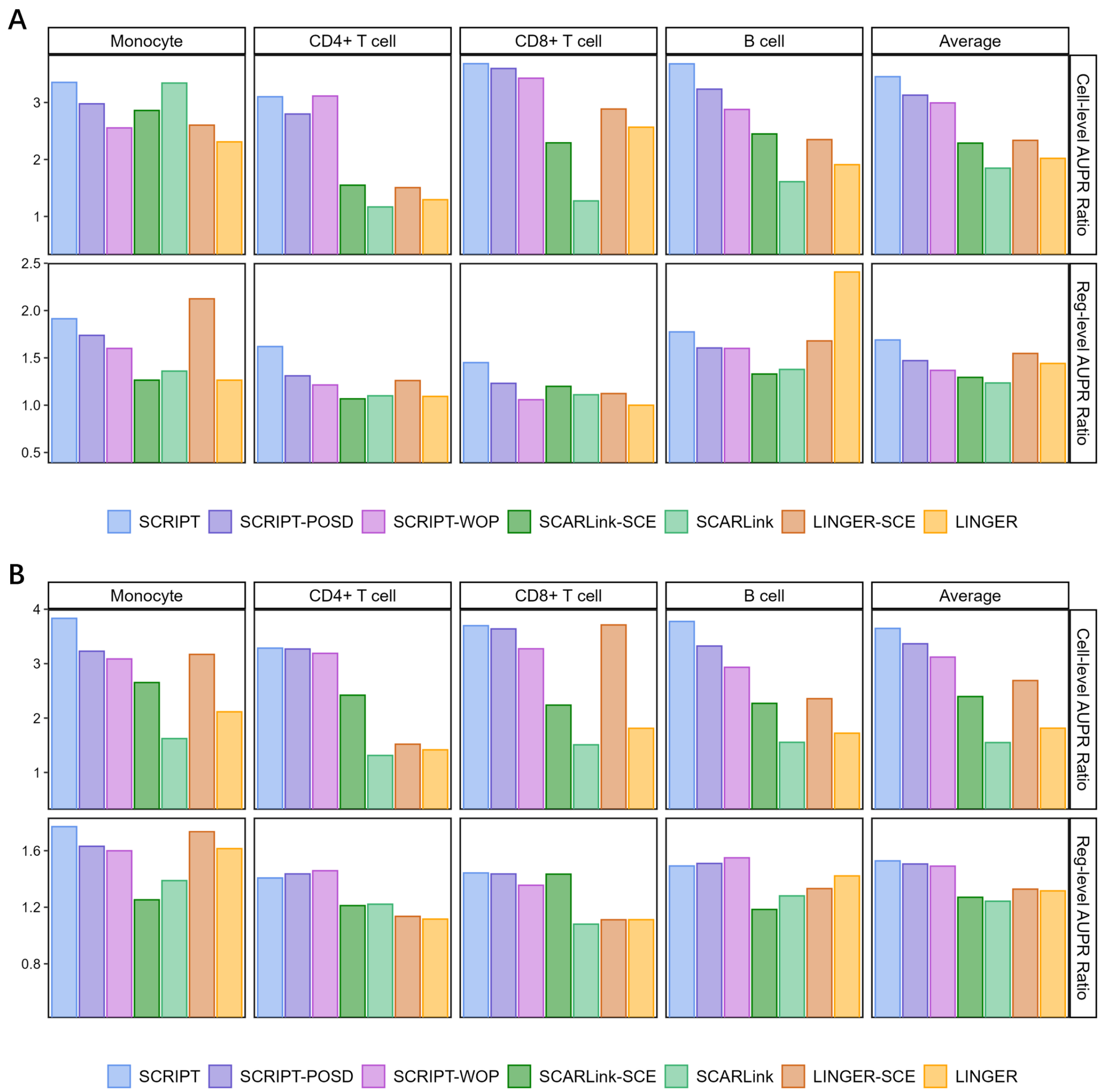

Figure S8 Performance of SCRIPT and other competing methods in different cell types. Panel A and B respectively show the performance in PBMC and PBMC&BMMC dataset. This figure is organized by the same way as **Fig. S7**.

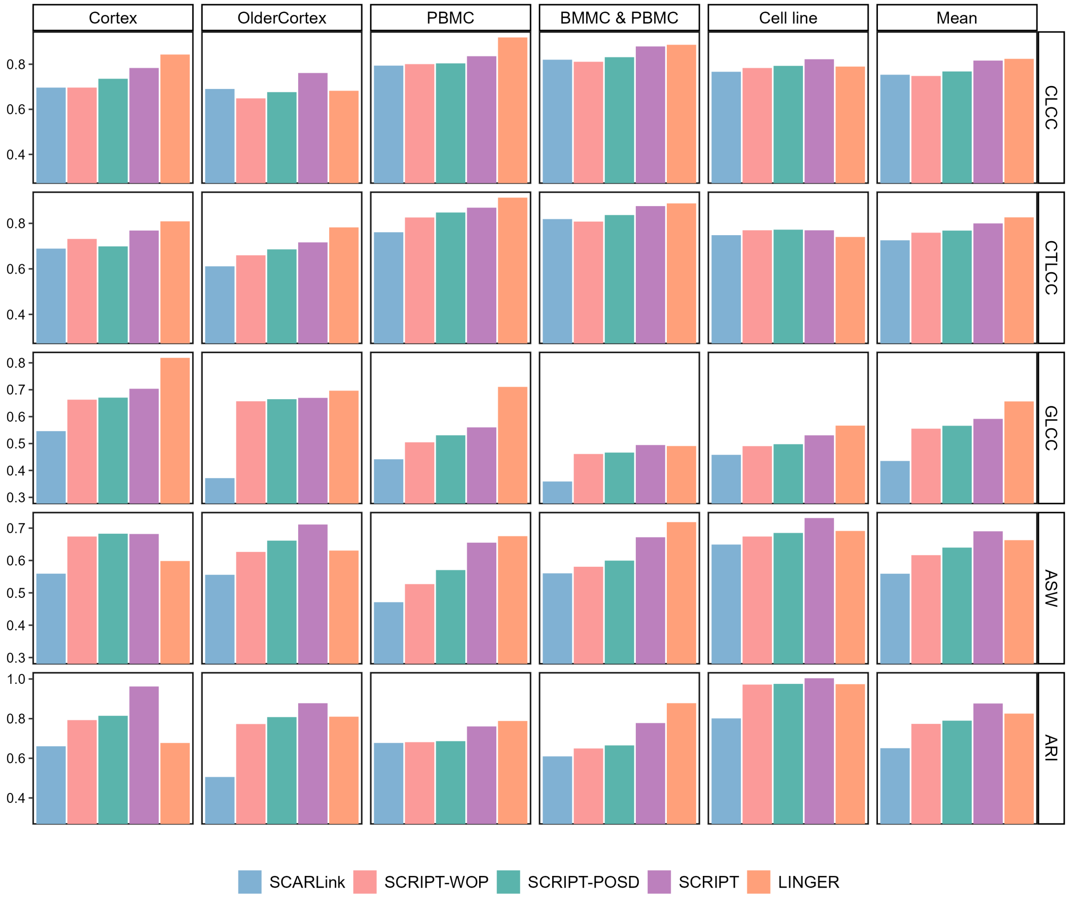

Figure S9 Performance of SCRIPT and other competing methods for gene expression prediction. The dataset names are shown in the boxes on top of the bar plots, the evaluation metrics are displayed in the boxes to the right of the bar plots, and different colors represent the various competing methods. SCRIPT-WOP, SCRIPT without pretraining; SCRIPT-POSD, SCRIPT pretrained on small dataset; CLCC, Cell-Level Correlation Coefficient; CTLCC, Cell-Type-Level Correlation Coefficient; GLCC, Gene-Level Correlation Coefficient; ASW, Average Silhouette Width; ARI, Adjusted Rand Index.

In Fig. S7, we have systematically assessed the gene expression prediction performance of SCRIPT and benchmarked it against state-of-the-art methods (SCARLink, LINGER) across five datasets. The evaluation results indicate that gene expression prediction performance is generally positively correlated with the accuracy of CRR identification, especially when model inputs are consistent.

To quantify prediction accuracy, we employed five complementary evaluation metrics: (1) Cell-Level Correlation Coefficient (CLCC), which measures per-cell correlation between predicted and observed expression profiles; (2) Cell-Type-Level Correlation Coefficient (CTLCC), which evaluates the agreement between cell-type-averaged predictions and ground truth; (3) Gene-Level Correlation Coefficient (GLCC), which measures correlation between predicted and true gene expression for each gene per cell; (4) Average Silhouette Width (ASW) and (5) Adjusted

Rand Index (ARI), both of which assess the ability of predicted expression profiles to preserve cell-type structure.

When using only scATAC-seq data as input, SCRIPT achieved the best overall performance among all models (mean CLCC: 0.81, mean CTLCC: 0.80, mean GLCC: 0.59, mean ASW: 0.69, mean ARI: 0.87). This was followed by SCRIPT-POSD (SCRIPT pretrained on small dataset; mean CLCC: 0.76, mean CTLCC: 0.76, mean GLCC: 0.56, mean ASW: 0.64, mean ARI: 0.79), SCRIPT-WOP (SCRIPT without pretraining; mean CLCC: 0.74, mean CTLCC: 0.76, mean GLCC: 0.59, mean ASW: 0.61, mean ARI: 0.77), and SCARLink (mean CLCC: 0.75, mean CTLCC: 0.72, mean GLCC: 0.43, mean ASW: 0.56, mean ARI: 0.65) (Response Figure 3).

LINGER incorporates both chromatin accessibility and transcription factor expression as input for gene expression prediction. The inclusion of additional input features may explain its slightly higher average CLCC (0.82), CTLCC (0.82) and GLCC (0.7) than SCRIPT. However, SCRIPT attained higher ASW and ARI scores, indicating that the expression profiles it predicted better captured biologically meaningful distinctions among cell types.

Overall, these results suggest a general positive correlation between gene expression prediction performance and the accuracy of CRR identification, particularly when model inputs are consistent. This supports the use of gene expression prediction accuracy as a proxy for assessing the reliability of inferred CRRs.

The computation methods for the four metrics used to assess gene expression prediction performance are detailed as follows.

CLCC is defined as the mean of Pearson correlation coefficients (PCCs) between the predicted gene expression vector of each cell and the corresponding true gene expression vector and is calculated as:

$$CLCC = \frac{1}{N}\sum_{n=1}^{N} PCC(\boldsymbol{x}_{\boldsymbol{n}}^{(\boldsymbol{pred})}, \boldsymbol{x}_{\boldsymbol{n}}^{(\boldsymbol{true})})$$

where *N* is cell numbers, $PCC(\cdot)$ is the calculation of PCC, $\boldsymbol{x}_{\boldsymbol{n}}^{(\boldsymbol{pred})}$ is the predicted gene expression vector of the *n*th cell, and $\boldsymbol{x}_{\boldsymbol{n}}^{(\boldsymbol{true})}$ is the true gene expression vector of the *n*th cell.

For CTLCC, we first convert the predicted cell-gene matrix to cell type-gene matrix by averaging the expression profiles of the cells with the same cell type and then average the PCCs between the predicted gene expression vector of each cell type and the corresponding true gene expression vector. Cell-type-level correlation is calculated as:

$$CTLCC = \frac{1}{C}\sum_{c=1}^{C} PCC(\overline{\boldsymbol{x}}_{\boldsymbol{c}}^{(\boldsymbol{pred})}, \boldsymbol{x}_{\boldsymbol{c}}^{(\boldsymbol{true})})$$

where C is the number of cell types, $PCC(\cdot)$ is the calculation of PCC, $\boldsymbol{x}_{\boldsymbol{c}}^{(\boldsymbol{pred})}$ is the predicted gene expression vector of the *c*th cell type, and $\boldsymbol{x}_{\boldsymbol{c}}^{(\boldsymbol{true})}$ is the true gene expression vector of the *c*th cell type.

GLCC is defined as the mean of Pearson correlation coefficients (PCCs) between the predicted gene expression vector of each gene (obtained by cross-validation) and the corresponding true gene expression vector and is calculated as:

$$GLCC = \frac{1}{M}\sum_{m=1}^{M} PCC(\boldsymbol{x}_{\boldsymbol{m}}^{(\boldsymbol{pred})}, \boldsymbol{x}_{\boldsymbol{m}}^{(\boldsymbol{true})})$$

where M is gene number, $PCC(\cdot)$ is the calculation of PCC, $\boldsymbol{x}_{\boldsymbol{m}}^{(\boldsymbol{pred})}$ is the predicted gene expression vector of the mth gene, and $\boldsymbol{x}_{\boldsymbol{m}}^{(\boldsymbol{true})}$ is the true gene expression vector of the mth gene.

Before calculating ASW and ARI, we first multiply the predicted expression matrix by a factor of size 10^5, then perform a log transformation and scaling for the matrix, and then use principal component analysis (PCA) to reduce the matrix to 50 dimensions.
Average silhouette width (ASW) is calculated as:

$$ASW = \frac{1}{N}\sum_{n=1}^{N} \frac{b_n - a_n}{\max(a_n, b_n)}$$

where N is cell numbers, a_n is the mean of the distances between the nth cell and other cells with the same cell type, b_n is the mean of the distances between the nth cell and other cells with the closest cell type, all distances between cells are the Euclidean distance on the space of the 50 principal components.

We first apply the k-means algorithm to divide all cells into C clusters on the space of the 50 principal components. Adjusted Rand index (ARI) is calculated as:

$$ARI = \frac{RI - E[RI]}{max(RI) - E[RI]}, RI = \frac{a + b}{\binom{N}{2}}$$

where RI is Rand index, N is cell numbers, a is the number of cell pairs whose true and cluster labels are both same and b is the number of cell pairs whose true and cluster labels are both different.

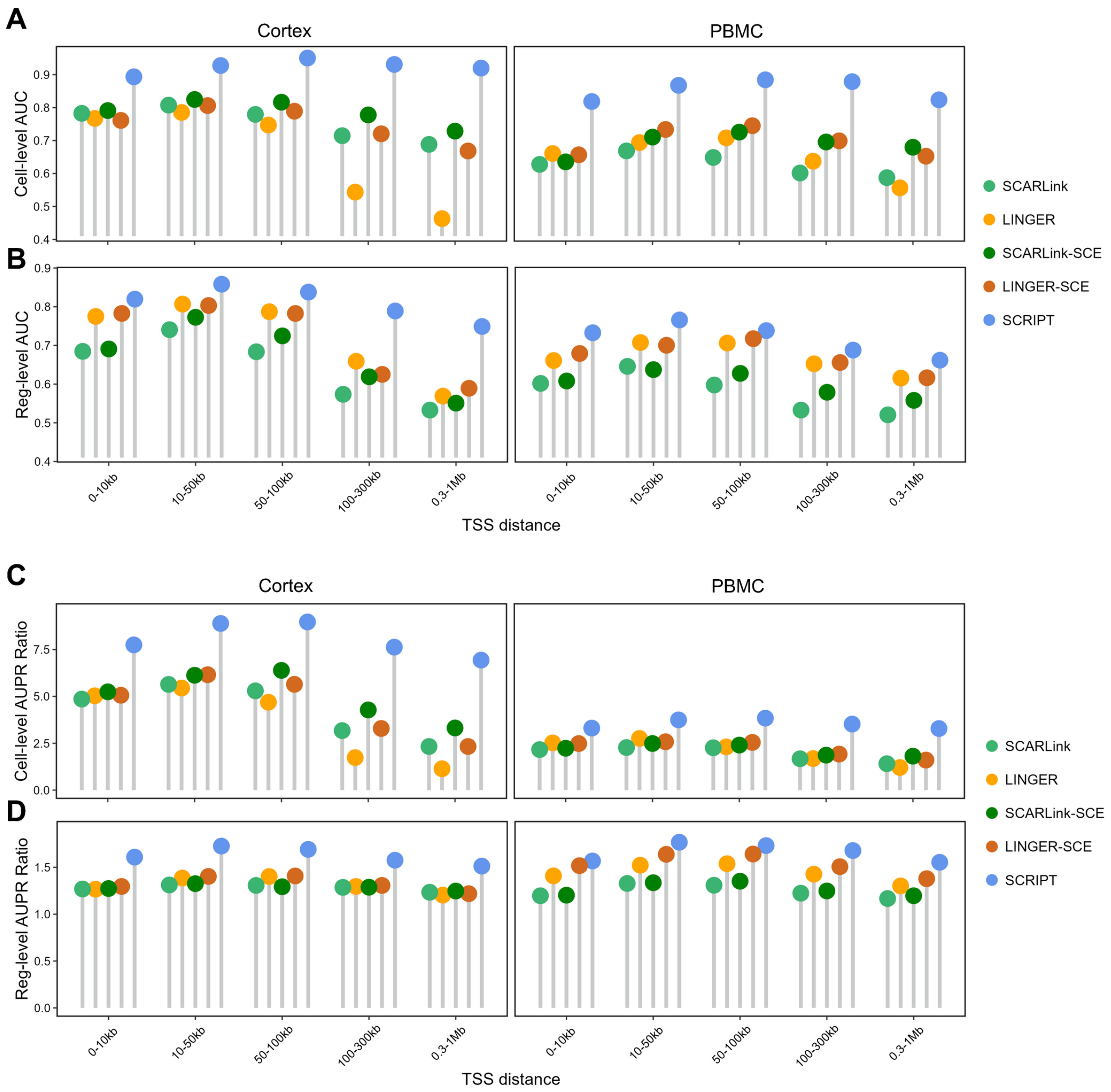

Figure S10 Performance comparison of LINGER, SCARLink, their modified versions and SCRIPT. Lollipop plots showing the cell-level AUCs (**A**), reg-level AUCs (**B**), cell-level AUPR ratios (**C**), and reg-level AUPR ratios (**D**) by the five methods on the cortex and PBMC datasets. CRRs are grouped by genomic distance into five bins, ranging from 0-10 kb to 0.3-1 Mb.

The results presented in Fig. S10 indicate that, when stratifying CRRs based on the distances between CREs and the transcription start sites (TSSs) of their corresponding target genes, the improvements in LINGER-SCE and SCARLink-SCE were most pronounced in the 100-300 kb and 300 kb-1 Mb distance ranges, compared to their original counterparts.

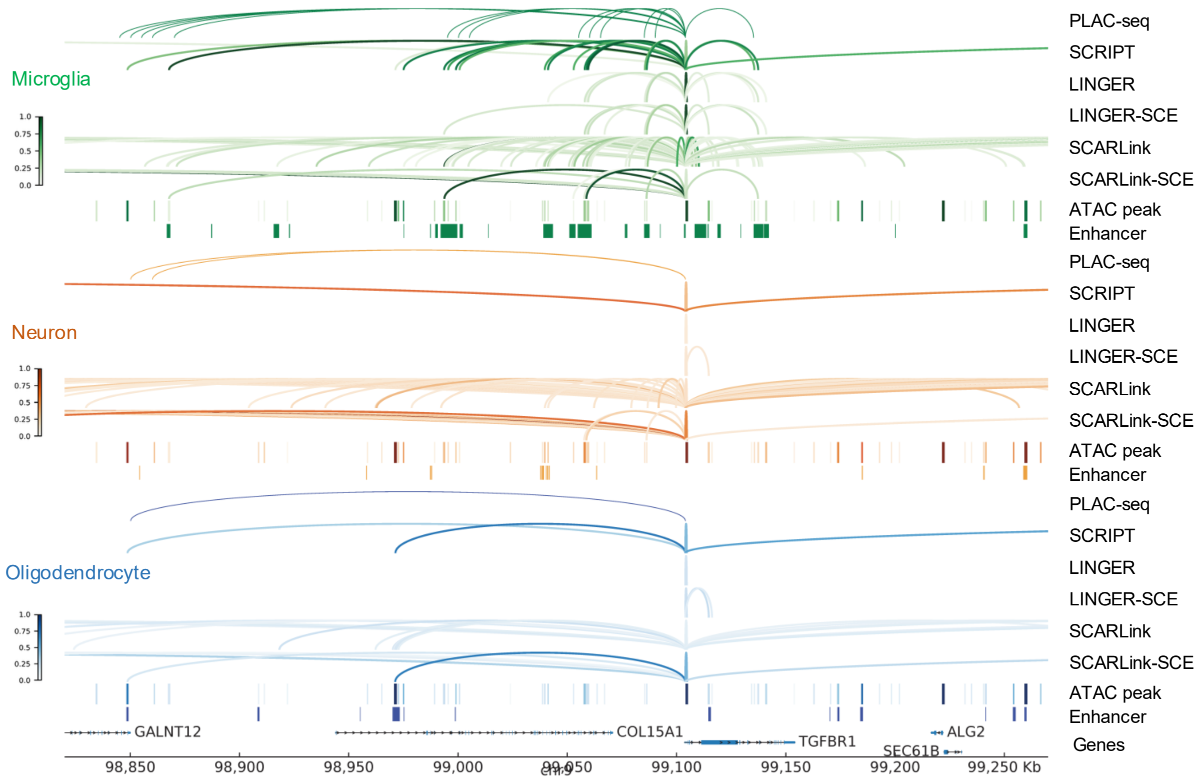

Figure S11 The comparison of cell-type-specific CRR predictions by of LINGER, SCARLink, their modified versions, and SCRIPT. This figure displays the predicted results in *TGFBR1* gene locus, and is organized by the same way as **Fig. 3c**.

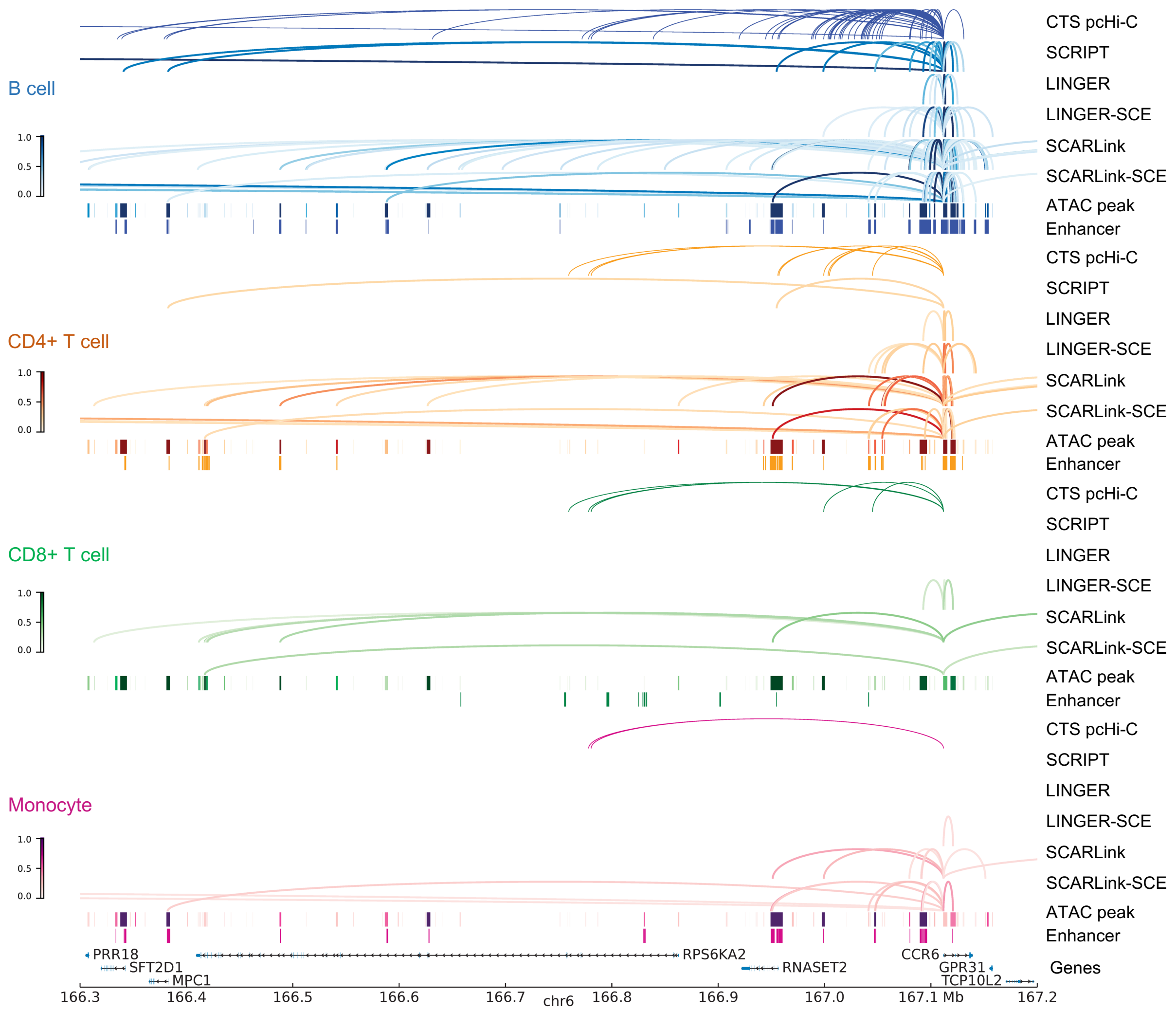

Figure S12 The comparison of cell-type-specific CRR predictions by of LINGER, SCARLink, their modified versions, and SCRIPT. This figure displays the predicted results in *CCR6* gene locus, and is organized by the same way as **Fig. 3d**.

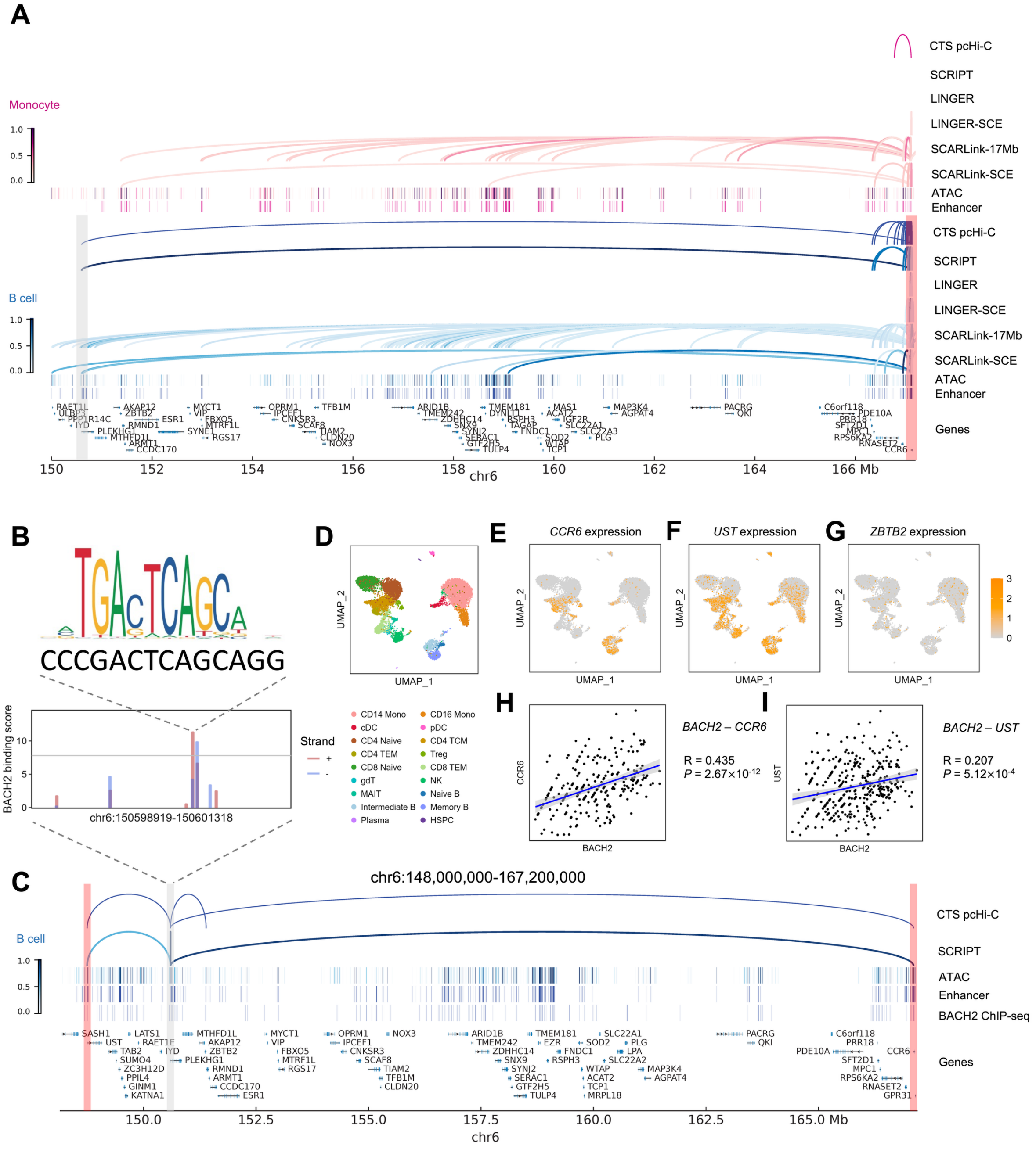

Figure S13 A exploratory result predicted by SCRIPT. (A) Genomic visualizations of CRRs identified by cell-type-specific pcHi-C data, normalized regulation scores predicted by SCRIPT, LINGER, LINGER-SCE, SCARLink-17Mb and SCARLink-SCE, normalized scATAC-seq-derived pseudobulk tracks, and enhancers identified by H3K27ac ChIP-seq data in the *CCR6* gene locus (chr6:150,000,000-167,200,000). The CREs linked to *CCR6* based on ChIP-seq and cell-type-specific pcHi-C data are highlighted in grey bars. The TSSs of *CCR6* are marked by red bars. **(B)** Bar plot illustrating the BACH2 binding scores across the enhancer region (chr6:150598919–150601318). Red and blue bars represent predicted binding scores on the sense and antisense DNA strands, respectively. The grey horizontal line indicates the JASPAR-recommended threshold for high-confidence TFBS predictions. The sequence with the highest

predicted binding score is highlighted, and the corresponding BACH2 motif is displayed. **(C)** Genomic visualizations of CRRs identified by cell-type-specific pcHi-C data, normalized regulation scores predicted by SCRIPT, normalized scATAC-seq-derived pseudobulk track, enhancers identified by H3K27ac ChIP-seq data, and BACH2 ChIP-seq track in the genomic region (chr6:148,000,000-167,200,000). The enhancer region chr6:150598919–150601318 is highlighted in grey bars. The TSSs of *UST* and *CCR6* are marked by red bars. **(D)** UMAP visualization of scRNA-seq data from the human PBMC dataset including 18 cell types (n = 10,412 cells). **(E-G)** UMAP visualization of *CCR6* (**E**), *UST* (**F**) and *ZBTB2* (**G**) expression across 18 PBMC cell types. **(H, I)** Dot plots showing the expression of *BACH2* in B cells and its relationship with *CCR6* (n = 234 cells) **(H)** and *UST* (n = 494 cells) **(I)** expression. Only cells with non-zero expression for the corresponding gene (i.e., no dropout) are shown. The blue line denotes the linear regression fitted to the points, and the shaded grey area indicates the 95% confidence interval. Two-sided t-test is used to evaluate the statistical significance of the correlation. CTS pcHi-C, cell-type-specific promoter capture Hi-C; TFBS, transcription factor binding site.

We observe that SCRIPT identifies a B cell-specific enhancer linked to CCR6 located 16.5 Mb upstream of the CCR6 TSS, which is supported by independent evidence from both cell-type-specific pcHi-C and H3K27ac ChIP-seq data (**Figure S13A**). In contrast, LINGER and SCARLink show inferior performance in predicting this ultra-long-range CRR at the CCR6 gene locus, even when we adjust their parameters or implement modifications to enable the prediction of ultra-long-range CRRs (**Figure S13A**).

To further evaluate the reliability of this exploratory observation, we perform two additional analyses:

1. Transcription Factor Analysis of the Enhancer

   Previous studies have shown that the generation of *CCR6*$^{+}$ B cells requires the transcription factor (TF) BACH2 (*Dan Suan et al., Immunity, 2017, 10.1016/j.immuni.2017.11.022; Qianwen Hu, Cell Reports, 2022, 10.1016/j.celrep.2022.111035*), suggesting that BACH2 may positively regulate *CCR6* expression in B cells. Through transcription factor binding site (TFBS) analysis, we identified BACH2 motifs within the enhancer region (chr6:150598919–150601318) (**Figure S13B**). Moreover, BACH2 ChIP-seq data (*Srividya Swaminathan et al., Nature Methods, 2013, 10.1038/nm.3247*) also show binding signals within this enhancer in B cells (**Figure S13C**). Besides, we observed a significant co-expression pattern between *BACH2* and *CCR6* in B cells (**Figure S13H**). These results support the hypothesis that BACH2 may regulate *CCR6* expression by binding to this enhancer.

2. Evaluation of Alternative Target Genes

   To address the concern regarding alternative gene targets, we analyzed all potential genes that could be regulated by this enhancer. Cell-type-specific pcHi-C data indicate that the enhancer contacts three genes: *UST*, *ZBTB2*, and *CCR6* (**Figure S13C**). Among these,

both *UST* and *CCR6* are predicted by SCRIPT to be linked to this enhancer via CRRs (**Figure S13C**). However, *ZBTB2* is expressed at very low levels in B cells, making it an unlikely target (**Figure S13D-G**). Although *UST* is genomically closer to the enhancer, *CCR6* displays higher B-cell-specific expression and stronger correlation with *BACH2* (**Figure S13H-I**). Based on the current evidence, it remains inconclusive whether this enhancer regulates *CCR6*, *UST*, or both.

Taken together, while our TF analysis suggests that the enhancer may regulate *CCR6* via BACH2 binding, the enhancer is also predicted to regulate *UST*, a gene located closer in linear genomic distance. Thus, there is not sufficient evidence to confirm the existence of the 16.5Mb enhancer-*CCR6* interaction. Given that ultra-long-range CRRs remain a frontier topic in gene regulation, we present this result as a hypothesis that may inform future studies.

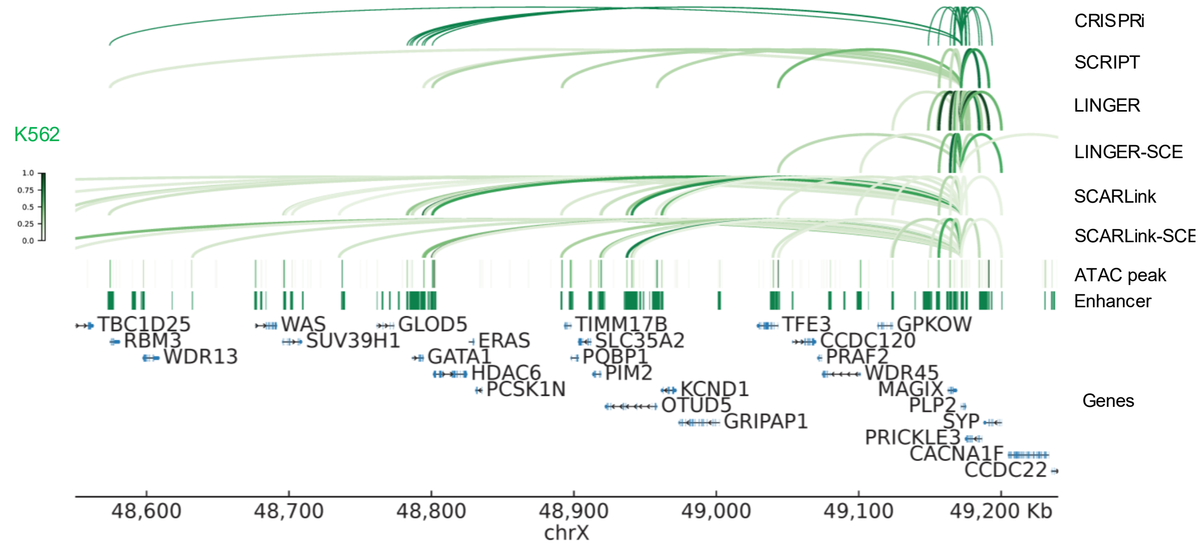

Figure S14 The comparison of cell-type-specific CRR predictions by of LINGER, SCARLink, their modified versions, and SCRIPT. This figure displays the predicted results in *PLP2* gene locus, and is organized by the same way as **Fig. 3f**.

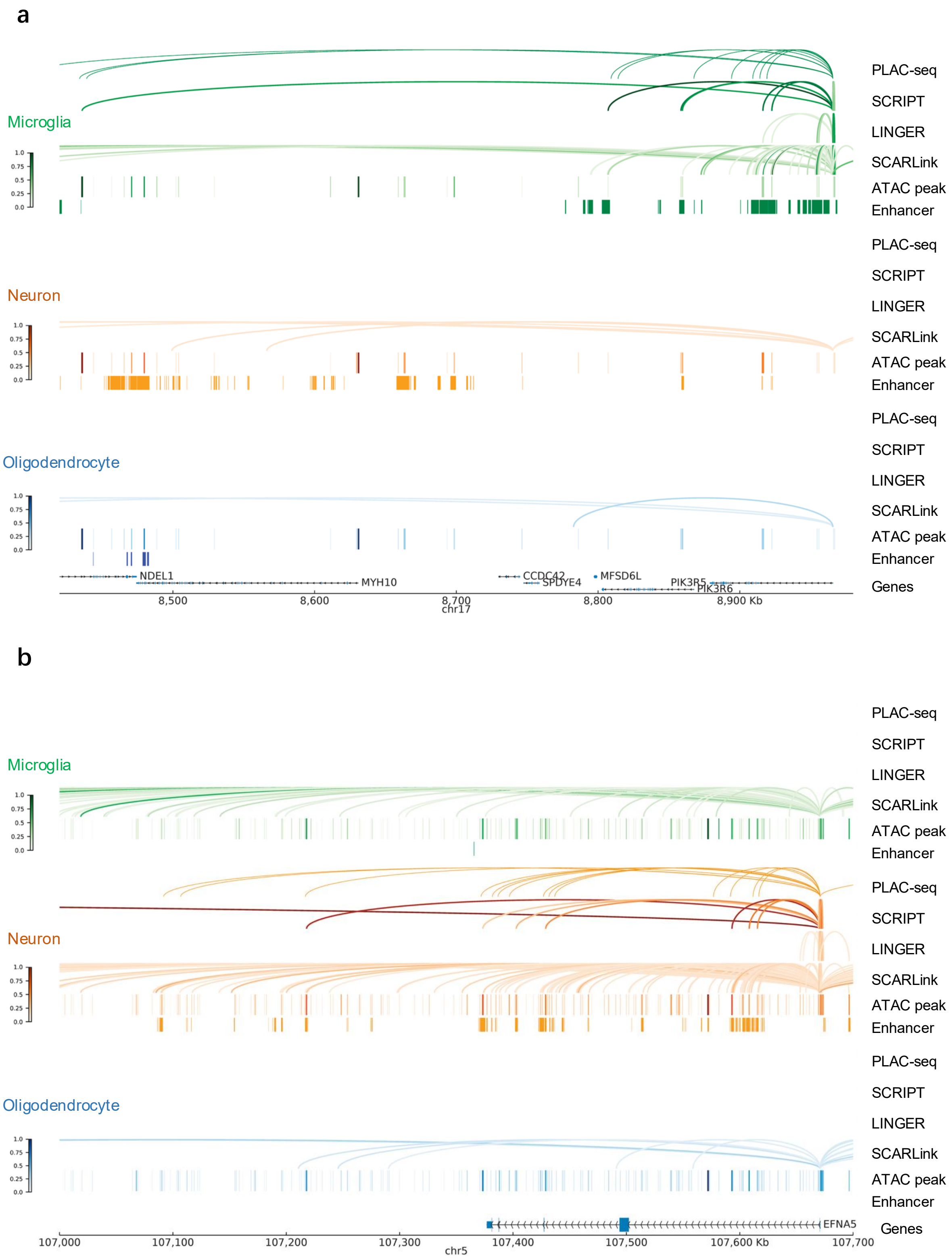

Figure S15 The comparison of cell-type-specific CRR predictions by SCRIPT, LINGER and SCARLink. Panel a and b respectively display the predicted results in *PIK3R5* and *EFNA5* gene locus. The two panels are organized by the same way as **Fig. 3c**.

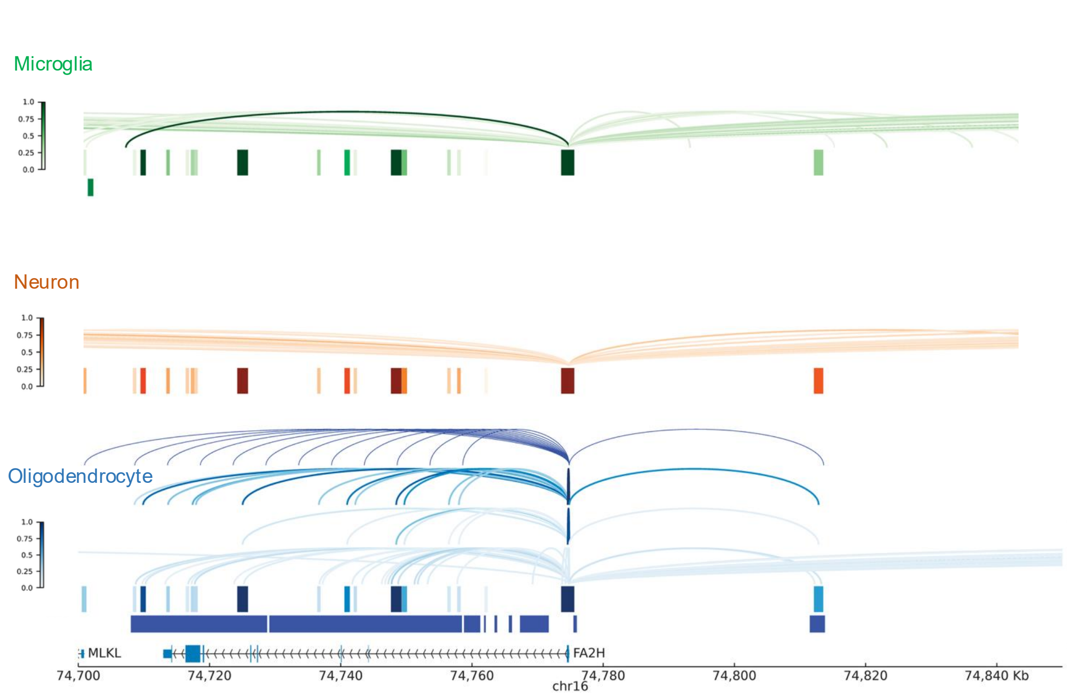

Figure S16 The comparison of cell-type-specific CRR predictions by SCRIPT, LINGER and SCARLink. This figure displays the predicted results in *FA2H* gene locus, and is organized by the same way as **Fig. 3c**.

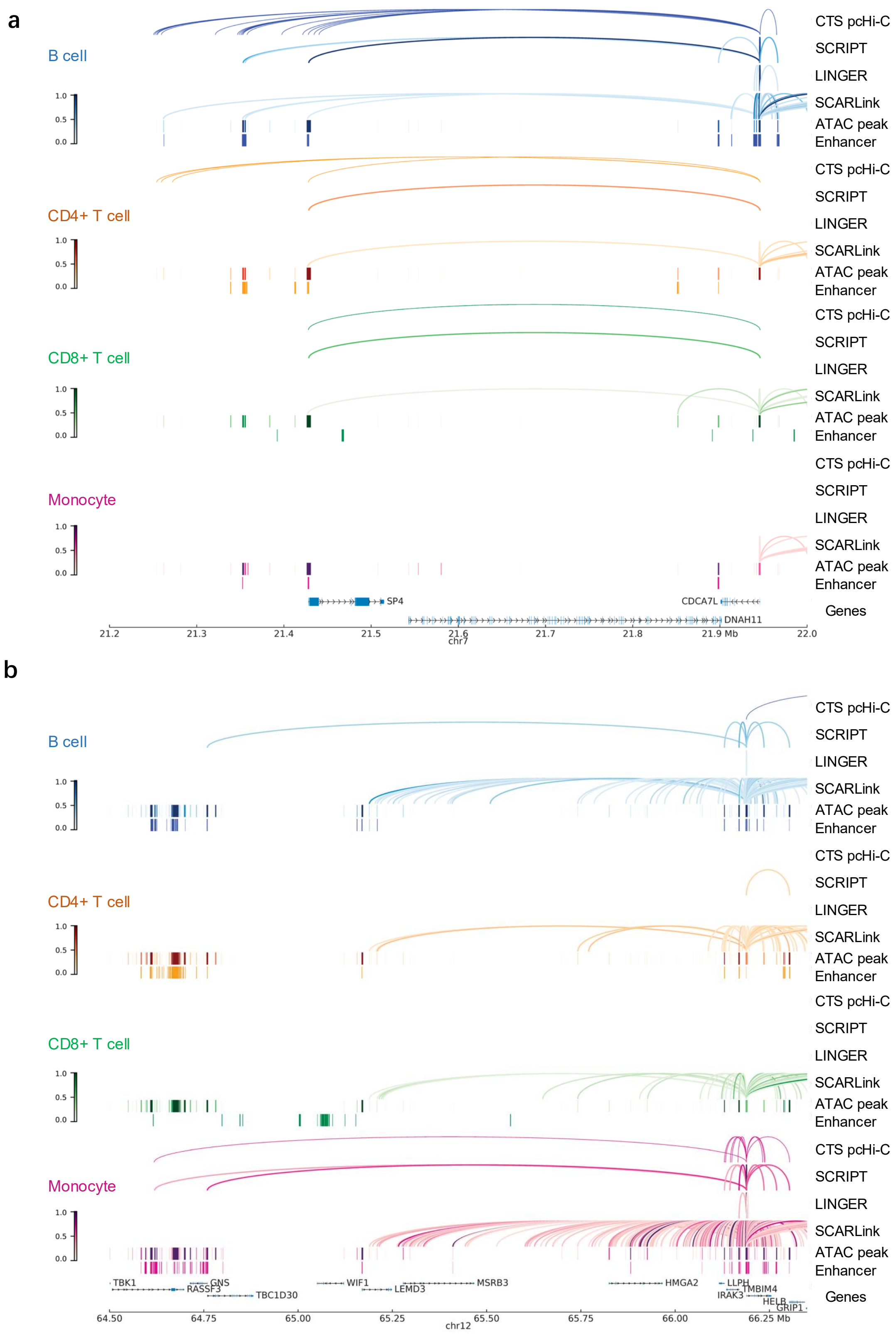

Figure S17 The comparison of cell-type-specific CRR predictions by SCRIPT, LINGER and SCARLink. Panel a and b respectively display the predicted results in *CDCA7L* and *IRAK3* gene locus. The two panels are organized by the same way as **Fig. 3d**.

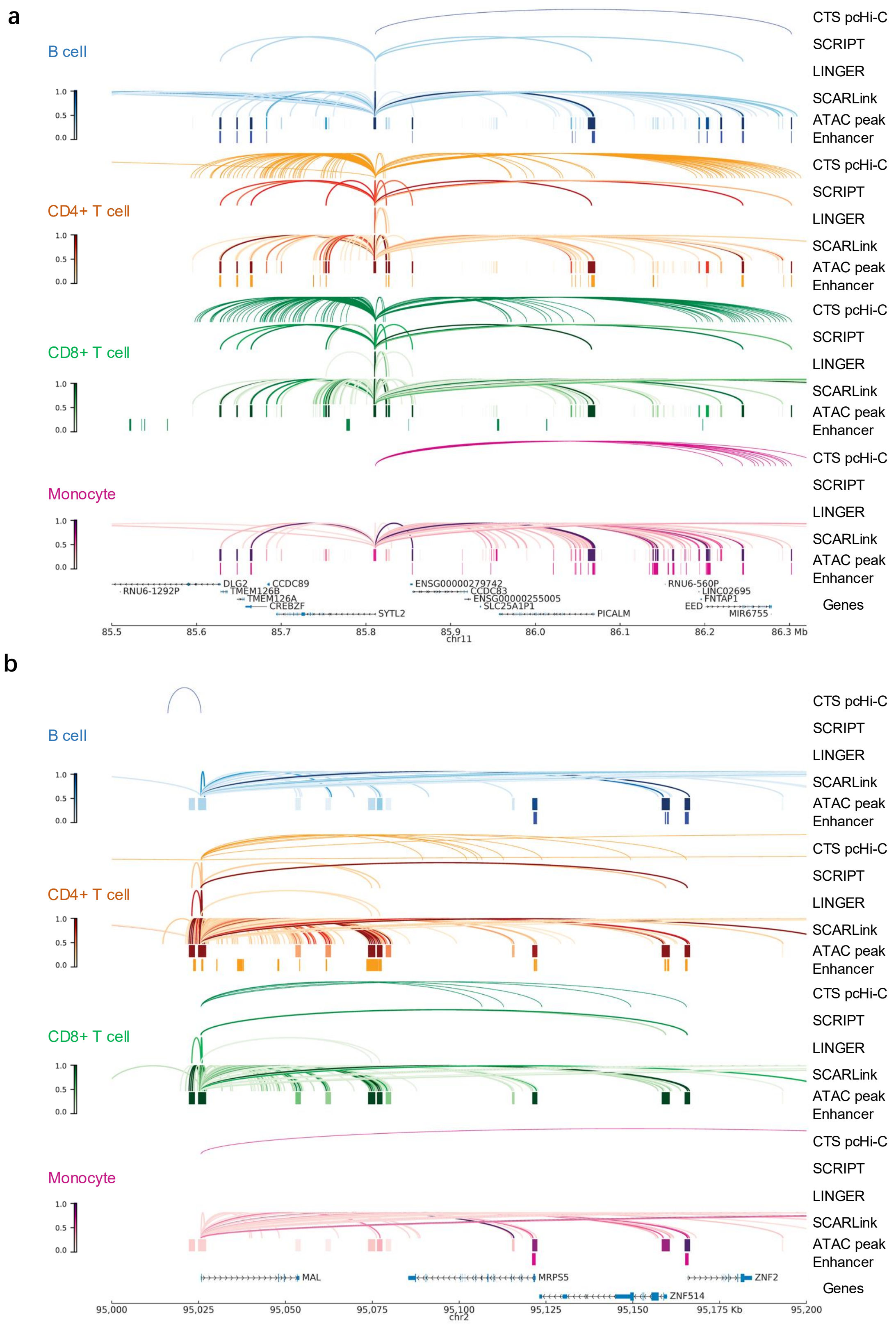

Figure S18 The comparison of cell-type-specific CRR predictions by SCRIPT, LINGER and SCARLink. Panel a and b respectively display the predicted results in *SYTL2* and *MAL* gene locus. The two panels are organized by the same way as **Fig. 3d**.

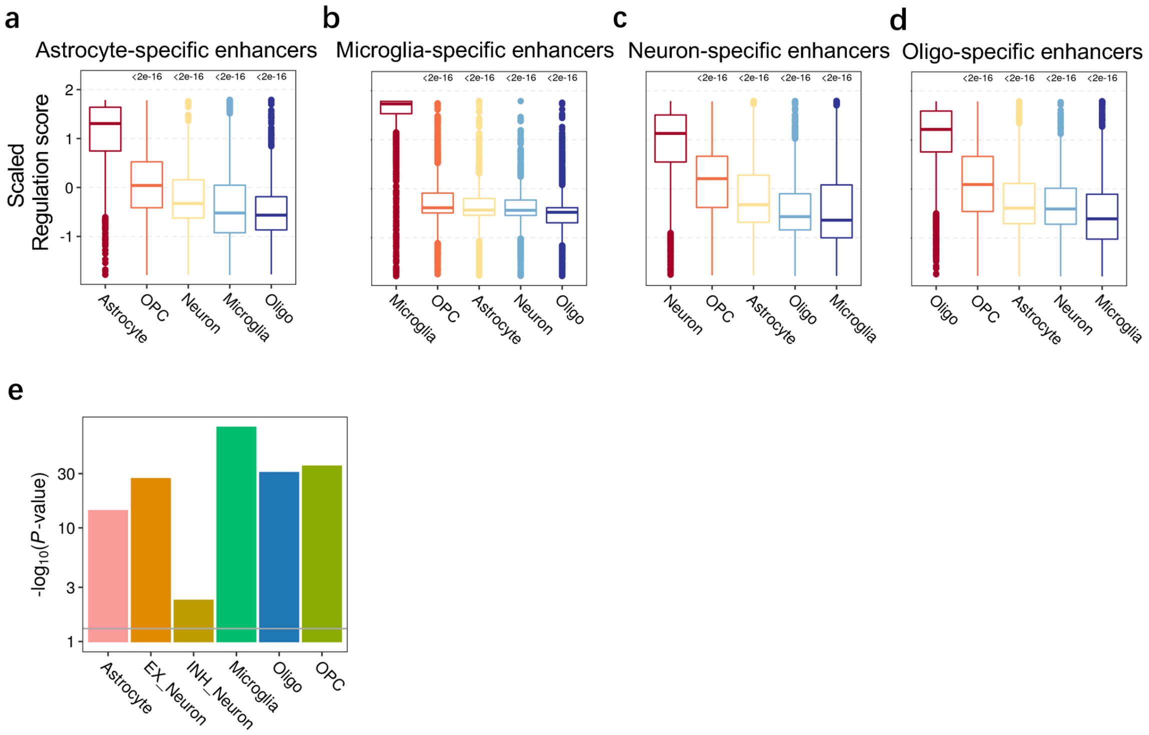

Figure S19 SCRIPT prioritizes cell-type-specific enhancers in Cortex dtatset. a-d, Boxplots of scaled regulation scores of each human cortex cell type at astrocyte-specific **(g)**, microglia-specific **(h)**, neuron-specific **(i)**, oligodendrocyte-specific **(j)** enhancers. **e**, barplots show the significance level of the overlap of cell-specific regulated genes with marker genes for each cell type in Cortex dataset.

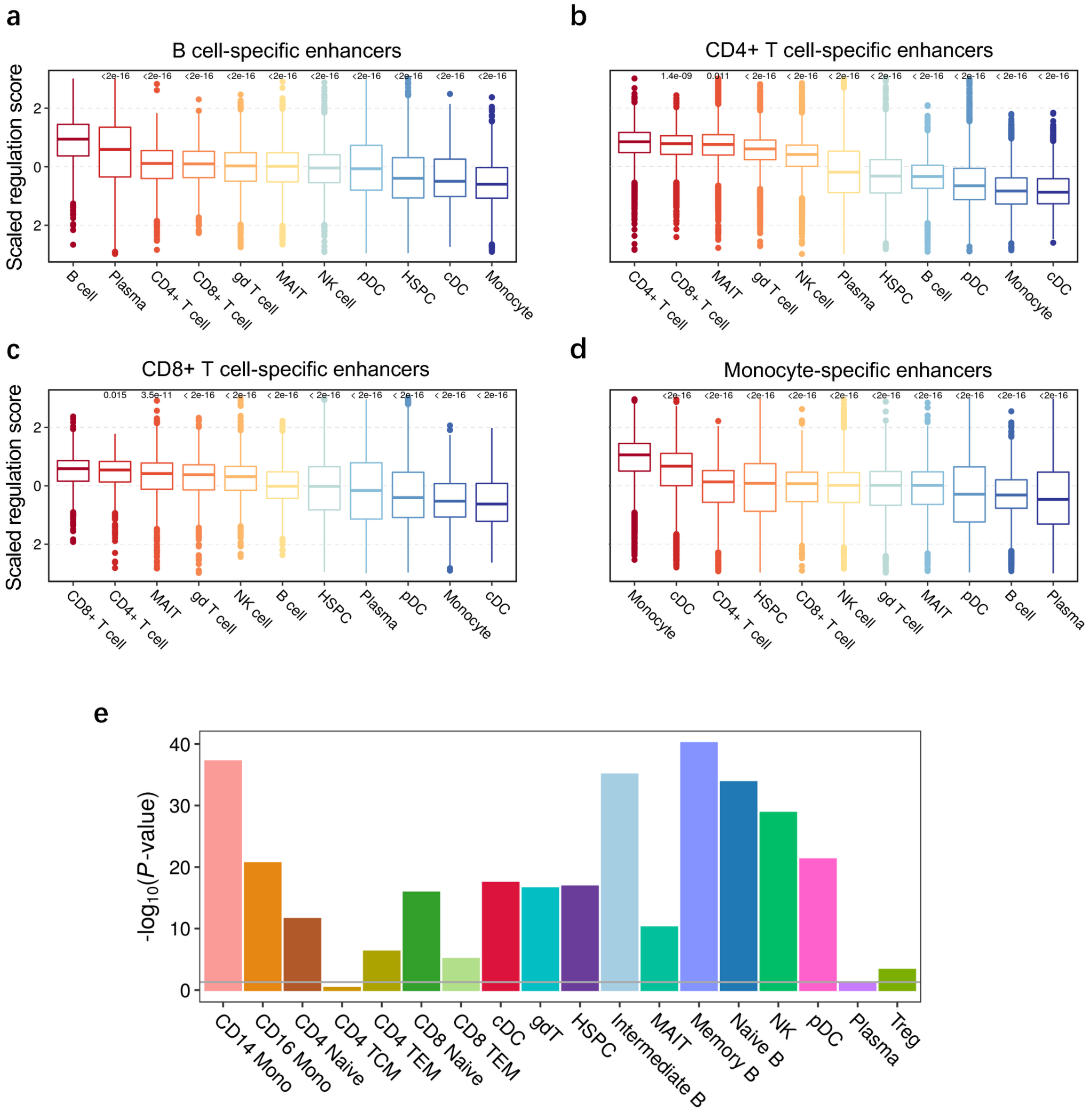

Figure S20 SCRIPT prioritizes cell-type-specific enhancers in PBMC dataset. a-d, Boxplots of scaled regulation scores of each human immune cell type at B-cell-specific **(g)**, CD4+-T-cell-specific **(h)**, CD8+-T-cell-specific **(i)**, Monocyte-specific **(j)** enhancers. **e**, Barplots show the significance level of the overlap of cell-specific regulated genes with marker genes for each cell type in PBMC datatset.

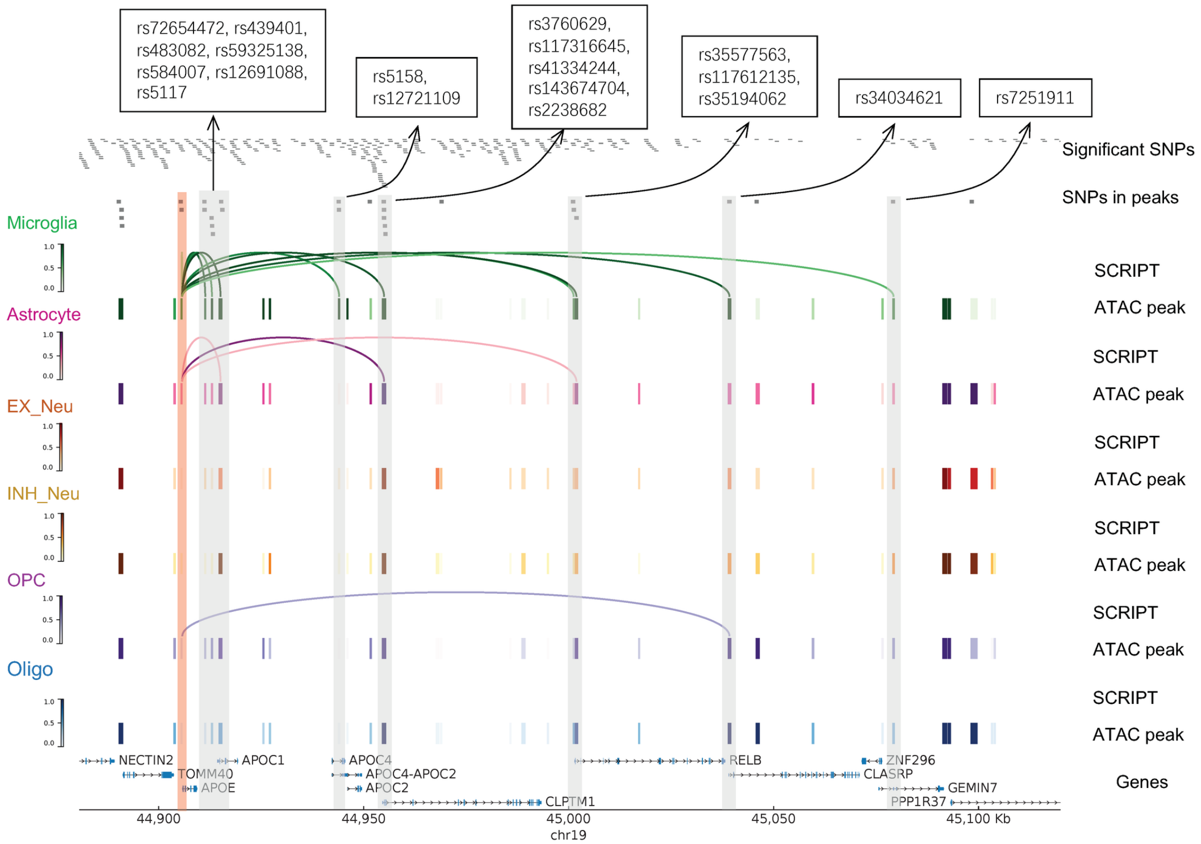

Figure S21 Application of SCRIPT to human cortex dataset for the interpretation of AD-related SNPs. a, The gene locus of *APOE* (chr19:44,880,000-45,120,000) show the normalized regulation scores predicted by SCRIPT and normalized pseudobulk accessibility scores of open chromatin regions derived from scATAC-seq for six kinds of cortex cell types. Besides, the SNPs significantly related to AD and located at CREs are displayed on the top of this panel. The rsID numbers of the SNPs which influence the expression level of *APOE* through microglia- or astrocyte-specific CRRs are highlighted in the rectangular boxes. The genomic locations of SNPs and *APOE* TSS are highlighted by grey and red bars, respectively. **b,** This panel is organized by the same way as panel a except that *SATB2* gene locus (chr2:199,200,000-200,500,000) and SCZ-related SNPs are shown.

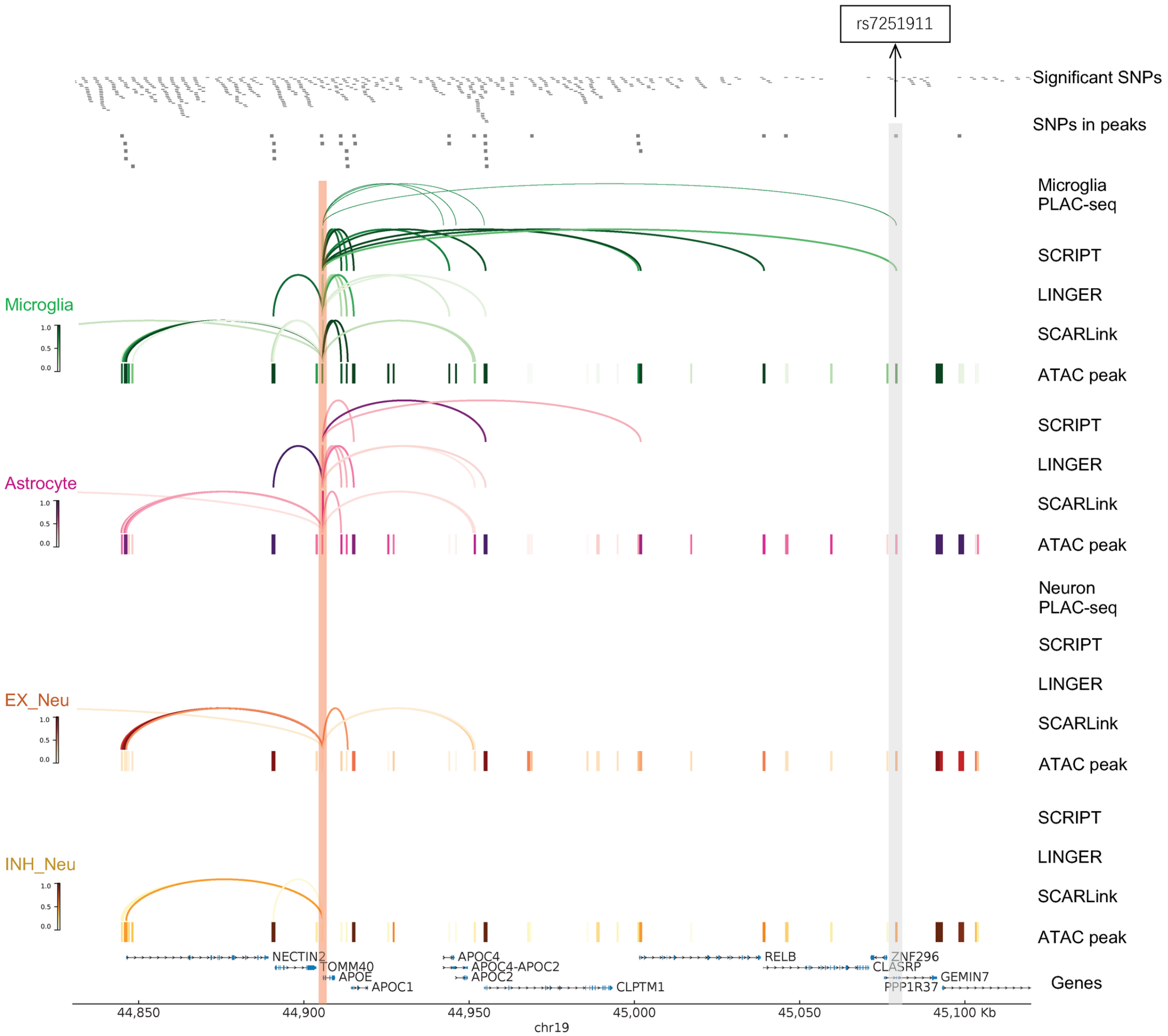

Figure S22 SCRIPT explaining the pathogenic mechanism of AD-related SNPs undetected by LINGER and SCARLink. The *APOE* gene locus (chr19:44,880,000-45,120,000) shows the CRRs identified by PLAC-seq in microglia and neuron, regulation scores predicted by SCRIPT, LINGER and SCARLink and normalized scATAC–seq-derived pseudobulk tracks across four cell types. SNPs significantly associated with AD and located within CREs are displayed at the top of this panel. The genomic locations of SNPs of interest and the *APOE* transcription start site (TSS) are highlighted by gray and red bars, respectively. This figure demonstrates that only SCRIPT assigns rs7251911 to *APOE*, which is consistent with the PLAC-seq data.

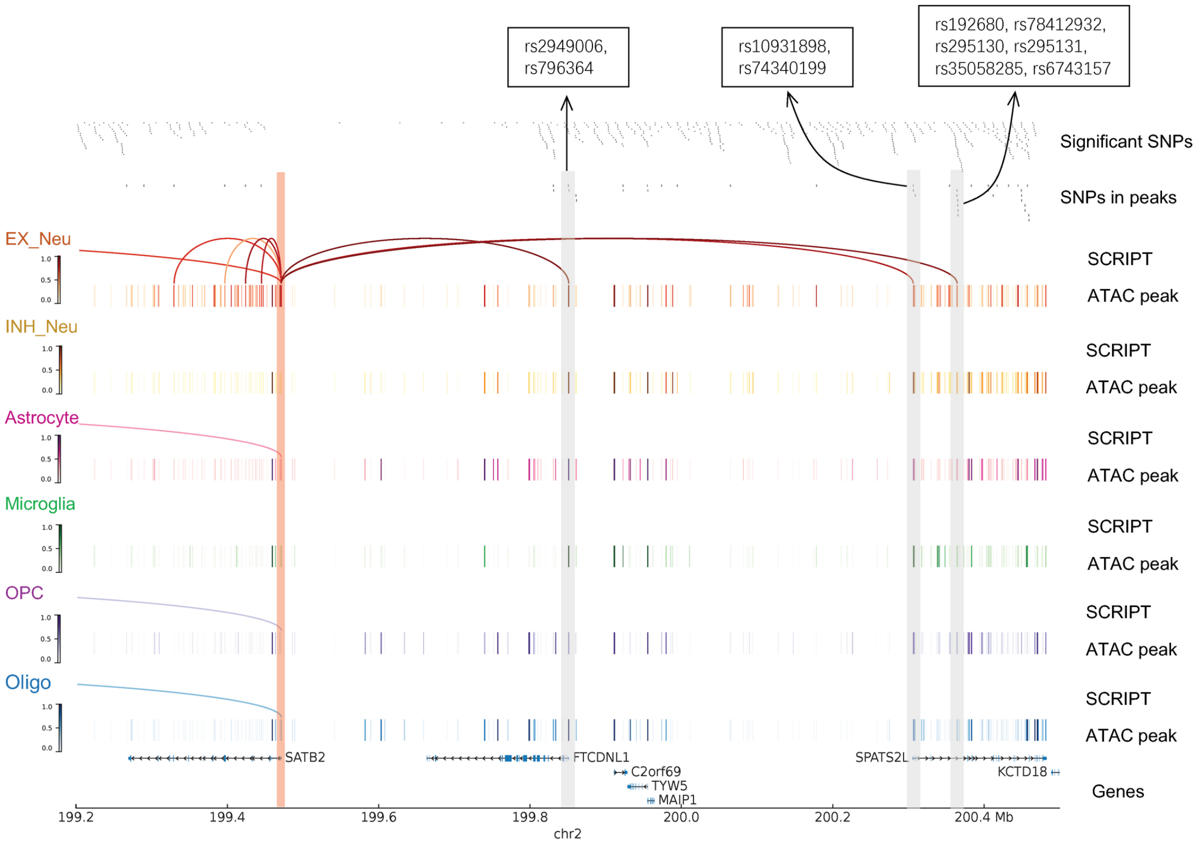

Figure S23 Application of SCRIPT to human cortex dataset for the interpretation of SCZ-related SNPs. This figure is organized by the same way as **Figure S21** except that *SATB2* gene locus (chr2:199,200,000-200,500,000) and SCZ-related SNPs are shown.

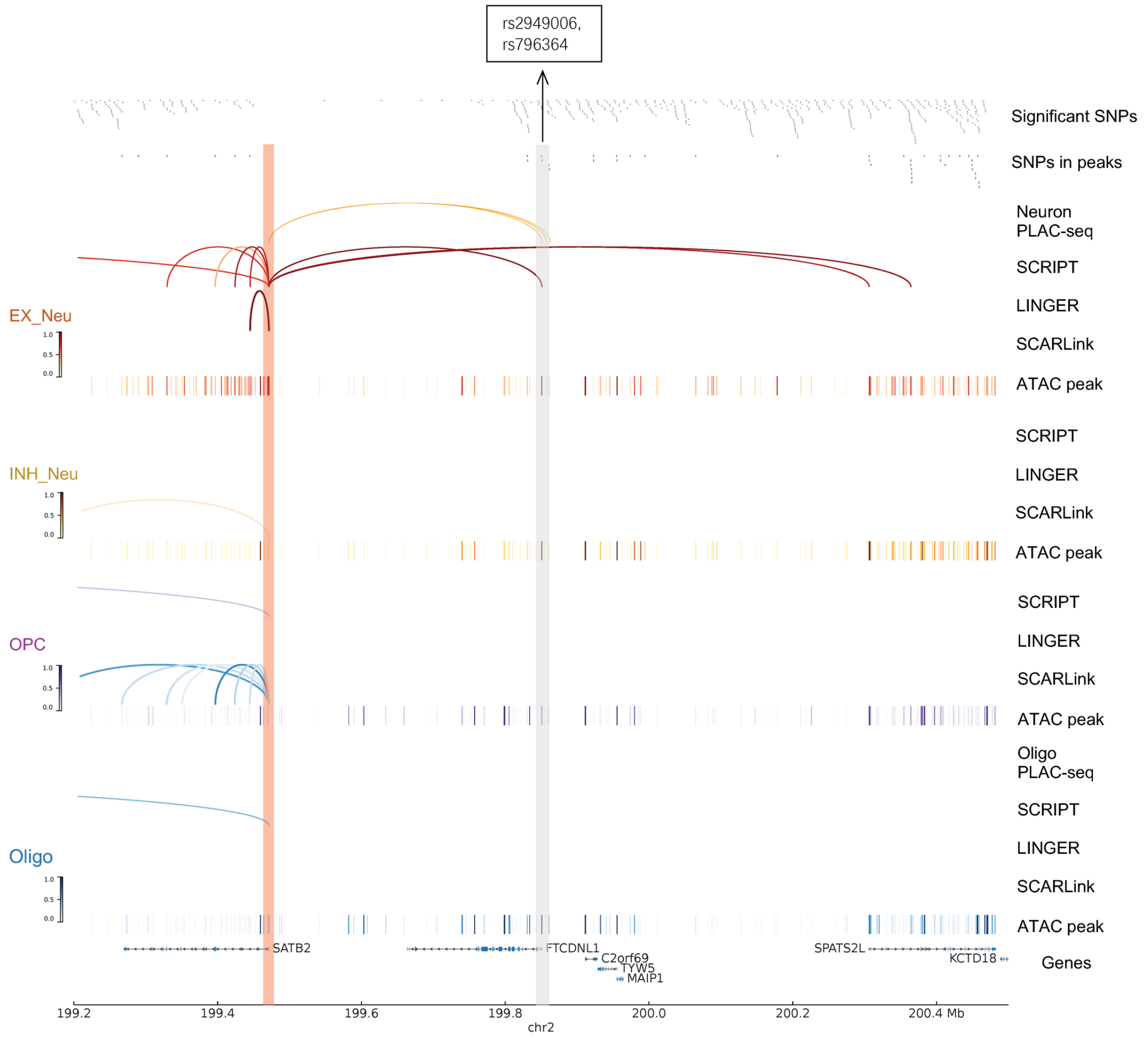

Figure S24 SCRIPT explaining the pathogenic mechanism of SCZ-related SNPs undetected by LINGER and SCARLink. The *SATB2* gene locus (chr2:199,200,000-200,500,000) shows the CRRs identified by PLAC-seq in neuron and oligodendrocyte, regulation scores predicted by SCRIPT, LINGER and SCARLink and normalized scATAC–seq-derived pseudobulk tracks across four cell types. SNPs significantly associated with AD and located within CREs are displayed at the top of this panel. The genomic locations of SNPs of interest and the *SATB2* transcription start site (TSS) are highlighted by gray and red bars, respectively. This figure indicates that only SCRIPT assigns rs2949006 to *SATB2*, consistent with the PLAC-seq data.

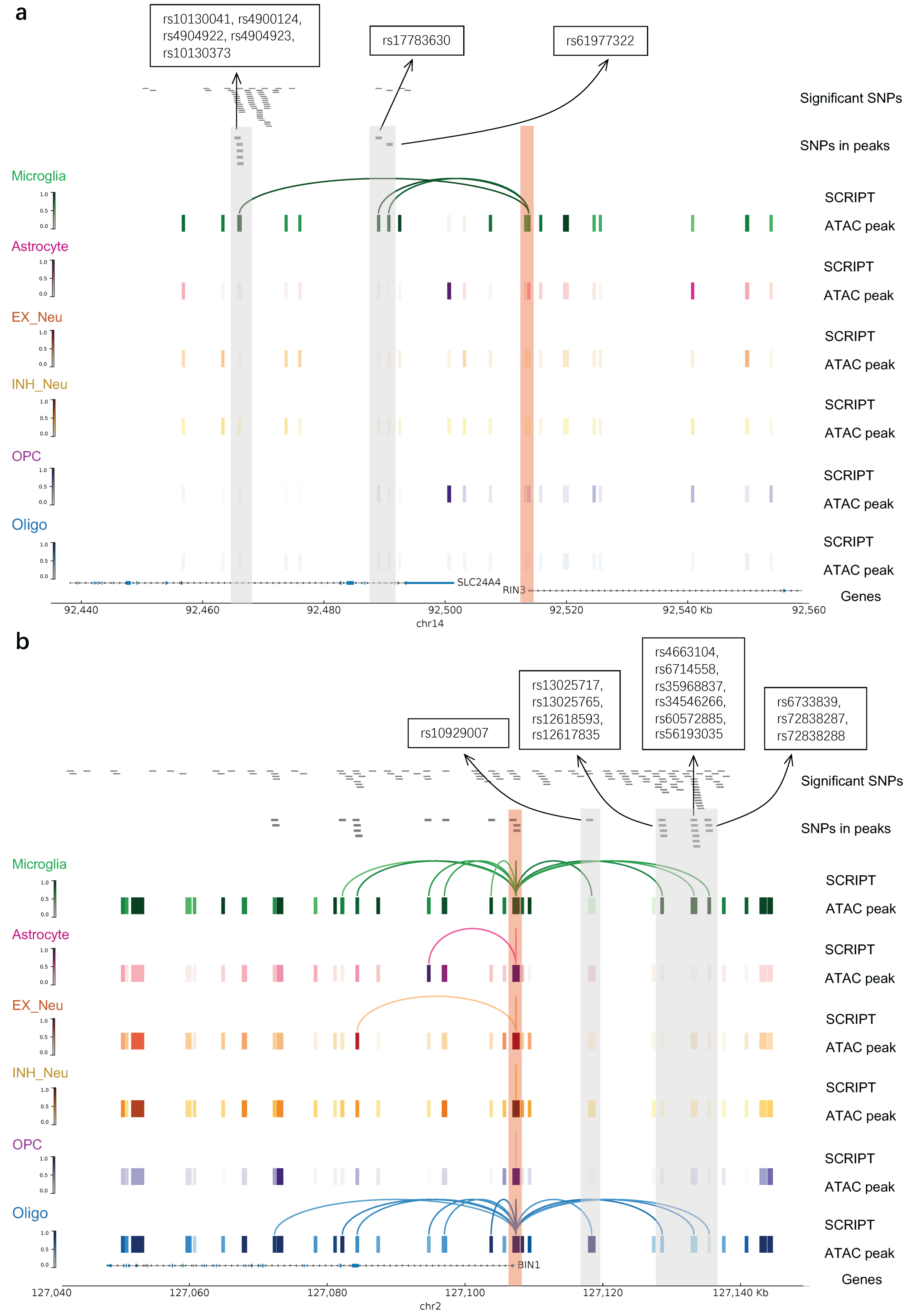

Figure S25 Application of SCRIPT to human cortex dataset for the interpretation of AD-related SNPs. Panel **a** and **b** show the results in *RIN3* and *BIN1* loci, and are organized by the same way with **Figure S21**.

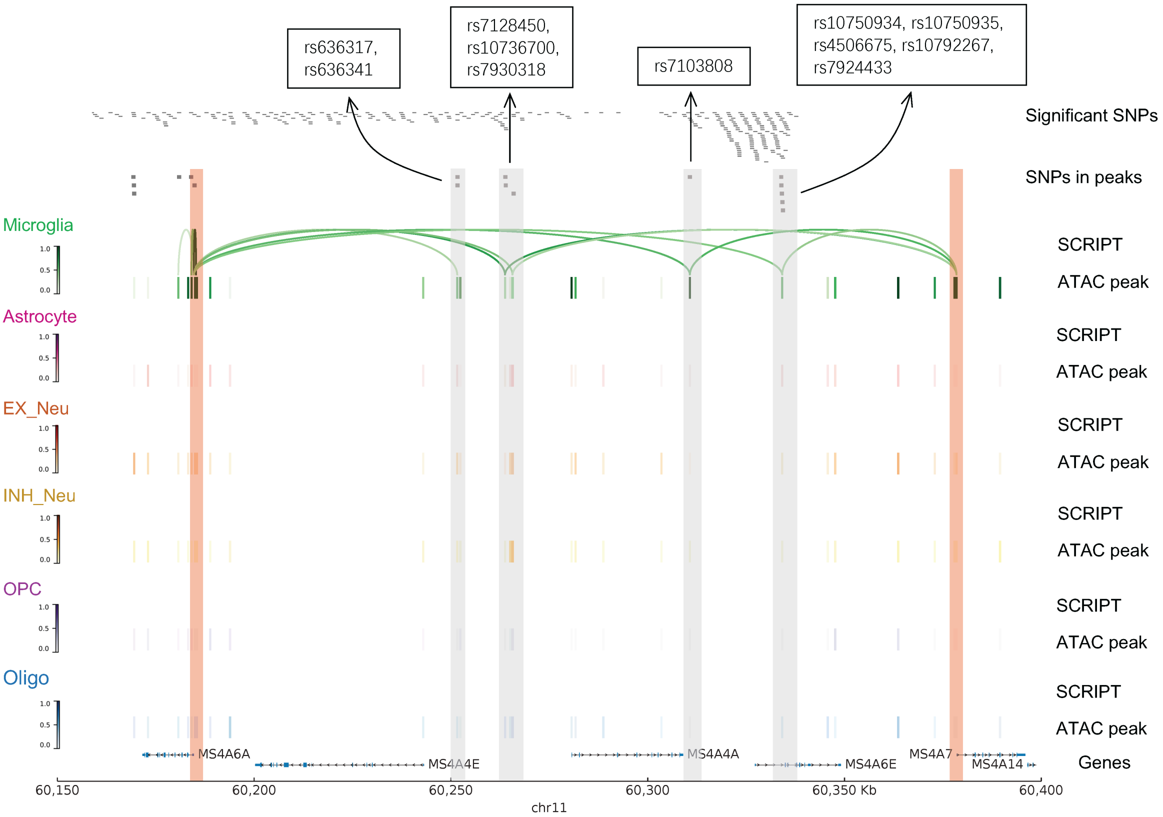

Figure S26 Application of SCRIPT to human cortex dataset for the interpretation of AD-related SNPs. This figure shows the results in *MS4A* gene cluster loci, and is organized by the same way with **Figure S21**.

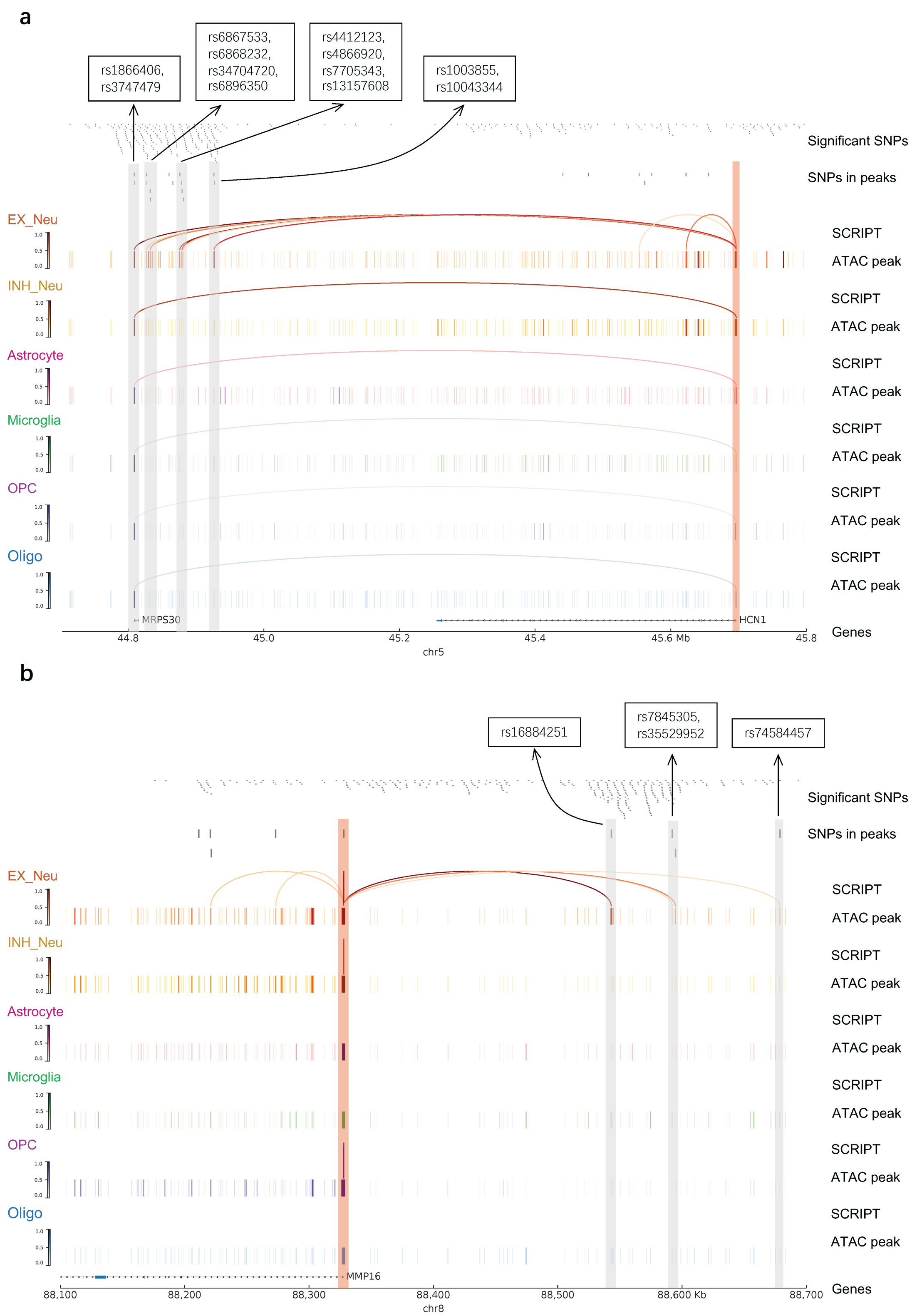

Figure S27 Application of SCRIPT to human cortex dataset for the interpretation of SCZ-related SNPs. Panel **a** and **b** show the results in *HCN1* and *MMP16* loci, and are organized by the same way with **Figure S23**.

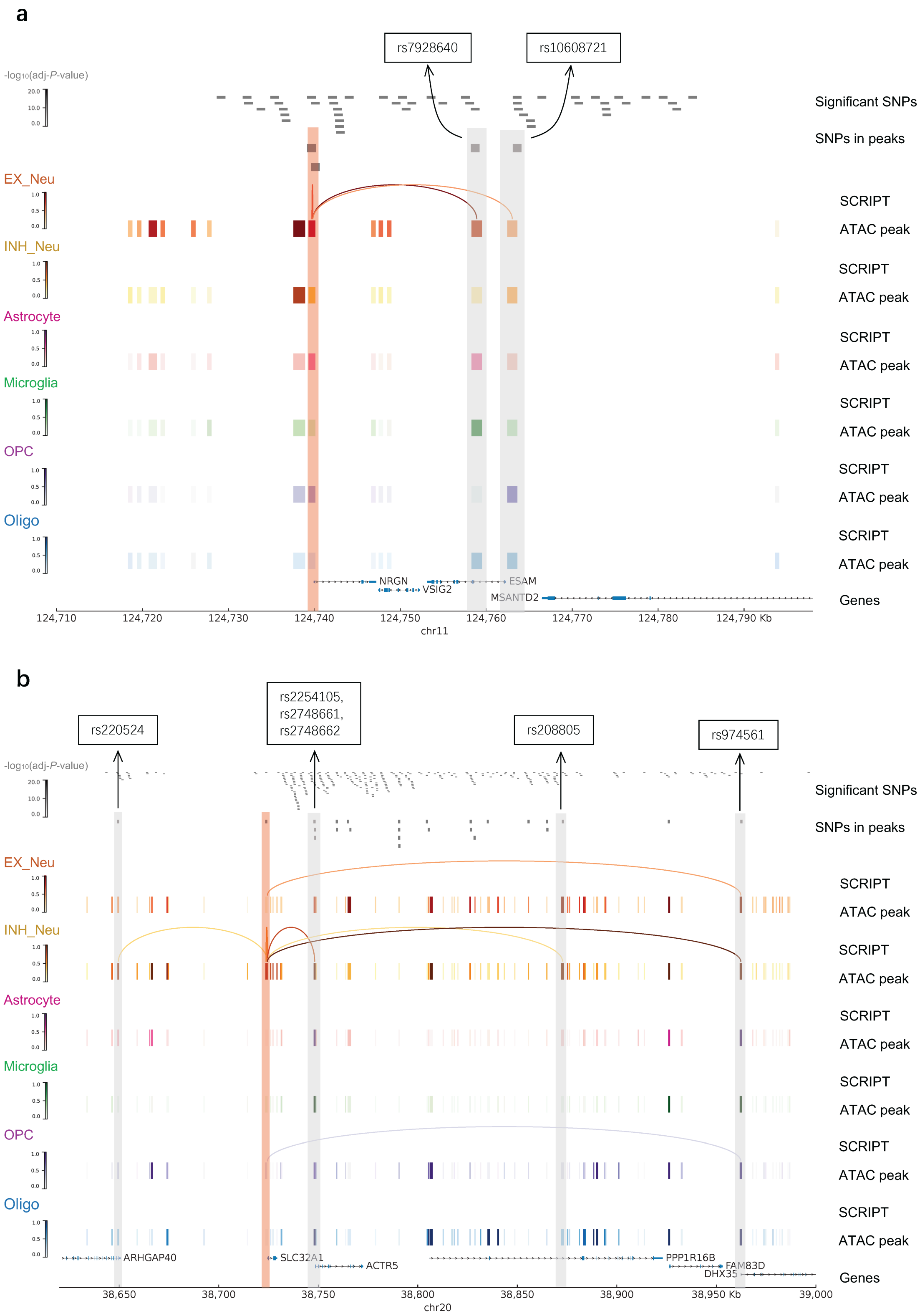

Figure S28 Application of SCRIPT to human cortex dataset for the interpretation of SCZ-related SNPs. Panel **a** and **b** show the results in *NRGN* and *SLC32A1* loci, and are organized by the same way with **Figure S23**.

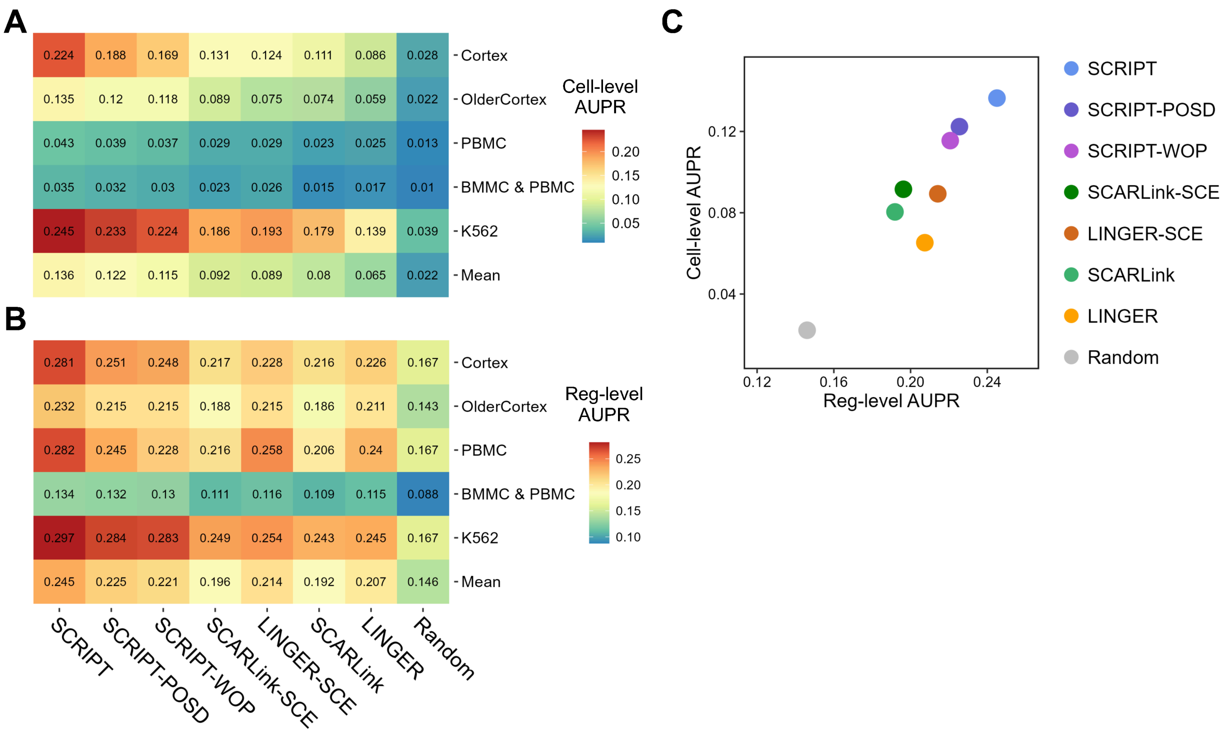

Figure S29 AUPRs of SCRIPT and competing methods for CRR prediction. (A, B) Heatmaps showing cell-level **(A)** and reg-level **(B)** AUPRs of eight methods for single-cell CRR prediction across five benchmark datasets. Method names and dataset names are shown at the bottom and right of the heatmaps, respectively. **(C)** Scatter plot of mean AUPRs for the eight methods, where the x-axis represents the mean reg-level AUPR and the y-axis represents mean cell-level AUPR. Higher-performing methods are expected to appear in the top-right quadrant.

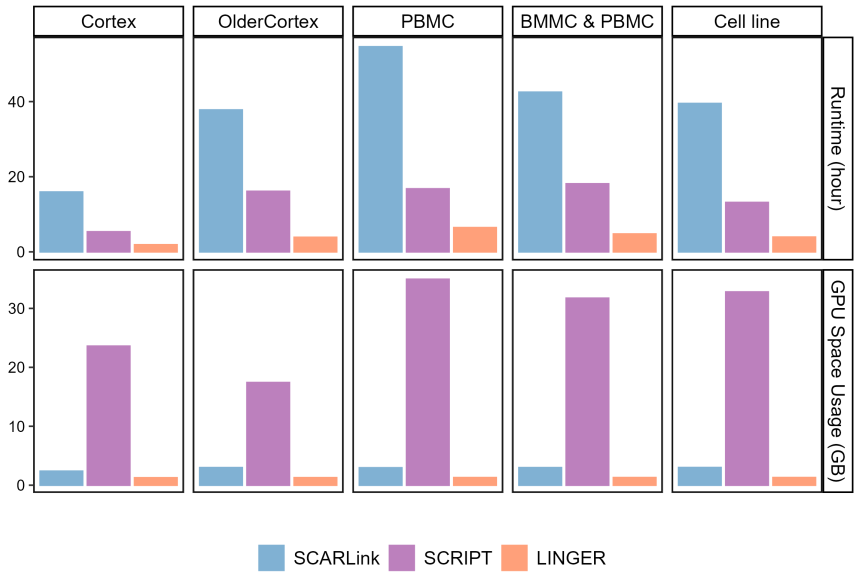

Figure S30 The runtime and space usage analysis of SCARLink, SCRIPT and LINGER across five benchmark datasets. The dataset names are shown in the boxes on top of the bar plots, the evaluation metrics are displayed in the boxes to the right of the bar plots, and different colors represent the various competing methods. All methods were executed on a single NVIDIA A100 GPU.
